# Ocean current patterns drive the worldwide colonization of eelgrass (*Zostera marina*)

**DOI:** 10.1038/s41477-023-01464-3

**Published:** 2023-07-20

**Authors:** Lei Yu, Marina Khachaturyan, Michael Matschiner, Adam Healey, Diane Bauer, Brenda Cameron, Mathieu Cusson, J. Emmett Duffy, F. Joel Fodrie, Diana Gill, Jane Grimwood, Masakazu Hori, Kevin Hovel, A. Randall Hughes, Marlene Jahnke, Jerry Jenkins, Keykhosrow Keymanesh, Claudia Kruschel, Sujan Mamidi, Damian M. Menning, Per-Olav Moksnes, Masahiro Nakaoka, Christa Pennacchio, Katrin Reiss, Francesca Rossi, Jennifer L. Ruesink, Stewart T. Schultz, Sandra Talbot, Richard Unsworth, David H. Ward, Tal Dagan, Jeremy Schmutz, Jonathan A. Eisen, John J. Stachowicz, Yves Van de Peer, Jeanine L. Olsen, Thorsten B. H. Reusch

**Affiliations:** 1 Marine Evolutionary Ecology, GEOMAR Helmholtz Centre for Ocean Research Kiel, Kiel, Germany; 2 Institute of General Microbiology, Kiel University, Kiel, Germany; 3 Department of Paleontology and Museum, University of Zurich, Zurich, Switzerland; 4 Natural History Museum, University of Oslo, Oslo, Norway; 5 Genome Sequencing Center, HudsonAlpha Institute for Biotechnology, Huntsville, AL USA; 6 US Department of Energy Joint Genome Institute, Lawrence Berkeley National Laboratory, Berkeley, CA USA; 7 Department of Evolution and Ecology, University of California, Davis, CA USA; 8 Département des sciences fondamentales, Université du Québec à Chicoutimi, Chicoutimi, Quebec Canada; 9 Tennenbaum Marine Observatories Network, Smithsonian Environmental Research Center, Edgewater, MD USA; 10 Institute of Marine Sciences (UNC-CH), Morehead City, NC USA; 11 Japan Fisheries Research and Education Agency, Yokohama, Japan; 12 Department of Biology, San Diego State University, San Diego, CA USA; 13 Marine Science Center, Northeastern University, Nahant, MA USA; 14 Tjärnö Marine Laboratory, Department of Marine Sciences, University of Gothenburg, Strömstad, Sweden; 15 University of Zadar, Zadar, Croatia; 16 US Geological Survey, Alaska Science Center, Anchorage, AK USA; 17 Department of Marine Sciences, University of Gothenburg, Gothenburg, Sweden; 18 Hokkaido University, Akkeshi, Japan; 19 Nord University, Bodø, Norway; 20 Department of Integrative Marine Ecology (EMI), Stazione Zoologica Anton Dohrn–National Institute of Marine Biology, Ecology and Biotechnology, Genoa, Italy; 21 Department of Biology, University of Washington, Seattle, WA USA; 22 Far Northwestern Institute of Art and Science, Anchorage, AK USA; 23 Department of Biosciences, Swansea University, Swansea, UK; 24 Project Seagrass, the Yard, Bridgend, UK; 25 Center for Population Biology, University of California, Davis, CA USA; 26 Department of Plant Biotechnology and Bioinformatics, Ghent University, Gent, Belgium; 27 Center for Microbial Ecology and Genomics, Department of Biochemistry, Genetics and Microbiology, University of Pretoria, Pretoria, South Africa; 28 College of Horticulture, Academy for Advanced Interdisciplinary Studies, Nanjing Agricultural University, Nanjing, China; 29 VIB-UGent Center for Plant Systems Biology, Gent, Belgium; 30 Groningen Institute for Evolutionary Life Sciences, Groningen, The Netherlands

**Keywords:** Population genetics, Plant evolution, Marine biology

## Abstract

Currents are unique drivers of oceanic phylogeography and thus determine the distribution of marine coastal species, along with past glaciations and sea-level changes. Here we reconstruct the worldwide colonization history of eelgrass (*Zostera marina* L.), the most widely distributed marine flowering plant or seagrass from its origin in the Northwest Pacific, based on nuclear and chloroplast genomes. We identified two divergent Pacific clades with evidence for admixture along the East Pacific coast. Two west-to-east (trans-Pacific) colonization events support the key role of the North Pacific Current. Time-calibrated nuclear and chloroplast phylogenies yielded concordant estimates of the arrival of *Z. marina* in the Atlantic through the Canadian Arctic, suggesting that eelgrass-based ecosystems, hotspots of biodiversity and carbon sequestration, have only been present there for ~243 ky (thousand years). Mediterranean populations were founded ~44 kya, while extant distributions along western and eastern Atlantic shores were founded at the end of the Last Glacial Maximum (~19 kya), with at least one major refuge being the North Carolina region. The recent colonization and five- to sevenfold lower genomic diversity of the Atlantic compared to the Pacific populations raises concern and opportunity about how Atlantic eelgrass might respond to rapidly warming coastal oceans.

## Abstract (web summary)

Ocean currents play a crucial role in the distribution of marine coastal species. Here the nuclear and chloroplast genomes of this eelgrass (*Zostera marina* L.) is used to trace its colonization history from its origin in the Northwest Pacific.

## Main

**Fig. 1:**
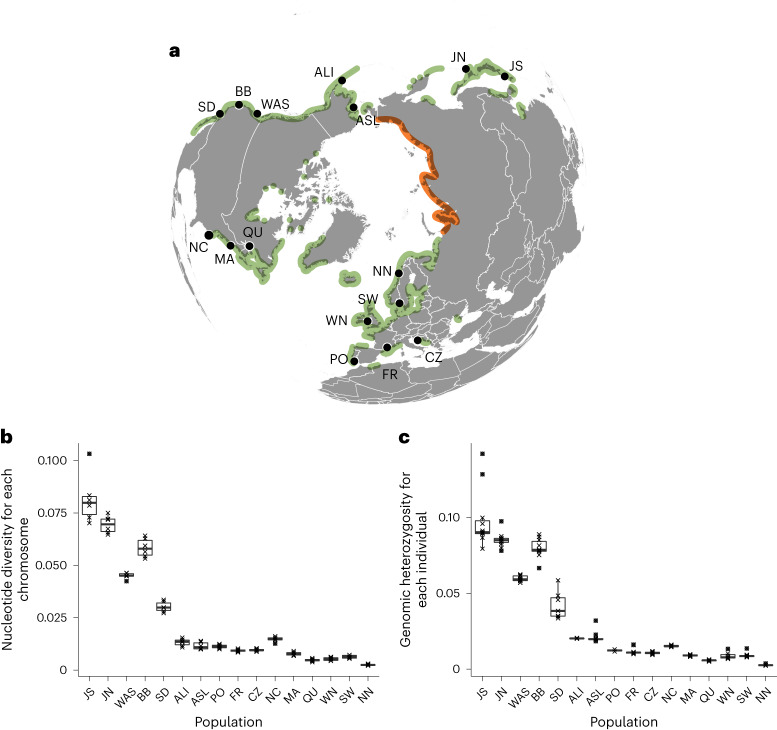
Distribution and sampling sites for *Z. marina* and their widely varying genetic diversity. **a**, Green areas indicate the presence of *Z. marina*. Arctic Canada locations were added from ref. ^76^. The orange line along the Siberian coastline represents the absence of *Z. marina* based on cursory surveys of Alismatales including *Z. marina* by Russian colleagues. The latter areas are characterized by gravel coasts, river outflows and turbid waters. Detailed location metadata can be found in Supplementary Table 1. **b**, Genetic diversity: box plots (median, 25/75% percentile, range whiskers within 1.5× of inter-quartile range, outliers >1.5× of inter-quartile range) of nucleotide diversity (*π*), calculated for each of the six chromosomes based on 44,865 SNPs (Supplementary Fig. 1). Each data point indicates one chromosome. **c**, Box plots of individual genome-wide heterozygosity *H*_obs_ based on 144,773 SNPs (data set ZM_neutral_SNPs; Supplementary Fig. 1), as (number of heterozygous sites)/(total number of sites with genotype calls). Each data point corresponds to an individual (*N* = 2–14 individuals, for exact values see Source Data Fig. 1). Statistical tests for differences in mean *π* or *H*_obs_ are given in Supplementary Table 4. WN, Wales-North; FR, Mediterranean France; CZ, Croatia. Source data

Seagrasses are the only flowering plants that returned to the sea ~67 mya (million years ago). Three independent lineages descended from freshwater ancestors that lived ~114 mya (ref. ^1^). Seagrasses are foundation species of entire ecosystems thriving in all shallow coastal areas of the global ocean except Antarctica^2^. By far the most geographically widespread species is eelgrass (*Zostera marina*), occurring in Pacific and Atlantic areas of the Northern Hemisphere from warm temperate to Arctic environments^3^, spanning 40° of latitude and a range of ~18 °C in average annual temperatures (Fig. 1a). Eelgrass is a unique foundation species in that no other current seagrass can fill its ecological niche in the cold temperate to Arctic Northern Hemisphere^3^ (Supplementary Note 1). At the same time, eelgrass meadows provide critical nursery functions and ecosystem services including erosion protection, nutrient cycling and considerable carbon sequestration^4^.

Given its very wide natural distribution range that exceeds most terrestrial plant species, our goal was to reconstruct the major colonization pathways of eelgrass starting from the putative origin of *Z. marina* in the West Pacific along the Japanese Archipelago^5,6^. Currents are unique drivers of phylogeographic processes in the ocean, and we hypothesized that the North Pacific Current, Alaska and California Currents in the Pacific, and the Labrador, Gulf Stream and North Atlantic Drift in the Atlantic drove its worldwide colonization. Being a flowering plant, rafting seed-bearing shoots of eelgrass stay alive for weeks and have been shown to be able to travel tens to hundreds of kilometres, providing a biological mechanism for long-distance dispersal^7^ (Supplementary Note 1).

One major objective of the present study was to provide time estimates of major colonization events. We asked how evolutionary contingency—specifically the timing of large-scale dispersal events—may have affected the timing of arrival of eelgrass on East Pacific and North Atlantic coastlines^8^. To do so, we took advantage of recent extensions of the multi-species coalescent (MSC) as applied at the population level^9,10^, making it possible to construct a time-calibrated phylogenetic tree from SNP (single-nucleotide polymorphism) data^11^. Our data set comprised 190 individuals from 16 worldwide locations that were subjected to comprehensive whole-genome resequencing (nuclear and chloroplast).

Superimposed on the general eastward colonization are Pleistocene cycles of glacial and interglacial periods that resulted in frequent latitudinal expansions and contractions of available habitat for both terrestrial and marine biota^12^. Such local extinctions and subsequent recolonizations from refugial populations are expected to leave their genomic footprint in extant marine populations^13–15^ and may restrict their potential to rapidly adapt to current environmental change^16,17^. Hence, we were also interested in how glaciations—in particular the Last Glacial Maximum (LGM; 20 kya (thousand years ago); ref. ^18^)—have affected the population-wide genomic diversity of *Z. marina* and which glacial refugia permitted eelgrass to survive this period.

## Results

### Whole-genome resequencing and nuclear and chloroplast polymorphism

Among 190 *Z. marina* specimens collected from 16 geographic locations (Fig. 1a and Supplementary Table 1), full-genome sequencing yielded an average read coverage of 53.73x. After quality filtering (Supplementary Data Table 1), SNPs were mapped and called (Supplementary Figs. 1 and 2) based on a chromosomal-level assembly v.3.1 (ref. ^19^). To avoid reference-related bias, owing to the large Pacific–Atlantic genomic divergence, and to facilitate phylogenetic reconstruction within a conserved set of genes^20^, we focused on core genes—the set of genes shared by most individuals. From a total of 21,483 genes, we identified 18,717 core genes that were on average observed in 97% of the samples, containing 763,580 SNPs (hereafter ‘ZM_HQ_SNPs’; Supplementary Note 2).

After exclusion of 37 samples owing to missing data, selfing or duplicate clonality, 153 were left for further analyses (Supplementary Tables 2 and 3 and Supplementary Figs. 3 and 4). We also extracted two additionally filtered SNP data sets: one based on synonymous SNPs (‘ZM_neutral_SNPs’, comprising 144,773 sites) and the other based on a further subset in which only sites with a physical distance of >3 kbp were retained (‘ZM_Core_SNPs’, 11,705 SNPs; Supplementary Figs. 1 and 2; see Methods for further explanation).

A complete chloroplast genome of 143,968 bp was reconstructed from the reference sample^21^. Median chloroplast sequencing coverage for the samples of the worldwide data set was 6,273x. A total of 151 SNPs were detected along the whole chloroplast genome, excluding 23S and 16S ribosomal RNA gene regions due to possible contamination in some samples and ambiguous calling next to microsatellite regions (132,438 bp), comprising 54 haplotypes.

### Gradients of genetic diversity within and among ocean basins

As measures of genetic diversity, we assessed nucleotide diversity (*π*) and genome-wide heterozygosity (*H*_obs_) (Fig. 1b,c). Consistent with the Pacific origin of the species (Supplementary Note 3), Pacific locations showed a 5.5 (*π*)- to 6.6 (*H*_obs_)-fold higher genetic diversity compared to the Atlantic ones (Supplementary Table 4). The highest *π* and *H*_obs_ values were observed in Japan-South (JS) followed by Japan-North (JN). Alaska-Izembek (ALI) and Alaska-Safety Lagoon (ASL) showed approximately a third (28% for *π*; 34% for *H*_obs_) of the diversity in the more southern Pacific sites (average of San Diego (SD), Bodega Bay, California (BB) and Washington State (WAS)). In the Atlantic, a comparable loss of diversity along a south–north gradient was observed. Quebec (QU) showed 42% (*π*) and 47% (*H*_obs_) of the diversity of North Carolina (NC) and Massachusetts (MA), while the diversity values in Northern Norway (NN) was 31% and 43% of averaged values of Sweden (SW) and Wales, respectively.

### Global population structure of *Z. marina*

**Fig. 2:**
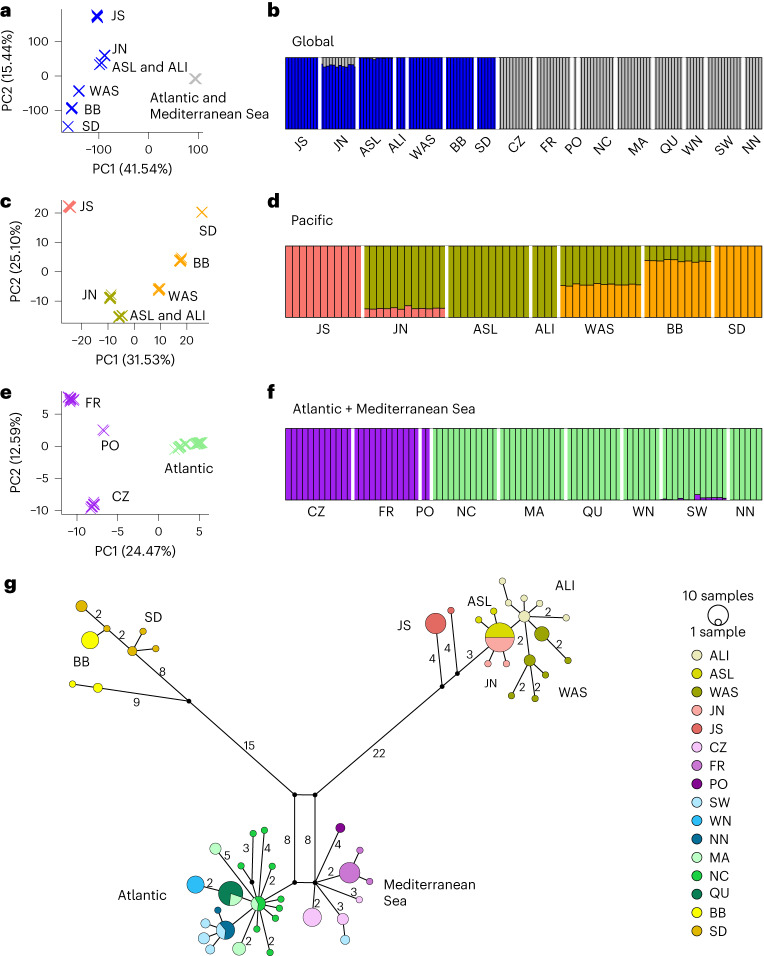
Population structure based on nuclear and cpDNA SNPs among 16 eelgrass populations. **a**,**b**, Global genetic population structure. **a**, Global PCA based on data set ZM_HQ_SNP (763,580 sites); the Atlantic and Mediterranean populations are collapsed. The Pacific and Atlantic Ocean were separated by principal component 1 (PC1) that explained 41.75% of the variation. **b**, Global STRUCTURE analysis (number of clusters, *K* = 2; based on 2,353 SNPs). Each individual is represented by a vertical bar partitioned into colours based on its affiliation to a genetic cluster (Methods). **c**,**d**, Genetic population structure within the Pacific. **c**, PCA within the Pacific based on 12,514 SNPs. **d**, STRUCTURE analysis within the Pacific (*K* = 3; 6,168 SNPs). **e**,**f**, Genetic population structure for the Atlantic and the Mediterranean Sea. **e**, PCA for the Atlantic and the Mediterranean Sea based on 8,552 SNPs. **f**, STRUCTURE analysis for the Atlantic and the Mediterranean Sea (*K* = 2; 8,552 SNPs). See Supplementary Figs. 5–7 for results and discussion assuming higher numbers of clusters and Supplementary Fig. 1 for further details on the SNP sets used. **g**, cpDNA haplotype network. Numbers represent mutation steps >1. Colours correspond to the population. Split-coloured circles indicate that a particular haplotype is shared between populations; the circle size is proportional to the frequency. PC2, principal component 2.

To reveal the large-scale population genetic structure, we performed a principal component analysis (PCA) based on the most comprehensive SNP selection (Supplementary Fig. 1; 782,652 SNPs, Fig. 2a). Within-ocean genetic differentiation in the Pacific was as great as the Pacific–Atlantic split, whereas there was much less variation within the Atlantic. Separate PCAs for each ocean revealed additional structure (Fig. 2c,e), including the separation of the Atlantic and Mediterranean Sea populations (principal component 1, 24.47%, Fig. 2e).

We then used STRUCTURE^22^, a Bayesian clustering approach, on 2,353 SNPs (20%) randomly selected from the ZM_Core_SNPs. The most likely number of genetic clusters was determined using a combination of the Delta-*K* method^23^ and other metrics introduced by ref. ^24^ (Fig. 2b,d,f), with a qualitative inspection of additional *K* values as generated from StructureSelector^25^ in Supplementary Figs. 5–7. In the global analysis (Fig. 2b), two clusters representing Atlantic and Pacific locations were identified. JN contained admixture components with the Atlantic, consistent with a west–east colonization via northern Japan through the North Pacific Current and then north towards the Bering Sea. Given the pronounced nested population structure (Fig. 2a), we then proceeded with separate analyses for Pacific and Atlantic, as recommended in ref. ^25^. An analysis restricted to Pacific sites supported a role of JN as a dispersal hub, with admixture components from JS and Alaska, suggesting that this site has been a gateway between both locations (Fig. 2c). At *K* = 3, WAS and BB, located centrally along the east Pacific coastline, were admixed between both Alaskan sites and SD. WAS showed about equal northern and southern components, while BB was dominated by the adjacent southern SD genetic component. Interestingly, under *K* = 4 (Supplementary Fig. 6), which was supported by the metrics medmeak and maxmeak^24^, a presumably ancient connection between JN and SD becomes apparent, while at even larger *K* values, the pattern remains stable for the Pacific side.

In the Atlantic and Mediterranean (Fig. 2f), a less pronounced population structure was present, with only two clearly separated groups representing the Mediterranean (plus Portugal (PO)) and all other Atlantic Ocean sites (both east and west), consistent with the PCA results (Fig. 2e). Further exploration of an additional genetic cluster revealed a connection between PO closest to the Strait of Gibraltar and the East Atlantic at *K* = 4 (NC, Supplementary Fig. 7, supported by medmeak and maxmeak). A clear split among West and East Atlantic becomes apparent with *K* = 4 and 5 clusters, for which either the separation time since the LGM or some non-sampled East Atlantic refugia might be responsible.

### Population structure of chloroplast DNA

**Extended Data Fig. 1:**
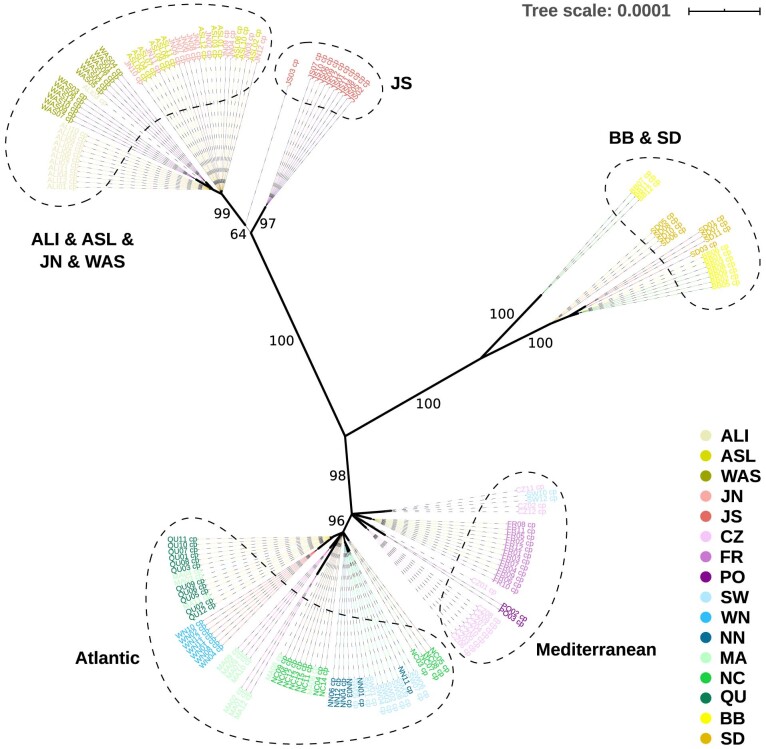
Maximum-likelihood phylogenetic tree based on cpDNA polymorphism. Based on fully sequenced chloroplast genomes, 108 parsimony-informative SNPs present in at least two samples were included. The tree topology supports the haplotype network topology with high bootstrap values. The tree was reconstructed by IQ-TREE v1.5.5 with 1000 bootstrap runs^32^ and visualized by iTOL (ref. ^78^).

A haplotype network (Fig. 2g) revealed three markedly divergent clades, which were additionally supported by bootstrap values of 98–100% based on a maximum-likelihood phylogeny (Extended Data Fig. 1). In the Pacific, WAS showed haplotypes similar to those of Alaska (ALI and ASL) and JN, while BB showed haplotypes of a divergent clade that also comprises all haplotypes from SD. ASL and JN share the same dominant haplotype, suggesting JN to be a hub between West and East Pacific. In JS, two divergent private haplotypes (separated by nine mutations from other haplotypes) suggest long-term persistence of eelgrass at that location.

On the Atlantic side, only four to six mutations separate the Northeast Atlantic and Mediterranean haplotypes, consistent with a much younger separation. The central (putatively ancestral) haplotype is shared by both MA and NC, with nine private NC haplotypes. A single mutation separates both MA and QU, as well as MA and Wales-North. Also extending from the central haplotype were SW and NN (Fig. 2g). Together with the diversity measures (Fig. 1b,c), this pattern suggests long-term residency of eelgrass on the West Atlantic coast and transport to the Northeast Atlantic via the North Atlantic Drift. Notably, there were no shared chloroplast DNA (cpDNA) haplotypes among Pacific and Atlantic, suggesting that the Atlantic was colonized only once.

### Reticulated topology of *Z. marina* phylogeography

**Fig. 3:**
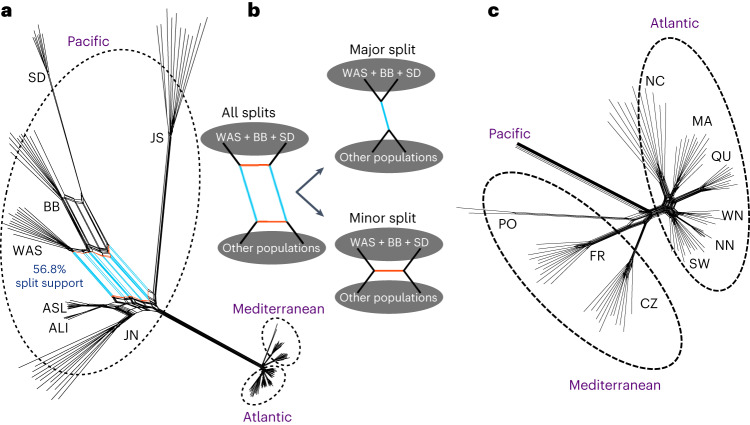
Conflicting phylogenetic signals in the nuclear genome. **a**, Splits network based on the ZM_Core_SNP data set (11,705 sites; Supplementary Fig. 1). Each terminal branch indicates one individual sample. Splits coloured in cyan are particularly strongly supported between a grouping of WAS, BB and SD and the rest of the Pacific. **b**, Main signals in the observed network structure. The splits network structure indicates that the SNP data set supports alternative evolution histories, which are particularly strong with respect to BB, WAS and SD. The major split depicted in **b** is supported by 56.8% of all splits. **c**, Splits network reconstructed for Atlantic populations only.

To further explore the degree of admixture and secondary contact, we constructed a split network^26^ using all ZM_Core_SNPs. Pacific populations were connected in a web-like fashion (Fig. 3a). WAS and BB were involved in alternative network edges (Fig. 3b), either clustering with SD or with both JS and JN. The topology places WAS and BB in an admixture zone with a northern Alaska component (ALI and ASL) and a more divergent southern component from SD, in line with the STRUCTURE results (Fig. 2c). Due to uniparental inheritance mode, the population relationships inferred from chloroplast data were expected to reflect only one of the two topologies. Based on these data, WAS groups with the Alaska component (Fig. 2g and Supplementary Fig. 6), indicating an early divergence from the SD and BB cpDNA haplotypes. In the Atlantic (Fig. 3c), edges among locations were shorter than those on the Pacific side, indicating a more recent divergence among Atlantic populations. A bifurcating topology connected the older Mediterranean populations, while both Northeast and Northwest Atlantic were connected by unresolved, web-like edges, indicating a mixture of incomplete lineage sorting and probable, recent gene flow.

**Extended Data Fig. 2:**
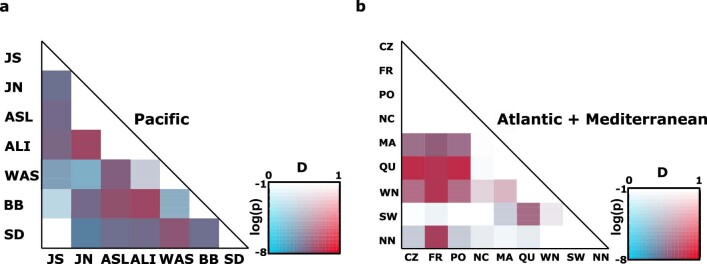
Matrix depicting Patterson’s D-statistic for Pacific and Atlantic populations separately. D-statistic values (aka ABBA-BABA statistics) are presented as two-color heat map, with red intensity indicating higher D-values. More saturated colors towards the lower edge of the color legend, indicate increasing statistical significance (log(*p*)). Significant and high D-values indicate admixture between any two population pairs, but the direction of gene flow cannot be estimated. A signal of admixture can be caused by direct gene flow between the two populations or by genetic input from a third unsampled population.

We used Patterson’s *D*-statistic^27^ to further test for admixture^28^ (Extended Data Fig. 2). For the Pacific side, the pairs WAS/SD, BB/ALI and BB/ASL in addition to JN/ALI and JN/ASL showed the highest *D* values along with statistical significance (*D* = 0.67; *P* < 0.001), suggesting substantial admixture. For the Atlantic side, *D* values indicated recent or ongoing connection between the Atlantic and Mediterranean Sea, consistent with the admixture signal detected by STRUCTURE (SW, Fig. 2f) and with two Atlantic (SW) cpDNA haplotypes that cluster with the Mediterranean ones (Fig. 2g).

### Time-calibrated MSC analysis of colonization events

**Fig. 4:**
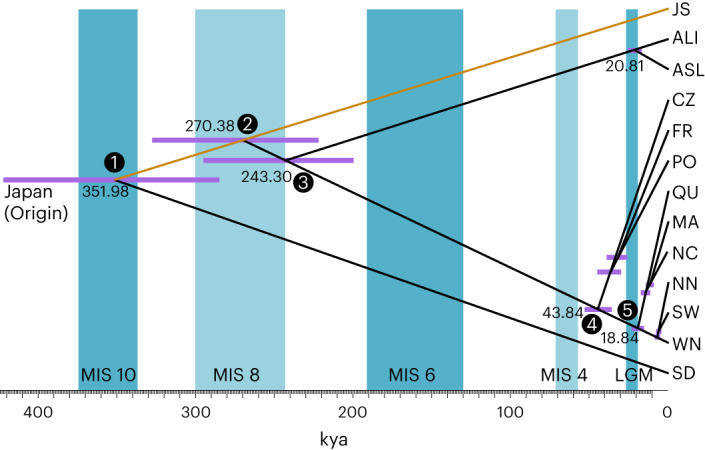
Time-calibrated phylogenetic tree based on the MSC allows dating of major colonization events. Blue bars indicate glacial periods with Marine Isotope Stages (MIS) alternating with warm to cool interglacial periods (white). The intensity of blue colour depicts the intensity of glaciations. The LGM (MIS2 = LGM) is depicted at 26.5–19 kya. Estimated absolute divergence times of nodes along with 95% confidence intervals (highest posterior densities, purple bars) are given. The three most strongly admixed populations WAS, BB and JN were excluded (Figs. 2 and 3). The orange edge connects the hypothetical founder in the Japan area with the extant JS. Inferred colonization scenarios (numbered black dots on the nodes) are presented in Fig. 6. ASTRAL- and StarBEAST2-based divergence time estimates are presented in Supplementary Fig. 12.

Application of the MSC^11^ (Fig. 4) assumes that populations diverge under a bifurcating model. Hence, three locations (WAS, BB, JN) that showed pronounced admixture (compare with Figure 2; Extended Data Fig. 2) were excluded, while we explored the effects of including or excluding admixed populations in Supplementary Fig. 9.

As direct fossil evidence is unavailable within the genus *Zostera*, the divergence time between *Z. marina* and *Zostera* *japonica* was estimated from a calibration point that takes advantage of a whole-genome duplication event previously identified and dated to ~67 mya (ref. ^21^). The resulting clock rate for fourfold degenerative transversions of paralogous gene sequences yielded a divergence time estimate of 9.86–12.67 mya between *Z. marina* and *Z. japonica* (Supplementary Note 4). We then repeated the analysis based on 13,732 SNP sites polymorphic within our target species (Supplementary Fig. 2) after setting a new *Z. marina*-specific calibration point.

Assuming JS as generally representative of the species origin^5^ (Supplementary Note 3), we found evidence for two trans-Pacific dispersal events (Fig. 4). The first trans-Pacific dispersal event at ~352 kya (95% highest posterior density (HPD), 422.10–284.9 kya) founded populations close to SD that remained isolated but engaged in admixture to the north (Supplementary Note 5), as also supported by chloroplast-based population structure. A second trans-Pacific dispersal event from JS to the Northeast Pacific seeded the Alaskan populations some 270 kya (95% HDP, 327.50–221.8 kya), likely with JN as stepping stone. Shortly thereafter, the Atlantic was colonized ~243 kya (95% HPD, 294.9–199.6 kya) from populations in or close to Alaska. This estimate is surprising given that the Bering Strait opened as early as 4.8–5.5 mya (ref. ^29^). Further support for JN being a dispersal hub is its smallest pairwise *F*_ST_ with all Atlantic populations (Supplementary Table 5). Moreover, JN was the only Pacific population that showed a shared genetic component with the Atlantic (Fig. 2b).

In the Atlantic, divergence time estimates were much more recent than in the Pacific. The Mediterranean Sea clade emerged ~43.8 kya (95% HPD, 52.8–35.5 kya). The Northwest and Northeast Atlantic populations also diverged from each other very recently at ~18.8 kya (95% HPD, 22.9–15.1 kya) and shared a common ancestor during the LGM, indicating that they were partially derived from the same glacial refugium in the Northwest Atlantic (likely at or near NC). Some admixture found in the SW population stemming from the Mediterranean gene pool (Fig. 2f, g) likely explains a higher genetic diversity at that location (Fig. 1b,c). Some coalescence runs of the population data set with WAS, BB and JN excluded showed a different topology for the JS–Alaska–Atlantic split, requiring the presence of a third trans-Pacific colonization event that predated the Atlantic colonization (Supplementary Fig. 9a), along with a more recent dispersal to Alaska. Note that divergence time estimates for all other splits, in particular the foundation of the SD lineage and the Atlantic and Mediterranean colonization, were very similar.

In a second coalescent approach^10^, we used alignments of 617 core genes across all samples (Supplementary Note 2). Based on the same initial calibration as under the MSC, the tree topology was examined using ASTRAL. Despite high incomplete lineage sorting (ASTRAL normalized quartet score = 0.48), the species tree follows geographic patterns with only 2 of 107 individuals showing incongruent topology based on geographic collection sites^30^ (Supplementary Fig. 11). Subsequent divergence time estimation was performed with StarBEAST2 (ref. ^31^). This approach resulted in a topology consistent with the one depicted in Fig. 4, while divergence time estimates for the deeper nodes were even more recent (for example, Pacific–Atlantic split at 162 kya). Estimates for the more recent divergence events were nearly identical (Supplementary Fig. 12). The StarBEAST2-based topology supports the SNAPP topology presented in Fig. 4.

Finally, we used the mutational steps among chloroplast (cpDNA) haplotypes as an alternative dating method. SD and BB along the Pacific East coast showed very different haplotypes, separated by about 30 mutations from the other Pacific and the Atlantic clades. Assuming a synonymous cpDNA mutation rate of 2 × 10^−9^ per site per year, this genetic distance corresponds to a divergence time of 392 kya (Supplementary Note 6), comparable to the estimate of 352 kya in the coalescent analysis. Conversely, few mutations (4–7) distinguished major Atlantic haplotypes from the Mediterranean Sea, consistent with a much younger divergence estimate based on nuclear genomes (Fig. 4). The topology had a high bootstrap support in a maximum-likelihood-based phylogenetic tree^32^ (Extended Data Fig. 1).

### Demographic history and post-LGM recolonization

**Fig. 5:**
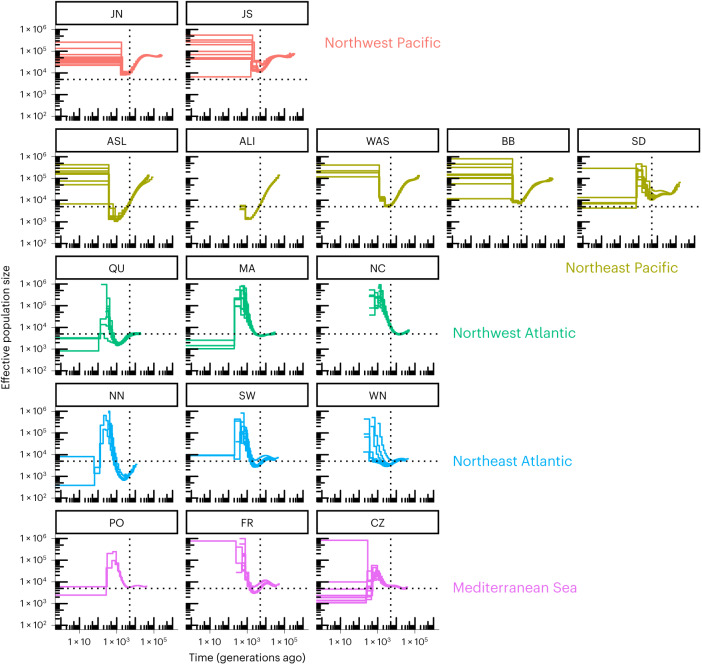
Demographic history of worldwide eelgrass (*Z. marina*) populations reveal effects of the last glacial maximum (LGM). Historical effective population sizes (*N*_e_) were inferred by the MSMC. Replicate runs were performed with all unique genotypes in each location, depicted as separate lines. The *x* axis depicts generations rather than absolute time as generation time for *Z. marina* varies depending on the level of local clonality. *N*_e_ values are capped at 1 million. Many populations reveal a minimal *N*_e_ (thus likely a bottleneck) at ~5,000 generations ago (dashed vertical lines), which probably reflects the impact of the LGM if we estimate a mean generation time of ~3 years. Note that demographic estimates of <1,000 generations are considered unreliable and are hence not interpreted. Dashed horizontal lines at *N*_e_ = 5,000 are for orientation only.

We used the multiple sequentially Markovian coalescent (MSMC)^33^ to infer past effective population size *N*_e_ (Fig. 5). We here focus on time intervals where different replicate runs per population converged, acknowledging that MSMC creates unreliable estimates in recent time^34^. Almost all eelgrass populations revealed a recent expansion 1,000–100 generations ago, while the magnitude of *N*_e_ value minima (at about 10,000–1,000 generations) varied. Given a range of plausible generation times under a mix of clonal and sexual reproduction, it is likely that an *N*_e_ minimum shown by several locations coincides with the LGM, which in turn can be used to estimate the long-term generation time. For example, a local minimal *N*_e_ at 5,000 generations ago, at locations JS, WAS, BB, SD and MA would translate to 3 year × generation^−1^ × 5,000 generations = 15 kya, just after the LGM. In general, lower *N*_e_ values were related to lower clonal diversity at sites in northern (NN) and southern Europe (PO; Supplementary Table 3). Within the Pacific, the southernmost population (SD) showed no drop in *N*_e_, while all others showed bottlenecks that became more pronounced from south to north (in the order BB, WAS and ALI/ASL). As for the Atlantic side, the Northwest Atlantic populations NC and MA and the southern European populations PO and CZ (and to a lesser extent Mediterranean FR) showed little evidence for bottlenecks (as local *N*_e_ minima), suggesting that these localities were refugia during the LGM (Fig. 5). The opposite applied to QU in the Northwest and NN and SW in the Northeast Atlantic, where we see a pronounced minimal *N*_e_ at about 3,000 generations ago.

For the Atlantic, we determined the most likely post-LGM recolonization through approximate Bayesian computations (Do-It-Yourself-Approximate Bayesian Computation - DIY-ABC; Supplementary Fig. 10) and found that the region north from NC to QU was the most likely donor source (Supplementary Note 7).

## Discussion

**Fig. 6:**
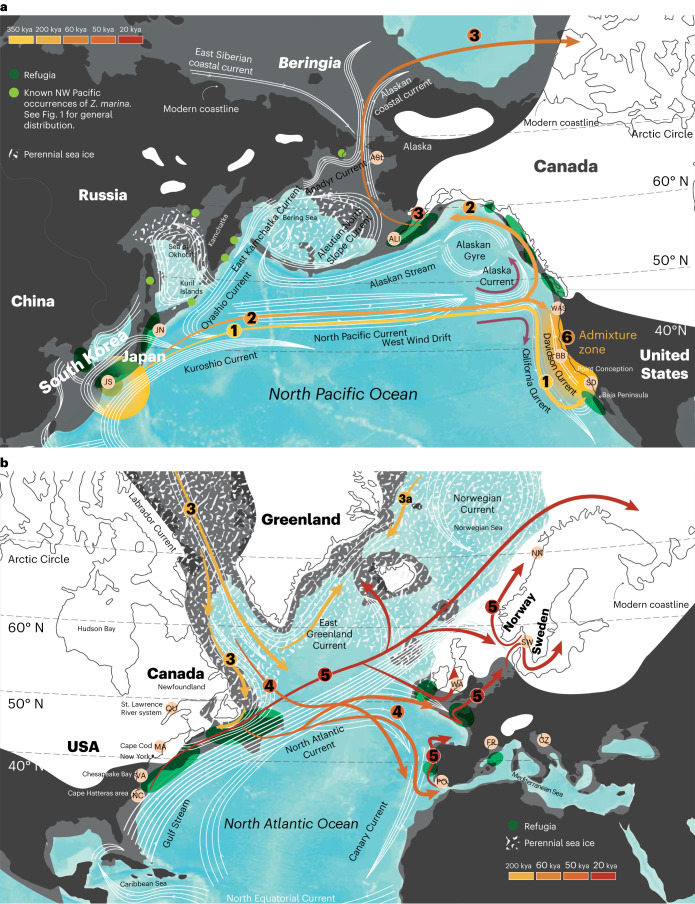
Dispersal and colonization history across the Pacific and to the Atlantic. **a**, Pacific Ocean. *Z. marina* arose in the Japanese Archipelago region (Supplementary Note 3). Known occurrences in the Russian Arctic are depicted by light green dots. Hypothesized dispersal events are as follows: event 1, first trans-Pacific dispersal via the North Pacific Current, arriving at the North Pacific Current ‘gateway’, where it splits both south following the California Current and north via the Alaska Current, in this case taking the southern route (Supplementary Note 5); event 2, later trans-Pacific dispersals to the ‘gateway’ go south or north and, if the northern route, then up to Alaska and possibly beyond; event 3, colonization of the Atlantic from recently colonized Alaska. Admixture zone along the Pacific East coast (event 6): SD ancestors may have later dispersed northwards via the seasonally reversing, near-shore Davidson Current, forming sequential admixtures with BB and WAS. **b**, Atlantic Ocean. The dispersal into the Atlantic was likely propelled by the southward Labrador current (event 3) providing the original foundation and subsequent spread to the Mediterranean (including southern Portugal) and dispersal further along both Atlantic coastlines between MIS4 and the LGM (Supplementary Note 7) in which expansions and contractions of populations moved with the ice edge and changing sea level. Event 4 describes the early colonization of the Mediterranean Sea before the LGM. Event 5 represents the post-LGM recolonization in which the West Atlantic refugia close to NC (along with a hypothesized southern European refugia) created the distribution we see today^46,77^. For both maps: black, present coastline; dark grey, LGM sea level coastline; white, glaciers; speckled white, perennial sea ice; as shown, current pathways. Pink dots with labels following Fig. 1, sampled locations; dark green ovals, hypothesized refugia; yellow–orange–red gradient arrows, dispersal pathways and timing including the North Pacific Current ‘gateway’ (paired purple arrows) and Davidson Current (white). Numbers on current pathways correspond to phylogenetic branch points (nodes) in Fig. 4.

With rapid climate change, information about past climatic shifts and their legacy effects on genetic structure and diversity of extant populations can help to guide restoration efforts to ensure persistence and resilience^16,17,35^. *Z. marina* has a circumglobal distribution that provided us with the unique opportunity to reconstruct the natural expansion of a marine plant throughout the Northern Hemisphere starting from the species origin in the Northwest Pacific during a period of strong recurrent climate changes (Fig. 6a,b).

The presence of eelgrass in the Atlantic is surprisingly recent, dating to only ~243 kya. As no other seagrass species is able to fill this ecological niche or form dense meadows in boreal to Arctic regions (>50° N, Supplementary Note 1), historical contingency^8^ has played a previously underappreciated role for the establishment of this unique and productive ecosystem. The recency of the arrival of eelgrass in the Atlantic may also explain why relatively few animals are endemic to eelgrass beds or have evolved to consume its plant tissue directly (Supplementary Table 6). Greater numbers of species are found to be intimately associated with *Z. marina* in the Pacific than the Atlantic, including specialist feeders, facultative feeders on green tissue and habitat specialists.

The first dated population-level phylogeny in any seagrass species might also explain why there seems to be little niche differentiation among eelgrass-associated epifauna in the Atlantic compared to the Pacific^36^. Our study shows how macro-ecology, here the presence of an entire ecosystem, may be strongly determined by the colonization history, specifically the time frame in which eelgrass reached the North Atlantic^8^, and not by suitable environmental conditions.

We identified the North Pacific Current, which began to intensify ∼1 mya (ref. ^37^), as the major dispersal gateway. It bifurcates north into the Alaska Current and south into the California Current (Fig. 6a), roughly at the latitude of mid-Vancouver Island (Supplementary Note 5). Based on this scenario, SD was colonized by the earliest detectable colonization event roughly 352 kya (Fig. 6a, event 1) and has retained old genetic variation since then, probably owing to the rarity of genetic exchange southward across the Point Conception biogeographic boundary^38^ and the variable North–South Davidson Current (reviewed in ref. ^39^). Subsequent trans-Pacific events that headed south at the gateway eventually resulted in an admixture zone involving WAS and BB.

Another trans-Pacific dispersal (Fig. 6a, event 2) at 270 kya moved north through the gateway, colonized Alaska and became the stepping stone for an inter-oceanic dispersal to the Atlantic through the Arctic Ocean some 243 kya (event 3). Further support for the gateway bifurcation comes from two chloroplast *mat*-K haplotypes present in northern Hokkaido, Japan^40^, with a split on the East Pacific side: the *mat*-K2 haplotype went north and was found at 12 sites in the Bering and Gulf of Alaska Large Marine Ecosystems, whereas the *mat*-K4 haplotype was found south of the gateway at six sites in the California Current Large Marine Ecosystem all the way to Baja (Supplementary Note 5).

Although the Bering Strait may have opened as early as 5.5–4.8 mya (ref. ^29^), our analyses only support a single colonization event into the Atlantic, in contrast to findings for other amphi-Arctic and boreal marine invertebrates^41^ and seaweeds^42^. Genomic variation characteristic of extant Alaskan populations was not detected in any North Atlantic populations, in line with earlier microsatellite data^40^, corroborating that the Atlantic was only colonized once. While we cannot rule out an earlier colonization, this would require that *Z. marina* became extinct without leaving any trace in nuclear genomes or cpDNA haplotypes, which we consider unlikely.

The Pacific–Atlantic genetic divide has been recently identified as a ‘Pleistocene legacy’ based on a microsatellite-based genotyping study^17^. Here we further confirm the presence of two deeply divergent clades in the Pacific that share a complex pattern of secondary contact on the East Pacific side (Supplementary Note 8). In contrast, the genetic separation between West and East Atlantic populations is present but weak, suggesting recent population contractions and expansions driven by the LGM, with the North Atlantic Drift driving repeated west–east colonization events (Fig. 6b).

While our phylogeny (Fig. 4) is also consistent with a scenario in which the deep branching SD population would represent the species’ origin of *Z. marina*, we consider this extremely unlikely given the long-term prevailing ocean currents (Fig. 6a), the distribution of genetic diversity (Fig. 1b,c) and our current understanding of the emergence of the genus *Zostera* (~15 mya), including the species *Z. marina* some 5–1.62 mya (ref. ^5^) in the Northwest Pacific (Supplementary Note 3). Thus, considering all evidence jointly, we conclude that the Japan region, and not the East Pacific (SD), is the most likely geographic origin of eelgrass and the source of multiple dispersal events with ocean currents.

The NC and Chesapeake Bay region northward to Long Island served as a major refuge and was at least one subsequent source population for the Northeast Atlantic (Fig. 6b, event 5). The coastal areas further north of Cape Cod, Nova Scotia, Quebec and Newfoundland are also known refugia^43^ and connected by Quebec in our sampling. Additional inclusion of populations from Newfoundland and southern Greenland may modify this view, as may be the case of refugia around southwestern Ireland and the Brittany peninsula^44,45^ (Supplementary Note 7). Indeed, there is some evidence in our data from the STRUCTURE analysis of higher *K* modes (Supplementary Figs. 5–7) and admixture signals in SW that additional East Atlantic refugia resulted in a more complex post-LGM genetic composition of extant northern European populations as suggested earlier^46^ (Supplementary Note 7).

Along with demographic modelling, we identify population contraction and subsequent latitudinal expansion along three coastlines following the LGM (26–19 kya). These are common patterns of many terrestrial^12^ and intertidal species^15,46^, with the Northeast Atlantic/North Sea coastline and Beringia being most drastically affected. Interestingly, for *Z. marina*, the Atlantic region was not more severely influenced by the last glaciations and sea-level changes than the East Pacific (Fig. 5 and 6b), even when considering their relative baseline diversities (Supplementary Table 4). In both oceans, there were dramatic losses of genome-wide diversity. The 5- to 7-fold lower overall genetic diversity in the Atlantic simply amplified LGM effects and resulted in >30-fold differences among populations with the highest (JS) versus lowest (NN) diversity. This observation may have significant but as yet unknown consequences for the adaptive potential and genetic rescue of eelgrass in the Anthropocene.

In conclusion, the relatively low number of extant seagrass species (~65 species in six families^47^) has been attributed to frequent intermediate extinctions^6^. Our data suggest a second plausible process, namely multiple long-distance genetic exchanges within and among ocean basins that may have impeded allopatric speciation (see also ref. ^48^). Our range-wide sampling has allowed an overview of evolutionary history in this lineage of seagrass and opens the door for exploration of functional studies across ocean basins and coasts. Future work will explore the pan-genome of *Z. marina* with the consideration of how the high diversity and robustness of Pacific populations may be able to contribute to management and rescue of populations along rapidly warming Atlantic coastlines.

## Methods

### Study species and sampling design

Eelgrass (*Z. marina* L.) is the most widespread seagrass species of the temperate to Arctic Northern Hemisphere^3^. It is being developed as a model for studying seagrass evolution and genomics^17,19,21,49^. *Z. marina* is a foundation species of shallow water ecosystems^17^ with a number of critical ecological functions including enhancement of fish and crustacean recruitment^50^, improvement of water quality^51^ and the sequestration of ‘blue carbon’^52,53^.

Eelgrass features a mix of clonal (=vegetative spread of the rhizome system) and sexual reproduction via seeds, with varying proportions across locations^46^. The mating system is monoecious. While there is the possibility for selfing, that is, self-compatibility^54^, most populations are outcrossing^55^. Except for the most extreme cases of mono-clonality^56,57^, replicated modular units (leaf shoots = ramets) stemming from a sexually produced individual (=genet or clone) are intermingled to form the seagrass meadow. This also implies that generation times are difficult to estimate or average across populations. Nevertheless, we assumed here based on personal observations that in perennial eelgrass populations, individuals become reproductive in year 2 after germination, while attaining their maximal reproductive output in year 3. Extended clone longevity results in overlapping generations, but not in longer generation times. Additional evidence for an average generation time of 3 years used here for later modelling comes from the historical demographic analysis (Fig. 5), specifically the local *N*_e_ minima that are indicative of the population bottleneck during the LGM.

We conducted a range-wide sampling collection of 190 *Z. marina* specimens from 16 geographic locations (Fig. 1a and Supplementary Table 1). The chosen populations feature a mix of sexual and vegetative reproduction with the exception of mostly vegetative reproduction at the sites PO and NN, apparent through extended clones. Chosen locations were a subset of the *Zostera* Experimental Network sites that were previously analysed using 24 microsatellite loci^17^. Although a sampling distance of >2 m was maintained to reduce the likelihood of collecting the same genet/clone twice, this was not always successful (compare with Supplementary Table 3) and thus provided an estimate of local clonal diversity.

Plant tissue was selected from the basal meristematic part of the shoot after peeling away the leaf sheath to minimize epiphytes (bacteria and diatoms), frozen in liquid nitrogen and stored at −80 °C until DNA extraction.

### DNA extraction, whole-genome resequencing and quality check

About 100–200 mg fresh weight of basal leaf tissue, containing the meristematic region, was ground in liquid N_2_. Genomic DNA was extracted using the Macherey-Nagel NucleoSpin plant II kit following the manufacturer’s instructions. DNA concentrations were in the range of 50–200 ng µl^−1^. Quality control was performed following Joint Genome Institute guidelines (https://jgi.doe.gov/wp-content/uploads/2013/11/Genomic-DNA-Sample-QC.pdf). Plate-based DNA library preparation for Illumina sequencing was performed on the PerkinElmer Sciclone NGS robotic liquid handling system using Kapa Biosystems library preparation kit. About 200 ng of sample DNA was sheared to a length of around 600 bp using a Covaris LE220 focused ultrasonicator. Selected fragments were end-repaired, A-tailed and ligated with sequencing adaptors containing a unique molecular index barcode. Libraries were quantified using KAPA Biosystems’ next-generation sequencing library qPCR-kit on a Roche LightCycler 480 real-time PCR instrument. Quantified libraries were then pooled together and prepared for sequencing on the Illumina HiSeq2500 sequencer using TruSeq SBS sequencing kits (v4) following a 2 × 150 bp indexed run recipe to a targeted depth of approximately 40x coverage. The quality of the raw reads was assessed by FastQC (https://www.bioinformatics.babraham.ac.uk/projects/fastqc/) and visualized by MultiQC^58^. BBDuk (https://jgi.doe.gov/data-and-tools/bbtools/bb-tools-user-guide/bbduk-guide/) was used to remove adapters and for quality filtering, discarding sequence reads (1) with more than one ‘N’ (maxns = 1), (2) shorter than 50 bp after trimming (minlength = 50) and (3) with average quality <10 after trimming (maq = 10). FastQC and MultiQC were used for second round of quality check for the clean reads. Sequencing coverage and mapping rate was calculated for each sample (Supplementary Data Tables 1 and 2).

### Identifying core and variable genes

To analyse genetic loci present throughout the global distribution range of eelgrass, we focused on identifying core genes that are present in genomes of all individuals. To do so, each of the 190 ramets were de novo assembled using HipMer (*k* = 51) (ref. ^59^). To categorize, extract and compare core and variable (shell and cloud) genes, primary transcript sequences (21,483 gene models) from the *Z. marina* reference (V3.1; ref. ^19^) were aligned using BLAT using default parameters^60^ to each de novo assembly. Genes were considered present if the transcript aligned with either (1) >60% identity and >60% coverage from a single alignment or (2) >85% identity and >85% coverage split across three or fewer scaffolds. Individual presence–absence-variation calls were combined into a matrix to classify genes into core, cloud and shell categories based on their observation across the population. The total number of genes considered was 20,100. Because identical genotypes and fragmented, low-quality assemblies can bias and skew presence–absence-variation analyses, only 141 single representatives of clones and ramets with greater than 17,500 genes were kept to ensure that only unique, high-quality assemblies were retained. Genes were classified using discriminant analysis of principal components^61^ into cloud, shell and core gene clusters based on their frequency. Core genes were the largest category, with 18,717 genes that were on average observed in 97% of ramets.

### SNP mapping, calling and filtering

The quality-filtered reads were mapped against the chromosome-level *Z. marina* reference genome V3.1 using BWA MEM^62^. The alignments were converted to BAM format and sorted using Samtools^62^. The MarkDuplicates module in GATK4 (ref. ^63^) was used to identify and tag duplicate reads in the BAM files. The mapping rate for each genotype was calculated using Samtools (Supplementary Data Table 2). HaplotypeCaller (GATK4) was used to generate a Genomic Variant Call Format (GVCF) file for each sample, and all the GVCF files were combined by CombineGVCFs (GATK4). GenotypeGVCFs (GATK4) was used to call genetic variants.

BCFtools^64^ was used to remove SNPs within 20 base pairs of an indel or other variant type (Supplementary Fig. 1), as these variant types may cause erroneous SNPs calls. VariantsToTable (GATK4) was used to extract INFO annotations. SNPs meeting one or more than one of the following criteria were marked by VariantFiltration (GATK4): MQ < 40.0; FS > 60.0; QD < 10.0; MQRandSum > 2.5 or MQRandSum < −2.5; ReadPosRandSum < −2.5; ReadPosRandSum > 2.5; SOR > 3.0; DP > 10,804.0 (2 × average DP). Those SNPs were excluded by SelectVariants (GATK4). A total of 3,975,407 SNPs were retained. VCFtools^65^ was used to convert individual genotypes to missing data when GQ < 30 or DP < 10. Individual homozygous reference calls with one or more reads supporting the variant allele, and individual homozygous variant calls with ≥1 read supporting the reference, were set as missing data. Only bi-allelic SNPs were kept (3,892,668 SNPs). To avoid the reference-genome-related biases, due to the large Pacific–Atlantic genomic divergence, we focused on the 18,717 core genes that were on average observed in 97% of ramets. Bedtools^66^ was used to find overlap between the SNPs and the core genes, and only those SNPs were kept (ZM_HQ_SNPs, 763,580 SNPs). Genotypes that were outside our custom quality criteria were represented as missing data.

### Excluding clone mates and genotypes originating from selfing

Based on the extended data set ZM_HQ_SNPs (763,580 SNPs; Supplementary Fig. 1), possible parent–descendant pairs under selfing (Supplementary Table 2) as well as clonemates were detected based on the shared heterozygosity (ref. ^67^). To ensure that all genotypes assessed originated by random mating, ten ramets showing evidence for selfing were excluded. Seventeen multiple sampled clonemates were also excluded (Supplementary Table 3 and Supplementary Fig. 3). Based on ZM_HQ_SNPs (763,580 SNPs), we calculated the sample-wise missing rate using a custom Python3 script and plotted results as a histogram (Supplementary Fig. 4). Missing rates were mostly <15%, except for ten ramets (ALI01, ALI02, ALI03, ALI04, ALI05, ALI06, ALI10, ALI16, QU03 and SD08) that were also excluded. After the exclusion of these 37 samples owing to missing data, selfing or clonality, 153 samples were left for further analyses.

### Chloroplast haplotypes

The chloroplast genome was de novo assembled by NOVOPlasty^68^. The chloroplast genome of *Z. marina* was represented by a circular molecule of 143,968 bp with a classic quadripartite structure: two identical inverted repeats (IRa and IRb) of 24,127 bp each, a large single-copy region of 83,312 bp, and a small single-copy region of 12,402 bp. All regions were equally taken into SNP calling analysis except for 9,818 bp encoding 23S and 16S ribosomal RNAs due to bacterial contamination in some samples. The raw Illumina reads of each individual were aligned by BWA MEM to the assembled chloroplast genome. The alignments were converted to BAM format and then sorted using Samtools^62^. Genomic sites were called as variable positions when the frequency of variant reads was >50% (Supplementary Fig. 8) and the total coverage of the position was >30% of the median coverage (174 variable positions). Then 11 positions likely related to microsatellites and 12 positions reflecting minute inversions caused by hairpin structures^69^ were removed from the final set of variable positions for the haplotype reconstruction (151 SNPs). For the phylogenetic tree reconstruction, we further selected 108 SNPs that represent parsimony-informative sites (that is, no singletons).

### Putatively neutral and non-linked SNPs

Among the 153 unique samples that were retained for analyses, SnpEff (http://pcingola.github.io/SnpEff/) was used to annotate each SNP as genic or non-genic, and within the former category as synonymous or non-synonymous. To obtain putatively neutral SNPs, we kept only SNPs annotated as ‘synonymous_variant’ (ZM_Neutral_SNPs, 144,773 SNPs). For the SNPs in ZM_Neutral_SNPs (144,773 SNPs), only SNPs without any missing data were kept, which resulted in 44,865 SNPs, the data set used for calculating *π* (Supplementary Figs. 1 and 2). To obtain putatively non-linked SNP loci for the coalescence runs, we thinned sites using VCFtools to achieve a minimum pairwise distance (physical distance in the reference genome) of 3,000 bp to obtain our core data set, hereafter ZM_Core_SNPs, corresponding to 11,705 SNPs.

### Population structure based on nuclear and chloroplast polymorphism

We used R packages to run a global PCA based on ZM_HQ_SNPs, (=763,580 SNPs). The package vcfR^70^ was used to load the VCF format file, and function glPca in adegenet package to conduct PCA analyses, followed by visualization through the ggplot2 package.

We used Bayesian clustering implemented in STRUCTURE to study population structure and potential admixture^22^. To reduce the run time, we randomly selected 2,353 SNPs from ZM_Core_SNPs (20%) to run STRUCTURE (length of burn-in period 3 × 10^5^; number of Markov chain Monte Carlo runs 2 × 10^6^). Ten runs were performed for *K* values 1–10. StructureSelector^25^ was used to help determine the optimal number of clusters (*K*) based on the original Delta-*K* method^23^ in conjunction with additional metrics proposed by ref. ^24^ that give an upper limit to the number of clusters. We considered the hierarchical structure of our data set owing to the marked Pacific–Atlantic divide and always performed a qualitative inspection of alternative major and minor *K* modes.

To detect hidden hierarchical population structure, we further analysed populations from the Atlantic and Pacific alone. Pacific data were extracted from ZM_Neutral_SNPs (144,773 SNPs), excluding monomorphic sites and those with missing data. To obtain putatively independent SNPs, we thinned sites using VCFtools, so that no two sites were within 3,000 bp distance (physical distance in the reference genome) from one another (ZM_Pacific_SNPs, 12,514 SNPs). Those 12,514 SNPs were subjected to PCA, while a set of randomly selected 6,168 SNPs was used in STRUCTURE to reduce run times (length of burn-in period 3 × 10^5^; number of Markov chain Monte Carlo runs, 2 × 10^6^) as described above, with possible *K* values 1–7.

Polymorphism data for Atlantic and Mediterranean eelgrass were also extracted from ZM_Neutral_SNPs (144,773 SNPs). To obtain putatively independent SNPs, we thinned sites using VCFtools according to the above criteria. The resulting 8,552 SNPs were then used to run another separate PCA and STRUCTURE using the parameters above. For STRUCTURE analysis, *K* was set from 1 to 5. For each *K*, we repeated the analysis 10 times independently (Supplementary Figs. 6 and 7).

The population structure of cpDNA was explored using a haplotype network, constructed via the Median Joining Network method^71^ with epsilon 0 and 1 implemented by PopART^72^, based on 151 polymorphic sites. The topology was additionally confirmed using a maximum-likelihood phylogenetic tree, reconstructed by IQ-TREE v1.5.5 with 1,000 bootstrap replicates^32^ based on 108 parsimony-informative polymorphic sites (Extended Data Fig. 1).

### Analysis of reticulate evolution using split network

To assess reticulate evolutionary processes, we used SplitsTree4^26^, a combinatorial generalization of phylogenetic trees designed to represent incompatibilities. A custom Python3 script was used to generate a fasta format file containing concatenated DNA sequences for all ramets based on ZM_Core_SNPs. As the majority of genotypes were heterozygous, one allele had to be randomly selected to represent the site for an individual. We checked for consistency by re-rerunning the analysis with different randomly selected SNP sets and found identical topologies and similar split weights. The fasta format file was converted to nexus format file using MEGAX^73^, which was fed to SplitsTree4. NeighborNet method was used to construct the split network.

### Genetic diversity

VCFtools was used to calculate nucleotide diversity (*π*) for each population at all synonymous sites using each of the six chromosomes as replicates for 44,685 SNPs without any missing data (Supplementary Fig. 1). Genomic heterozygosity for a given genotype *H*_OBS_ (as (number of heterozygous sites)/(total number of sites with available genotype calls)) was calculated using a custom Python3 script based on all synonymous SNPs (144,773).

### Pairwise population differentiation using *F*_ST_

We used the function stamppFst in the StAMPP-R package^74^ to calculate pairwise *F*_ST_ based on ZM_Core_SNPs (Supplementary Table 5). *P* values were generated by 1,000 bootstraps across loci.

### *D*-statistics

Patterson’s *D* provides a simple and powerful test for the deviation from a bifurcating evolutionary history. The test is applied to three populations, P1, P2 and P3 plus an outgroup O, with P1 and P2 being sister populations. If P3 shares more derived alleles with P2 than with P1, Patterson’s *D* will be positive. We used Dsuite^28^ to calculate *D* values for populations within the Pacific and within the Atlantic Oceans (Extended Data Fig. 2). *D* was calculated for trios of *Z. marina* populations based on the SNP core data set (ZMZJ_D_SNPs) (Supplementary Fig. 2), using *Z. japonica* as outgroup. The Ruby script plot_d.rb (https://github.com/mmatschiner/tutorials/blob/master/analysis_of_introgression_with_snp_data/src/plot_d.rb) was used to plot a heat map that jointly visualizes both the *D* value and the associated *P* value for each comparison of P2 and P3. The colour of the corresponding heat map cell indicates the most significant *D* value across all possible populations in position P1. Red colours indicate higher *D* values, and more saturated colours indicate greater significance.

### Phylogenetic tree with estimated divergence time

To estimate the divergence time among major groups, we used the MSC in combination with a strict molecular clock model^11^. We used the software SNAPP^9^ with an input file prepared by script ‘snapp_prep.rb’ (github.com/mmatschiner/snapp_prep). Two specimens were randomly selected from each of the included populations, and genotype information was extracted from ZMZJ_Neutral_SNPs (Supplementary Figs. 1 and 2). Monomorphic sites were excluded. Only SNPs without any missing data were kept. To obtain putatively independent SNPs, we thinned sites using VCFtools so that no two sites included SNPs that were within 3,000 bp (physical distance in the reference genome) from one another (6,169 SNPs). The estimated divergence time between *Z. japonica* and *Z. marina* was used as a calibration point, which was implemented as a lognormal prior distribution (Supplementary Note 4, mean = 11.154 mya, s.d. = 0.07).

Most of the 6,169 SNPs above represented the genetic differences between *Z. japonica* and *Z. marina* and were monomorphic in *Z. marina*. To obtain a better estimation among *Z. marina* populations, we performed a second, *Z. marina*-specific SNAPP analysis via subsampling from the ZM_Neutral_SNPs (144,773 SNPs) data set, excluding monomorphic sites and missing data. We thinned sites again using VCFtools, so that all sites were ≥3,000 bp distance from one another (13,732 SNPs). The crown divergence for all *Z. marina* populations, estimated in the first SNAPP analysis, was used as calibration point, assuming a lognormal prior distribution (mean = 0.3564 mya, s.d. = 0.1).

As the MSC model does not account for genetic exchange, the SNAPP analysis was repeated after removing populations showing admixture in STRUCTURE (Fig. 2), SplitsTree (Fig. 3) and *D*-statistics (Extended Data Fig. 2). We hence reduced the data set by excluding JN (admixed with Alaska), as well as WAS and BB (involved in admixture with SD). We also explored how this exclusion of admixed populations progressively affected the SNAPP phylogenetic tree topology (Supplementary Fig. 11b–d). As alternative coalescent method, an ASTRAL analysis based on 617 core genes in combination with divergence time estimation using StarBEAST2 was conducted (Supplementary Note 2). Incomplete lineage sorting was examined using ASTRAL quartet analysis^30^ (Supplementary Fig. 11), and the alternative dating of divergence events is presented in Supplementary Fig. 12.

### Demographic analysis

The MSMC^33^ was run for each genotype per population. We focused on time intervals where different replicate runs per population converged, because MSMC creates unreliable estimates in recent time^34^. Owing to differences in the relative amount of sexual versus clonal or vegetative reproduction, the generation time of *Z. marina* varies across populations. We therefore refrained from representing the *x* axis in absolute time. We first generated one mappability mask file for each of the six main chromosomes using SNPable (http://lh3lh3.users.sourceforge.net/snpable.shtml). Only chromosomal regions that permitted unique mapping of sequencing reads were considered. We generated one mask file for all core genes along each of the six main chromosomes. We generated one ramet-specific mask file based on the BAM format file using bamCaller.py (https://github.com/stschiff/msmc-tools), containing the chromosomal regions with sufficient coverage of any genoytpe, with minDepth = 10. We also generated a ramet-specific VCF file for each of the six main chromosomes based on ZM_HQ_SNPs using a custom Python3 script.

### Recolonization scenarios after the LGM for the Atlantic

Simulations using DIYABC-RF^75^ were run to distinguish between alternative models of the recolonization history of *Z. marina* after the LGM. Considering that the Mediterranean Sea had its own glacial refugium, the ABC modelling was conducted for the Atlantic only. We constructed three recolonization scenarios (Supplementary Fig. 10): (1) NC and MA were glacial refugia in the Atlantic, which first recolonized QU as a stepping stone and then the Northeast Atlantic. (2) NC and MA represent the only glacial refugia in the Atlantic. Both QU and Northeast Atlantic were directly recolonized by the glacial refugia. (3) NC and MA represent the southern glacial refugia for the Northwest Atlantic only. Note that this analysis cannot cover any additional East Atlantic refugia that were not sampled (Supplementary Note 7).

### Reporting summary

Further information on research design is available in the Nature Portfolio Reporting Summary linked to this article.

## Supplementary information

Supplementary Information Supplementary Notes 1–8, Tables 1–6 and Figs. 1–12.

Reporting Summary

Supplementary Data Supplementary Data Table 1: Sequence coverage. Supplementary Data Table 2: Mapping rate. Supplementary Data Table 3: Accession number of each library.

## Data Availability

Genome data have been deposited in Genbank (short read archive, Supplementary Data Table 3). Coding sequences of *Z. japonica* and *Z*. *marina* for the ASTRAL analysis can be found on figshare (10.6084/m9.figshare.21626327.v1). VCF files of the 11,705 core SNPs can be accessed at 10.6084/m9.figshare.21629471.v1. Source data for Fig. 1b,c are given, as well as statistics of sequencing coverage, mapping rate and further specifications of each sequenced library (Supplementary Tables 1–3). Source data are provided with this paper.

## Source data

Source Data Fig. 1b,c Raw data on nucleotide diversity (1b) and population-wise heterozygosity (1c) for each sample in all 16 populations.

## Code availability

Custom-made scripts are deposited on GitHub for SNP filtering (github.com/leiyu37/populationGenomics_ZM.git), for clone mate detection (github.com/leiyu37/Detecting-clonemates.git), for heterozygote and nucleotide diversity quantification (github.com/leiyu37/populationGenomics_ZM.git) and to prepare SplitsTree input files (https://github.com/leiyu37/populationGenomics_ZM/blob/main/10_SplitsTree/vcf2alignment.py) and SNAPP input files (github.com/mmatschiner/snapp_prep). Scripts for calculating *D*-statistics are available at github.com/mmatschiner/tutorials/blob/master/analysis_of_introgression_with_snp_data/src/plot_d.rb. Scripts to prepare the gene presence/absence analysis are deposited on https://github.com/leiyu37/populationGenomics_ZM/tree/main/gene_presense_absence_analysis. Further software code for the MSMC analysis are found at http://lh3lh3.users.sourceforge.net/snpable.shtml (generation of mappability mask file for each of six chromosomes using SNPable) and at https://github.com/stschiff/msmc-tools (generation of ramet-specific mask file based on a bam file using bamCaller.py).

## References

[1] Chen L-Y (2022). Phylogenomic analyses of Alismatales shed light into adaptations to aquatic environments. Mol. Biol. Evol..

[2] Unsworth RKF, Cullen-Unsworth LC, Jones BLH, Lilley RJ (2022). The planetary role of seagrass conservation. Science.

[3] Green, E. P. & Short, F. T. *World Atlas of Seagrasses* (Univ. California Press, 2003).

[4] Röhr ME (2018). Blue carbon storage capacity of temperate eelgrass (*Zostera marina*) meadows. Glob. Biogeochem. Cycles.

[5] Coyer JA (2013). Phylogeny and temporal divergence of the seagrass family Zosteraceae using one nuclear and three chloroplast loci. Syst. Biodivers..

[6] Waycott, M., Biffin, E. & Les, D. H. in *Seagrasses of Australia: Structure, Ecology and Conservation* (eds Larkum, A. W. D., Kendrick, G. A. & Ralph, P. J.) 129–154 (Springer International, 2018).

[7] Harwell MC, Orth RJ (2002). Long-distance dispersal potential in a marine macrophyte. Ecology.

[8] Marske KA, Rahbek C, Nogués-Bravo D (2013). Phylogeography: spanning the ecology–evolution continuum. Ecography.

[9] Bryant D, Bouckaert R, Felsenstein J, Rosenberg NA, RoyChoudhury A (2012). Inferring species trees directly from biallelic genetic markers: bypassing gene trees in a full coalescent analysis. Mol. Biol. Evol..

[10] Zhang C, Rabiee M, Sayyari E, Mirarab S (2018). ASTRAL-III: polynomial time species tree reconstruction from partially resolved gene trees. BMC Bioinform..

[11] Stange M, Sánchez-Villagra MR, Salzburger W, Matschiner M (2018). Bayesian divergence-time estimation with genome-wide single-nucleotide polymorphism data of sea catfishes (Ariidae) supports Miocene closure of the Panamanian Isthmus. Syst. Biol..

[12] Hewitt G (2000). The genetic legacy of the Quaternary ice ages. Nature.

[13] Bringloe TT, Verbruggen H, Saunders GW (2020). Unique biodiversity in Arctic marine forests is shaped by diverse recolonization pathways and far northern glacial refugia. Proc. Natl Acad. Sci. USA.

[14] Neiva J (2018). Glacial vicariance drives phylogeographic diversification in the amphi-boreal kelp *Saccharina latissima*. Sci. Rep..

[15] Marko PB (2010). The ‘expansion–contraction’ model of Pleistocene biogeography: rocky shores suffer a sea change?. Mol. Ecol..

[16] Hewitt, G. M. & Nichols, R. A. in *Climate Change and Biodiversity* (eds Lovejoy, T. E. & Hannah. L.) 176–192 (Yale Univ. Press, 2005).

[17] Duffy JE (2022). A Pleistocene legacy structures variation in modern seagrass ecosystems. Proc. Natl Acad. Sci. USA.

[18] Clark PU (2009). The Last Glacial Maximum. Science.

[19] Ma X (2021). Improved chromosome-level genome assembly and annotation of the seagrass, *Zostera marina* (eelgrass). F1000Research.

[20] Danilevicz MF, Tay Fernandez CG, Marsh JI, Bayer PE, Edwards D (2020). Plant pangenomics: approaches, applications and advancements. Curr. Opin. Plant Biol..

[21] Olsen JL (2016). The genome of the seagrass *Zostera marina* reveals angiosperm adaptation to the sea. Nature.

[22] Pritchard JK, Stephens M, Donnelly P (2000). Inference of population structure using multilocus genotype data. Genetics.

[23] Evanno G, Regnaut S, Goudet J (2005). Detecting the number of clusters of individuals using the software STRUCTURE: a simulation study. Mol. Ecol..

[24] Puechmaille SJ (2016). The program structure does not reliably recover the correct population structure when sampling is uneven: subsampling and new estimators alleviate the problem. Mol. Ecol. Resour..

[25] Li Y-L, Liu J-X (2018). StructureSelector: a web-based software to select and visualize the optimal number of clusters using multiple methods. Mol. Ecol. Resour..

[26] Huson DH, Bryant D (2006). Application of phylogenetic networks in evolutionary studies. Mol. Biol. Evol..

[27] Patterson N (2012). Ancient admixture in human history. Genetics.

[28] Malinsky M, Matschiner M, Svardal H (2021). Dsuite—fast D-statistics and related admixture evidence from VCF files. Mol. Ecol. Resour..

[29] Marincovich L, Gladenkov AY (1999). Evidence for an early opening of the Bering Strait. Nature.

[30] Zhang C, Scornavacca C, Molloy EK, Mirarab S (2020). ASTRAL-Pro: quartet-based species-tree inference despite paralogy. Mol. Biol. Evol..

[31] Ogilvie HA, Bouckaert RR, Drummond AJ (2017). StarBEAST2 brings faster species tree inference and accurate estimates of substitution rates. Mol. Biol. Evol..

[32] Nguyen L-T, Schmidt HA, von Haeseler A, Minh BQ (2015). IQ-TREE: a fast and effective stochastic algorithm for estimating maximum-likelihood phylogenies. Mol. Biol. Evol..

[33] Schiffels S, Durbin R (2014). Inferring human population size and separation history from multiple genome sequences. Nat. Genet..

[34] Schiffels, S. & Wang, K. in *Statistical Population Genomics* pp. 147-166 (Humana, 2020).

[35] Cortés AJ, López-Hernández F, Osorio-Rodriguez D (2020). Predicting thermal adaptation by looking into populations’ genomic past. Front. Genet..

[36] Gross CP (2022). The biogeography of community assembly: latitude and predation drive variation in community trait distribution in a guild of epifaunal crustaceans. Proc. R. Soc. B.

[37] Gallagher SJ (2015). The Pliocene to recent history of the Kuroshio and Tsushima Currents: a multi-proxy approach. Prog. Earth Planet. Sci..

[38] Burton RS (1998). Intraspecific phylogeography across the Point Conception biogeographic boundary. Evolution.

[39] Checkley DM, Barth JA (2009). Patterns and processes in the California Current System. Prog. Oceanogr..

[40] Talbot SL (2016). The structure of genetic diversity in eelgrass (*Zostera marina* L.) along the North Pacific and Bering Sea coasts of Alaska. PLoS ONE.

[41] Laakkonen HM, Hardman M, Strelkov P, Väinölä R (2021). Cycles of trans-Arctic dispersal and vicariance, and diversification of the amphi-boreal marine fauna. J. Evol. Biol..

[42] Coyer JA, Hoarau G, Van Schaik J, Luijckx P, Olsen JL (2011). Trans-Pacific and trans-Arctic pathways of the intertidal macroalga *Fucus distichus* L. reveal multiple glacial refugia and colonizations from the North Pacific to the North Atlantic. J. Biogeogr..

[43] Maggs CA (2008). Evaluating signals of glacial refugia for North Atlantic benthic taxa. Ecology.

[44] Jenkins T, Castilho R, Stevens J (2018). Meta-analysis of northeast Atlantic marine taxa shows contrasting phylogeographic patterns following post-LGM expansions. PeerJ.

[45] Li, J.-J., Hu, Z.-M. & Duan, D.-L. in *Seaweed Phylogeography: Adaptation and Evolution of Seaweeds Under Environmental Change* (eds Hu, Z.-M. & Fraser, C.) 309–330 (Springer, 2016).

[46] Olsen JL (2004). North Atlantic phylogeography and large-scale population differentiation of the seagrass *Zostera marina* L. Mol. Ecol..

[47] Larkum, A. W. D., Orth, R. J. & Duarte, C. M. *Seagrasses: Biology, Ecology and Conservation* (Springer, 2006).

[48] Palumbi SR (1994). Genetic divergence, reproductive isolation, and marine speciation. Annu. Rev. Ecol. Syst..

[49] Franssen SU (2011). Transcriptomic resilience to global warming in the seagrass *Zostera marina*, a marine foundation species. Proc. Natl. Acad. Sci. USA.

[50] Bertelli CM, Unsworth RKF (2014). Protecting the hand that feeds us: seagrass (*Zostera marina*) serves as commercial juvenile fish habitat. Mar. Pollut. Bull..

[51] Reusch TBH (2021). Lower *Vibrio* spp. abundances in *Zostera marina* leaf canopies suggest a novel ecosystem function for temperate seagrass beds. Mar. Biol..

[52] Macreadie PI (2021). Blue carbon as a natural climate solution. Nat. Rev. Earth Environ..

[53] Stevenson A, Corcora TCÓ, Hukriede W, Schubert P, Reusch TBH (2022). Substantial seagrass blue carbon pools in the southwestern Baltic Sea are spatially heterogeneous, mostly autochthonous, and include historically terrestrial peatlands. Front. Mar. Sci..

[54] Hämmerli A, Reusch TBH (2003). Flexible mating: experimentally induced sex-ratio shift in a marine clonal plant. J. Evol. Biol..

[55] Reusch TBH (2000). Pollination in the marine realm: microsatellites reveal high outcrossing rates and multiple paternity in eelgrass *Zostera marina*. Heredity.

[56] Yu L (2020). Somatic genetic drift and multilevel selection in a clonal seagrass. Nat. Ecol. Evol..

[57] Reusch TBH, Boström C, Stam WT, Olsen JL (1999). An ancient eelgrass clone in the Baltic Sea. Mar. Ecol. Prog. Ser..

[58] Ewels P, Magnusson M, Lundin S, Käller M (2016). MultiQC: summarize analysis results for multiple tools and samples in a single report. Bioinformatics.

[59] Georganas, E. et al. In *SC ‘15*: *Proc. International Conference for High Performance Computing*, *Networking, Storage and Analysis* pp. 1–11 (2015).

[60] Kent WJ (2002). BLAT—the BLAST-like alignment tool. Genome Res..

[61] Jombart T (2008). adegenet: a R package for the multivariate analysis of genetic markers. Bioinformatics.

[62] Li H, Durbin R (2009). Fast and accurate short read alignment with Burrows–Wheeler transform. Bioinformatics.

[63] Van der Auwera, G. A. & O’Connor, B. D. *Genomics in the Cloud: Using Docker, GATK, and WDL in Terra* (O’Reilly Media, 2020).

[64] Li H (2011). A statistical framework for SNP calling, mutation discovery, association mapping and population genetical parameter estimation from sequencing data. Bioinformatics.

[65] Danecek P (2011). The variant call format and VCFtools. Bioinformatics.

[66] Quinlan AR, Hall IM (2010). BEDTools: a flexible suite of utilities for comparing genomic features. Bioinformatics.

[67] Yu L, Stachowicz JJ, DuBois K, Reusch TBH (2023). Detecting clonemate pairs in multicellular diploid clonal species based on a shared heterozygosity index. Mol. Ecol. Resour..

[68] Dierckxsens N, Mardulyn P, Smits G (2017). NOVOPlasty: de novo assembly of organelle genomes from whole genome data. Nucleic Acids Res..

[69] Petit, R. J. & Vendramin, G. G. in *Phylogeography of Southern European Refugia: Evolutionary Perspectives on the Origins and Conservation of European Biodiversity* (eds Weiss, S. & Ferrand, N.) 23–97 (Springer, 2007).

[70] Knaus BJ, Grünwald NJ (2017). vcfr: a package to manipulate and visualize variant call format data in R. Mol. Ecol. Resour..

[71] Bandelt HJ, Forster P, Röhl A (1999). Median-joining networks for inferring intraspecific phylogenies. Mol. Biol. Evol..

[72] Leigh JW, Bryant D (2015). popart: full-feature software for haplotype network construction. Methods Ecol. Evol..

[73] Kumar S, Stecher G, Li M, Knyaz C, Tamura K (2018). MEGA X: molecular evolutionary genetics analysis across computing platforms. Mol. Biol. Evol..

[74] Pembleton LW, Cogan NOI, Forster JW (2013). StAMPP: an R package for calculation of genetic differentiation and structure of mixed-ploidy level populations. Mol. Ecol. Resour..

[75] Collin F-D (2021). Extending approximate Bayesian computation with supervised machine learning to infer demographic history from genetic polymorphisms using DIYABC Random Forest. Mol. Ecol. Resour..

[76] Murphy GEP (2021). From coast to coast to coast: ecology and management of seagrass ecosystems across Canada. FACETS.

[77] Jahnke M (2018). Seascape genetics and biophysical connectivity modelling support conservation of the seagrass *Zostera marina* in the Skagerrak–Kattegat region of the eastern North Sea. Evol. Appl..

[78] Letunic I, Bork P (2021). Interactive Tree Of Life (iTOL) v5: an online tool for phylogenetic tree display and annotation. Nucleic Acids Res..

