# Author Correction: Ocean current patterns drive the worldwide colonization of eelgrass (*Zostera marina*)

**DOI:** 10.1038/s41477-023-01504-y

**Published:** 2023-08-07

**Authors:** Lei Yu, Marina Khachaturyan, Michael Matschiner, Adam Healey, Diane Bauer, Brenda Cameron, Mathieu Cusson, J. Emmett Duffy, F. Joel Fodrie, Diana Gill, Jane Grimwood, Masakazu Hori, Kevin Hovel, A. Randall Hughes, Marlene Jahnke, Jerry Jenkins, Keykhosrow Keymanesh, Claudia Kruschel, Sujan Mamidi, Damian M. Menning, Per-Olav Moksnes, Masahiro Nakaoka, Christa Pennacchio, Katrin Reiss, Francesca Rossi, Jennifer L. Ruesink, Stewart T. Schultz, Sandra Talbot, Richard Unsworth, David H. Ward, Tal Dagan, Jeremy Schmutz, Jonathan A. Eisen, John J. Stachowicz, Yves Van de Peer, Jeanine L. Olsen, Thorsten B. H. Reusch

**Affiliations:** 1 Marine Evolutionary Ecology, GEOMAR Helmholtz Centre for Ocean Research Kiel, Kiel, Germany; 2 Institute of General Microbiology, Kiel University, Kiel, Germany; 3 Department of Paleontology and Museum, University of Zurich, Zurich, Switzerland; 4 Natural History Museum, University of Oslo, Oslo, Norway; 5 Genome Sequencing Center, HudsonAlpha Institute for Biotechnology, Huntsville, AL USA; 6 US Department of Energy Joint Genome Institute, Lawrence Berkeley National Laboratory, Berkeley, CA USA; 7 Department of Evolution and Ecology, University of California, Davis, CA USA; 8 Département des sciences fondamentales, Université du Québec à Chicoutimi, Chicoutimi, Quebec Canada; 9 Tennenbaum Marine Observatories Network, Smithsonian Environmental Research Center, Edgewater, MD USA; 10 Institute of Marine Sciences (UNC-CH), Morehead City, NC USA; 11 Japan Fisheries Research and Education Agency, Yokohama, Japan; 12 Department of Biology, San Diego State University, San Diego, CA USA; 13 Marine Science Center, Northeastern University, Nahant, MA USA; 14 Tjärnö Marine Laboratory, Department of Marine Sciences, University of Gothenburg, Strömstad, Sweden; 15 University of Zadar, Zadar, Croatia; 16 US Geological Survey, Alaska Science Center, Anchorage, AK USA; 17 Department of Marine Sciences, University of Gothenburg, Gothenburg, Sweden; 18 Hokkaido University, Akkeshi, Japan; 19 Nord University, Bodø, Norway; 20 Department of Integrative Marine Ecology (EMI), Stazione Zoologica Anton Dohrn–National Institute of Marine Biology, Ecology and Biotechnology, Genoa, Italy; 21 Department of Biology, University of Washington, Seattle, WA USA; 22 Far Northwestern Institute of Art and Science, Anchorage, AK USA; 23 Department of Biosciences, Swansea University, Swansea, UK; 24 Project Seagrass, the Yard, Bridgend, UK; 25 Center for Population Biology, University of California, Davis, CA USA; 26 Department of Plant Biotechnology and Bioinformatics, Ghent University, Gent, Belgium; 27 Center for Microbial Ecology and Genomics, Department of Biochemistry, Genetics and Microbiology, University of Pretoria, Pretoria, South Africa; 28 College of Horticulture, Academy for Advanced Interdisciplinary Studies, Nanjing Agricultural University, Nanjing, China; 29 VIB-UGent Center for Plant Systems Biology, Gent, Belgium; 30 Groningen Institute for Evolutionary Life Sciences, Groningen, The Netherlands

**Keywords:** Population genetics, Plant evolution, Marine biology

Correction to: *Nature Plants* https://www.nature.com/articles/s41477-023-01464-3. Published online 20 July 2023.

In the version of the article initially published, Yves Van de Peer’s name appeared incorrectly as Yves Van De Peer. In addition, two of Yves Van de Peer’s affiliations were wrongly combined into a single affiliation. These have been separated, and now read: Department of Plant Biotechnology and Bioinformatics, Ghent University, Gent, Belgium and VIB-UGent Center for Plant Systems Biology, Gent, Belgium. These errors have been corrected in the HTML and PDF versions of the article.

