# Supplementary material for: Ocean current patterns drive the worldwide colonization of eelgrass (Zostera marina)
Source: Nat Plants. 2023 Jul 20;9(8):1207–20. doi: 10.1038/s41477-023-01464-3 (PMC10435387; doi:10.1038/s41477-023-01464-3)
Supplement: Supplementary file 1 — Supplementary Notes 1–8, Tables 1–6 and Figs. 1–12. [file 41477_2023_1464_MOESM1_ESM.pdf]

nature plants

Article

https://doi.org/10.1038/s41477-023-01464-3

Ocean current patterns drive the worldwide colonization of eelgrass (*Zostera marina*)

In the format provided by the authors and unedited

Table of Contents

Supplementary Note 1 - Ecological niches, distribution and dispersal ecology of Zostera marina *3*

Ecological niches of temperate-to-Arctic North Atlantic seagrass species 3

Temperate-to-Arctic North Pacific seagrass species 4

Dispersal ecology of *Zostera marina* 4

Supplementary Note 2 - Divergence time estimation using ASTRAL *5*

Constructing an ASTRAL species tree 5

ASTRAL normalized quartet scores 5

Divergence time estimates using StarBEAST2 6

Supplementary Note 3 - Geographic origin of the Zosteraceae and of Zostera marina *7*

Supplementary Note 4 - Rate estimation of the synonymous molecular clock in Zostera marina *8*

Estimating the divergence time between *Zostera japonica* and *Z. marina* 8

Estimating the mutation rate 9

SNP calling and filtering including *Z. japonica* 9

Supplementary Note 5 - The Role of the North Pacific Current (NPC) gateway for eelgrass population admixture *10*

Supplementary Note 6 - Using the cpDNA haplotype clock as an alternative dating estimate *13*

Estimating chloroplast haplotype divergence time 13

Supplementary Note 7 - Eelgrass refugia during the last glacial maximum in the Atlantic area *14*

Marine refugia during the last LGM 14

Eelgrass refugia in the Atlantic 14

Supplementary Note 8 - Zostera marina and Z. pacifica in the San Diego to Baja region *17*

Supplementary Tables *18*

Supplementary Table 1: Population metadata for 16 worldwide *Zostera marina* populations 18

Supplementary Table 2: Possible parent-descendant pairs under self-fertilization as detected using the shared heterozygosity index. 19

Supplementary Table 3: Clone mates detected based on the shared heterozygosity index. 19

Supplementary Table 4: Statistical comparison of genetic diversity measures 20

Supplementary Table 5: Matrix of F_{ST}-values among population pairs 21

Supplementary Table 6: *Zostera marina*-associated fauna among Pacific and Atlantic locations 22

Supplementary Figures *25*
Supplementary Fig. 1: Workflow for SNP calling and filtering. 25
Supplementary Fig. 2: Workflow for SNP calling and filtering that required an outgroup. 26
Supplementary Fig. 3: Detecting clone mates and possible parent-descendant pairs under selfing based on shared heterozygosity (SH) 27
Supplementary Fig. 4: Missing data distribution for each sample. 28
Supplementary Fig. 5: Global STRUCTURE results for K from 1 to 10. 29
Supplementary Fig. 6: STRUCTURE results for Pacific populations only for K from 1 to 7. 30
Supplementary Fig. 7: STRUCTURE results for Atlantic populations for K from 1 to 5. 31
Supplementary Fig. 8: Distribution of variant allele frequencies based on 163 chloroplast genomes 32
Supplementary Fig. 9: Alternative SNAPP topologies when admixed populations are included. 34
Supplementary Fig. 10: Most likely recolonization scenario of the Atlantic Ocean after the Last Glacial Maximum (LGM) using approximate Bayesian computation (ABC). 35
Supplementary Fig. 11: Unrooted phylogenetic tree using ASTRAL 36
Supplementary Fig. 12: StarBEAST2 phylogenetic tree including divergence time estimation.... 37

Supplementary Note 1 - Ecological niches, distribution and dispersal ecology of *Zostera marina*

Ecological niches of temperate-to-Arctic North Atlantic seagrass species

Zostera marina has a very broad thermal range from -1.5 to 30°C and spans nearly 50° latitude extending as far north as 72°N; Lee et al. 2007). Although mixed beds occur, *Z. marina* is typically monospecific, thus creating a unique foundational niche for which there is no substitute.

On the northwestern Atlantic coast, the southern distribution limit of eelgrass is in North Carolina (~34°N), where it sometimes co-occurs with the subtropical species *Halodule wrightii*. The latter species reaches its northern distribution limit in North Carolina (Ferguson et al. 1993). *Ruppia maritima* also occurs in mixed beds with *Z. marina* in parts of inner Chesapeake Bay (Koch & Orth 2003). All three species are found in lagoon settings and not along exposed shores. They colonize a wide range of salinities (from 10-20 psu= practical salinity units to fully marine at 30-35 psu).

On the northeastern Atlantic coast, eelgrass occurs as far north as southern Greenland (~64°N) and Northern Norway (~70°N), and as far south as southern Portugal (~37°N) where it sometimes intermingles with the subtropical species *Cymodosa nodosa. C. nodosa* occurs only as far north in Portugal as 42°N (Chefaoui et al. 2016) but is also widely distributed throughout the Mediterranean, sometimes mixed with patches of *Z. marina*. It cannot survive in winter temperatures <6°C. *Posidonia oceanica* is the iconic seagrass species endemic to the Mediterranean (Procaccini et al. 2001). This climax species rarely forms mixed beds with *Z. marina.*

Nanozostera noltei (also known as *Zostera noltii* in the ecological literature; see also Coyer et al. 2013 regarding the taxonomic change to *Nannzostera*) is a Tethyan species arriving in the Mediterranean and spreading to inland seas of the region some 14-11 mya. It survived the Mediterranean salinity crisis (6-5 mya). It occurs along East Atlantic shores as far as northern Scotland and southern Norway (56°N). Although it occurs in mixed stands with *Z. marina* in some areas (Laugier et al. 1999), its small size (maximal canopy height 15 cm) generally confines it to the intertidal zone where it cannot provide the same habitat function as dense and large lower intertidal and subtidal meadows of *Zostera marina*. When occurring together, *Z. marina* is the canopy forming species attaining about 3-times the plant height compared to *Nanozostera noltii*.

Chefaoui, R. M., Assis, J., Duarte, C. M. & Serrão, E. A. (2016). Large-scale prediction of seagrass distribution integrating landscape metrics and environmental factors: the case of *Cymodocea nodosa* (Mediterranean–Atlantic). *Estuaries and Coasts, 39*, 123-137. doi:10.1007/s12237-015-9966-y

Coyer, J. A., Hoarau, G., Kuo, J., Tronholm, A., Veldsink, J. & Olsen, J. L. (2013). Phylogeny and temporal divergence of the seagrass family Zosteraceae using one nuclear and three chloroplast loci. *Systematics and Biodiversity, 11*(3), 271-284. doi:10.1080/14772000.2013.821187

Ferguson, R. L., Pawlak, B. T. & Wood, L. L. (1993). Flowering of the seagrass *Halodule wrightii* in North Carolina, USA. *Aquatic Botany, 46*(1), 91-98. doi:10.1016/0304-3770(93)90066-6

Koch, E. W. & Orth, R. J. (2003). The seagrasses of the mid-Atlantic coast of the United States. In E. P. Green & F. T. Short (Eds.), *World Atlas of Seagrasses*. Berkeley, California: University of California Press.

Laugier, T., Rigollet, V. & de Casabianca, M.-L. (1999). Seasonal dynamics in mixed eelgrass beds, *Zostera marina* L. and *Z. noltii* Hornem., in a Mediterranean coastal lagoon (Thau lagoon, France). *Aquatic Botany, 63*(1), 51-69. doi:10.1016/S0304-3770(98)00105-3

Lee, K.-S.; Park, S.R. & Kim, Y.K. (2007) Effects of irradiance, temperature, and nutrients on growth dynamics of seagrasses: A review. *Journal of Experimental Marine Biology and Ecology*, *350*, 144-175 doi https://doi.org/10.1016/j.jembe.2007.06.016

Procaccini, G., Orsini, L., Ruggiero, M. V. & Scardi, M. (2001). Spatial patterns of genetic diversity in *Posidonia oceanica*, an endemic Mediterranean seagrass. *Molecular Ecology, 10*(6), 1413-1421. doi:10.1046/j.1365-294X.2001.01290.x

Temperate-to-Arctic North Pacific seagrass species

On the northeastern temperate-to-Arctic Pacific coast, *Z. marina* dominates soft bottoms in monospecific stands from northern Alaska (Bering Sea) and the southwestern Alaskan peninsula (roughly Unimak Pass). It was reported on Atka Island (McRoy 1968) but not further west along the Aleutian chain (attributed to lack of habitat). In southeastern Alaska it extends east and south along the Gulf of Alaska to Juneau, Ketchikan and then along the British Columbian coast to Vancouver and Washington State. From the outer coast of Washington State, there are relatively isolated patches of *Z. marina* found along the Oregon and northern California coast (Humboldt Bay) and then intermittently all the way to Baja California Mexico (Wyllie-Echeverria and Ackerman 2003). *Ruppia maritima* is sometimes found with *Z. marina* but generally restricted to lower salinity lagoons. *Zostera japonica* and *Zostera asiatica* are human introductions from the early 20th century. Three species of *Phyllospadix* (known as surfgrasses) are found from the Chirikof Island in the western Kodiak Archipelago (*P. serrulatus*) through the Alaskan panhandle to Baja, where they dominate the rocky surf zone as their unique niche. The subtropical *Halodule wrightii* is occasionally encountered and restricted to the Gulf of California, Mexico.

The northwestern temperate-to-Arctic Pacific coast from roughly mid-Japan (~36°N) northward is a diversity hotspot for seagrasses (Aioi and Nakaoka 2003) in general and *Zostera* in particular—with four species of *Zostera* and two of *Phyllospadix. Z. japonica* is a small plant found from SW Japan to Sakhalin and the East coast of Kamchatka, Russia. Mixed meadows of 3-4 seagrass species are common in both the tropical and temperate regions where zonation by depth plays a role; *Z, marina* is typically shallow subtidal in this assemblage. From Hokkaido north (including Sakhalin, the Kuril Islands, Kamchatka and the Siberian Beringia) the extent of *Z. marina* is unknown, but a few *Z. marina* patches have been identified (Figure 6a) (Bobrov 2020, personal communication; Krause-Jensen et al. 2020).

Aioi, K. & Nakaoka, M. (2003). The seagrasses of Japan. In E. P. Green & F. T. Short (Eds.), *World Atlas of Seagrasses*. Berkeley, California: University of California Press.

Krause-Jensen, D., Archambault, P., Assis, J., Bartsch, I., Bischof, K., Filbee-Dexter, K., . . . Sejr, M. K. (2020). Imprint of climate change on pan-Arctic marine vegetation. *Frontiers in Marine Science, 7*, 617324. doi:10.3389/fmars.2020.617324

McRoy, C. P. (1968). The distribution and biogeography of *Zostera marina* (eelgrass) in Alaska. *Pacific Science, 22*(4), 507-513.

Wyllie-Echeverria, S. & Ackerman, J. D. (2003). The Pacific coast of North America. In E. P. Green & F. T. Short (Eds.), *World Atlas of Seagrasses*. Berkeley, California: University of California Press.

Dispersal ecology of *Zostera marina*

Genes of seagrasses (McMahon et al. 2014), including eelgrass, can move by three principal mechanisms, lateral clonal growth, pollen movement (Ackerman 2000; Ruckelshaus 1996), and seed-based dispersal (Harwell & Orth 2006; Orth et al. 2002). For the latter, rafting, seed-bearing shoots have been shown to be the predominant mechanism. As for the potential dispersal distances, rafting see-bearing shoots can travel 10-100 kms, while pollen moves only several meters, and clonal growth results in a spread of a particular genotype of dm*yr^{-1}. Hence, the processes discussed in this study most likely result from seed-based dispersal in the form of rafting shoots.

Ackerman, J. D. (2000). Abiotic pollen and pollination: ecological, functional, and evolutionary perspectives. *Plant Systematics and Evolution, 222*(1/4), 167-185.

Harwell, M. C. & Orth, R. J. (2002). Long-distance dispersal potential in a marine macrophyte. *Ecology, 83*(12), 3319-3330. doi:10.1890/0012-9658(2002)083[3319:LDDPIA]2.0.CO;2
McMahon, K., van Dijk, K.-j., Ruiz-Montoya, L., Kendrick, G. A., Krauss, S. L., Waycott, M., . . . Duarte, C. (2014). The movement ecology of seagrasses. *Proceedings of the Royal Society B: Biological Sciences, 281*(1795), 20140878. doi:10.1098/rspb.2014.0878
Orth, R. J., Harwell, M. C. & Inglis, G. J. (2006). Ecology of seagrass seeds and dispersal strategies. In A. W. D. Larkum, R. Orth, & C. M. Duarte (Eds.), *Seagrasses: biology, ecology and conservation* (pp. 111-133). Dordrecht, Netherlands: Springer.
Reusch, T. B. H., Stam, W. T. & Olsen, J. L. (1999). Size and estimated age of genets in eelgrass, *Zostera marina*, assessed with microsatellite markers. *Marine Biology, 133*, 519-525. doi:10.1007/s002270050492
Ruckelshaus, M. H. (1996). Estimation of genetic neighborhood parameters from pollen and seed dispersal in the marine angiosperm *Zostera marina* L. *Evolution, 50*(2), 856-864. doi:10.1111/j.1558-5646.1996.tb03894.x

Supplementary Note 2 - Divergence time estimation using ASTRAL

Constructing an ASTRAL species tree

Illumina sequencing libraries from 190 samples of *Zostera marina* were cleaned (Illumina reads less than 75 bp after trimming for adapter and quality (q < 20) were removed) and de novo assembled using HipMer (k=51). To build suitable gene trees (and a subsequent species tree), transcript sequences from *Z. marina* (v3.1, n = 21,483) were aligned against each HipMer (Georganas et al. 2015) assembly (n=212) using BLAT (v30) (Kent 2002). To find conserved gene sequences in all samples, a transcript was considered 'present' if it had (i) a single alignment greater than 60% identity and coverage; or (ii) a transcript was covered at least to 85% by 3 or fewer alignments among contigs with identity >85%. Next, two quality control assessments were performed prior to gene tree analysis. First, using samples identified as clones, 1016 genes were found to be inconsistently aligned (considered present in one clone but absent in another) and were removed from consideration. Clones were then removed from the dataset, retaining only one representative sample per clone set. Secondly, the total number of genes considered present was counted within all samples, excluding libraries with counts fewer than 17,500 as those were considered fragmented and of too low quality. The final dataset included 129 samples and 20,100 aligned transcripts, comprising 18,311 genes present in 97.9% of all accessions (core gene set). CDS and protein sequences from each transcript alignment were predicted using GeMoMa (v1.7; Keilwagen et al. 2019) using constrained search where each transcript's best alignment location was extracted using bedtools (Quinlan 2014) (getfasta) with 800 bp buffer. A random subset of 617 genes was then selected for building gene trees and the final ASTRAL tree. Admixed samples (populations Washington State and Bodega Bay; discussed in main ms) were not included for gene tree construction. Gene trees were constructed by first aligning CDS sequences together using MAFFT (v7.475; Katoh et al. 2002) (parameters: mafft --localpair --phylipout --maxiterate 1000), then generating individual gene trees with IQTREE (v2.1.2) (Nguyen et al. 2015; parameters -B 1000 -m K2P -T auto). All gene trees were used jointly as input for a species tree analysis with ASTRAL (Zhang et al. 2018) v5.7.3, using a map file to join multiple samples representing the same population.

ASTRAL normalized quartet scores

We also calculated a normalized quartet score for our unrooted ASTRAL tree of 0.48, which suggests high incomplete lineage sorting within the dataset. This is unsurprising considering

that both the multi-species coalescent and StarBEAST2 analyses show that Atlantic populations diverged approximately 20-30 Kya ago and show little nucleotide diversity (Figure 1b). To demonstrate that genotypes within the ASTRAL species tree follow geographic clustering when individuals are unconstrained by a map (-a) file, we ran ASTRAL using default parameters and generated a species tree with each individual colored by geographic origin. As shown in Supplementary Figure 11, despite high ILS the species tree follows geographic patterns with only 2 of 107 individuals showing incongruent topology (CCAXB, corresponding to population ALS; CCBCZ, corresponding to population QU, Supplementary Fig. 11)

Divergence time estimates using StarBEAST2

To investigate the divergence among *Z. marina* populations, the gene alignments (generated by MAFFT as outlined in the ASTRAL species tree section) were analyzed using StarBEAST2 (v2.6.3) (Bouckaert et al. 2014; Heled and Drummond 2010). As age calibration, the divergence between *Z. japonica* and *Z. marina* was estimated using the WGD event that occurred approximately 67 Mya (Olsen et al. 2016) (Supplementary Note 2). Next, protein sequences were predicted from an assembly of *Z. japonica* (Xiaomei Zhang pers. comm., available at doi.org/10.6084/m9.figshare.21626327.v1, and Zhang et al. 2019) using the GeMoMa pipeline outlined above. GeMoMa predicted 8,687 peptide sequences that, when aligned to *Z. marina* peptides using BLAT [v30] (Kent 2002), yielding 7154 reciprocal best orthologs. 4DTV rate calculation among best hit orthologs found a clear peak estimated between 0.0795- 0.0816 (90% CI-from 10,000 bootstrap estimates). Based on the neutral divergence among syntenic paralogs from the *Zostera* WGD event, we obtained a divergence estimate between *Z. japonica* and *Z. marina* at 9.86 - 12.67 mya, consistent with the upper bound of divergence times provided from Coyer et al. (11.8-2.9 mya; Coyer et al. 2013). A median age of 11.01 mya was selected for subsequent StarBEAST2 anaylsis.

The xml file was generated from MAFFT alignments (phylip format) using seqmagick (v0.6.2; Shen et al. 2016) to convert phylip format to nexus. The XML file required for StarBEAST2 was generated using BEAUTi from 70 randomly selected alignments with no missing data in a subset of populations (four samples represented per population): ALS, JS, SD, MA, NN, SW, FR to investigate node ages of major divergences, with *Z. japonica* and *Z. marina* reference sequences. Parameters for the StarBEAST2 run were as follows: gene ploidy = 2; constant population sizes; population size parameter = 0.03 (which assumes a 10K effective population size and a generation time of 3 years); Gamma site model with estimated substitution rate; HKY substitution model; estimate kappa; empirical nucleotide frequencies; strict molecular clock; estimated clock rate; Yule model; Outgroup= *Z. japonica* ; Outgroup divergence constrained with a lognormal prior [M=11.01; S=0.01; mean in real space, use originate]; MCMC chain length = 200,000,000; store every 200,000; pre-burnin = 0. starBEAST was run in triplicate, inspecting each run with Tracer to check for model convergence (Barido-Sottani et al. 2018) (10% burn-in; Effective sample sizes [ESS]>300). TreeAnnotator was to summarize each run (10% burn-in, median peak height, 0.5 posterior probability limit) which were then combined using LogCombiner. Finally, the log combined tree file was re-summarized with TreeAnnotator using the parameters listed above. Note that StarBEAST2 analysis requires an estimate of generation time and population size, both of which are subject to large error in eelgrass owing to large differences in clonal vs. sexual reproduction among populations (see also Supplementary Table 3).

Barido-Sottani, J., Bošková, V., Plessis, L. D., Kühnert, D., Magnus, C., Mitov, V., . . . Stadler, T. (2018). Taming the BEAST—A community teaching material resource for BEAST 2. *Systematic biology, 67*(1), 170-174. doi:10.1093/sysbio/syx060
Bouckaert, R., Heled, J., Kühnert, D., Vaughan, T., Wu, C.-H., Xie, D., . . . Drummond, A. J. (2014). BEAST 2: a software platform for Bayesian evolutionary analysis. *PLoS computational biology, 10*(4), e1003537. doi:10.1371/journal.pcbi.1003537
Coyer, J. A., Hoarau, G., Kuo, J., Tronholm, A., Veldsink, J. & Olsen, J. L. (2013). Phylogeny and temporal divergence of the seagrass family Zosteraceae using one nuclear and three chloroplast loci. *Systematics and Biodiversity, 11*(3), 271-284. doi:10.1080/14772000.2013.821187
Georganas, E., Buluç, A., Chapman, J., Hofmeyr, S., Aluru, C., Egan, R., . . . Yelick, K. (2015). *HipMer: an extreme-scale de novo genome assembler.* Paper presented at the Proceedings of the International Conference for High Performance Computing, Networking, Storage and Analysis.
Heled, J. & Drummond, A. J. (2010). Bayesian inference of species trees from multilocus data. *Molecular biology and evolution, 27*(3), 570-580. doi:10.1093/molbev/msp274
Katoh, K., Misawa, K., Kuma, K. i. & Miyata, T. (2002). MAFFT: a novel method for rapid multiple sequence alignment based on fast Fourier transform. *Nucleic acids research, 30*(14), 3059-3066. doi:10.1093/nar/gkf436
Keilwagen, J., Hartung, F. & Grau, J. (2019). GeMoMa: homology-based gene prediction utilizing intron position conservation and RNA-seq data. In M. Kollmar (Ed.), *Gene prediction: Methods and protocols* (pp. 161-177). New York, NY: Humana.
Kent, W. J. (2002). BLAT—the BLAST-like alignment tool. *Genome research, 12*(4), 656-664. doi:10.1101/gr.229202
Lovell, J. T., Sreedasyam, A., Schranz, M. E., Wilson, M., Carlson, J. W., Harkess, A., . . . Schmutz, J. (2022). GENESPACE tracks regions of interest and gene copy number variation across multiple genomes. *Elife, 11*, e78526. doi:10.7554/eLife.78526
Nguyen, L.-T., Schmidt, H. A., Von Haeseler, A. & Minh, B. Q. (2015). IQ-TREE: a fast and effective stochastic algorithm for estimating maximum-likelihood phylogenies. *Molecular biology and evolution, 32*(1), 268-274. doi:10.1093/molbev/msu300
Olsen, J. L., Rouzé, P., Verhelst, B., Lin, Y.-C., Bayer, T., Collen, J., . . . Van De Peer, Y. (2016). The genome of the seagrass *Zostera marina* reveals angiosperm adaptation to the sea. *Nature, 530*(7590), 331-335. doi:10.1038/nature16548
Quinlan, A. R. (2014). BEDTools: the Swiss-army tool for genome feature analysis. *Current protocols in bioinformatics, 47*(1), 11.12. 11-11.12. 34. doi:10.1002/0471250953.bi1112s47
Shen, W., Le, S., Li, Y. & Hu, F. (2016). SeqKit: a cross-platform and ultrafast toolkit for FASTA/Q file manipulation. *PloS one, 11*(10), e0163962. doi:10.1371/journal.pone.0163962
Zhang, C., Rabiee, M., Sayyari, E. & Mirarab, S. (2018). ASTRAL-III: polynomial time species tree reconstruction from partially resolved gene trees. *BMC bioinformatics, 19*(6), 15-30. doi:10.1186/s12859-018-2129-y
Zhang, X., Zhou, Y., Li, Y.-L. & Liu, J.-X. (2019). Development of microsatellite markers for the seagrass *Zostera japonica* using next-generation sequencing. *Molecular biology reports, 46*, 1335-1341. doi:10.1007/s11033-018-4491-2

Supplementary Note 3 - Geographic origin of the Zosteraceae and of *Zostera marina*

The geographical origin of a species is determined by multiple criteria including endemism, species richness within the genus relative to other locations, a comprehensive phylogeny that includes biogeographic sampling, divergence time dating, a fossil record if possible, and whatever else may be known about geological events and human introductions. All of these criteria (except a fossil) are available for *Z. marina*.

Seagrasses arose from 3-4 ancestral lineages within the core group of the freshwater Alismatales (basal monocots) during the Middle Cretaceous (ca. 100 mya) in SE Asia (Hartog & Kuo 2006, Les & Tippery 2013, Du et al. 2016). The ancestral split from freshwater relatives is deep (87 mya for Zosteraceae, Kato et al. (2003) and 67 mya Olsen et al. 2016). Modern diversification (i.e., the ancestors of *Phyllospadix* and *Zostera*) is much more recent (48-36 mya, Kato 2003; 23 mya, Coyer et al. 2013). There are no reliable fossils for *Zostera* (McRoy 1968, Kato et al. 2003).

A dated phylogeny for *Zosteraceae* was produced by Coyer et al. (2013) and based on complete species-level sampling, including the biogeographic component. They used four-genes (nuclear ITS, cp *rbc*L, *mat*-K and *psb*A-*trn*H). Dating was calibrated using *Oryza rbc*L

(which correlates well with recent dating based on whole genome duplication paralogues in *Z. marina* (Olsen et al. 2016).

A full taxonomic revision was also provided by Coyer et al. (2013). Their four-locus analysis supported the elimination of subgeneric designations within *Zostera*, which have caused perpetual taxonomic confusion. Molecular divergence among the clades was found to be deep and highly significant, each forming a monophyletic group and in a distinct geographic area. Thus, three genera are recognized: *Zostera* (northern hemisphere only and the ancestral lineage), *Nanozostera* (southern hemisphere, found in Australia [noting Tethyan dispersal to the Mediterranean and later NE Atlantic explained below] only and a derived lineage) and *Heterozostera* (southern hemisphere and a derived lineage).

Z. marina almost certainly evolved in the Japanese Archipelago region when it split off from the Chinese mainland some 19-15 mya (Barnes 2003, Tanaka et al. 2009). Diversification of *Z. asiatica* occurred after the period of island formation and habitat fracturing, conditions that led to eventual speciation. This conclusion is supported by species richness and endemism of both the genus and several species. Specifically, *Z. asiatica* diverged first at 4.6 Ma in the Japanese Archipelago (Green & Short, 2003; Coyer *et al.*, 2008). *Z. asiatica, Z. caespitosa*, *Z. caulescens, and Z. japonica* are all Japanese/Korean/Chinese endemics (although both *Z. asiatica* and *Z. japonica* are known introductions within the past century to the Pacific Northwest).

It is important to note that the northern-southern hemisphere split between *Zostera* and *Nanozostera* occurred much earlier (between 23-15 mya) in accordance with final closure of the Tethys Sea and major tectonic events that swept the ancestral lineages to the nascent Mediterranean and south to Australia. There are many endemic species of *Nanozostera* in Australia.

Barnes, G. L. (2003). Origins of the Japanese Islands: The New" Big Picture". *Japan Review, 15*, 3-50.

Coyer, J. A., Hoarau, G., Kuo, J., Tronholm, A., Veldsink, J. & Olsen, J. L. (2013). Phylogeny and temporal divergence of the seagrass family Zosteraceae using one nuclear and three chloroplast loci. *Systematics and Biodiversity, 11*(3), 271-284. doi:10.1080/14772000.2013.821187

Den Hartog, C. & Kuo, J. (2006). Taxonomy and biogeography of seagrasses. In A. W. D. Larkum, R. J. Orth, & C. M. Duarte (Eds.), *Seagrasses: biology, ecology and conservation* (pp. 1-23). Dordrecht: Springer.

Du, Z. Y., Wang, Q. F. & Consortium, C. P. (2016). Phylogenetic tree of vascular plants reveals the origins of aquatic angiosperms. *Journal of Systematics and Evolution, 54*(4), 342-348. doi:10.1111/jse.12182

Green, E. P. & Short, F. T. (2003). *World atlas of seagrasses*. CA, USA: University of California Press.

Kato, Y., Aioi, K., Omori, Y., Takahata, N. & Satta, Y. (2003). Phylogenetic analyses of *Zostera* species based on *rbcL* and *matK* nucleotide sequences: implications for the origin and diversification of seagrasses in Japanese waters. *Genes & genetic systems, 78*(5), 329-342. doi:10.1266/ggs.78.329

Les, D. H. & Tippery, N. P. (2013). In time and with water... the systematics of alismatid monocotyledons. In P. Wilkin & S. J. Mayo (Eds.), *Early events in monocot evolution* (pp. 118-164). Cambridge, UK: Cambridge University Press.

Tanaka, N., Aida, S., Akaike, S., Aramaki, H., Chiyokubo, T., Chow, S., . . . Saitoh, K. (2009). Distribution of *Zostera* species in Japan. I. *Zostera marina* L.(Zosteraceae). *Bull Nat Mus Nat Sci Series B (Botany), 35*(1), 23-40.

Waycott, M., Biffin, E. & Les, D. H. (2018). Systematics and evolution of Australian seagrasses in a global context. In A. Larkum, G. Kendrick, & P. Ralph (Eds.), *Seagrasses of Australia: Structure, Ecology and Conservation* (pp. 129-154): Springer.

Supplementary Note 4 - Rate estimation of the synonymous molecular clock in *Zostera marina*

Estimating the divergence time between *Zostera japonica* and *Z. marina*

To calibrate a molecular clock for *Z. marina*, we first calculated the fourfold degenerative third-codon transversion rate (4DTv) for each of the 1,072 syntenic paralog pairs in the *Z. marina* reference V3.1 (as determined with the comparative genomics pipeline

GENESPACE; Schmutz & Lovell, 2021). The 4DTv rates showed a distinct peak at approximately 0.48, indicating the *Z. marina* whole genome duplication event (~67 mya; Olsen et al., 2016). The 90% confidence interval of the peak (0.4640-0.5164) was estimated based on 10,000 bootstraps.

The divergence time between *Z. marina* and *Z. japonica* was then estimated based on the divergence of homologs. A constrained protein homology search was performed using BLAT and GeMoMa (v1.7; Keilwagen et al., 2019). To generate a constrained sequence database to search, *Z. marina* transcripts were aligned to the *Z. japonica* genome assembly. Each transcript's best hit location (+500 bp sequence buffer) was extracted from the *Z. japonica* genome and was used for GeMoMa protein prediction with default parameters. The protein prediction pipeline found 8,687 peptide sequences. Gffread (Pertea & Pertea, 2020) was used to extract each peptide coding sequence, and 1:1 orthologs (n=7,154) between *Z. japonica* and *Z. marina* were identified using best reciprocal BLAT hits. The 4DTv rate among orthologs was calculated and estimated using 10,000 bootstrap estimates (0.0795-0.0816; 90% CI). Applying the WGD age to the 4DTv neutral rate, the divergence time between *Z. japonica* and *Z. marina* was estimated to be between 9.86 - 12.67 MYA, which is consistent with the upper bound estimate of 11.9 mya from Coyer et al. (2013).

Estimating the mutation rate

The divergence time between *Z. japonica* and *Z. marina* was represented as a lognormal distribution (Mean: 11.1542 MYA; SD: 0.07). An estimate of a neutral mutation rate was calculated based on the synonymous mutation rate (K_s) among *Z. japonica* orthologs and four *Z. marina* populations (JS, BB, NC, and FR). The median Ks peak between *Z. japonica* and *Z. marina* was approximately 0.211, which was consistent across all 4 populations. The neutral mutation rate was calculated as $r = K_s / (2 * \text{divergence age}) = 9.462 * 10^{-9}$ per generation per year.

SNP calling and filtering including *Z. japonica*

One *Z. japonica* sample was included for genetic variant calling. The GVCF format file for *Z. japonica* was generated in the same way with the *Z. marina*. All the GVCF files (190 *Z. marina* samples + 1 *Z. japonica*) were combined by CombineGVCFs (GATK4). GenotypeGVCFs (GATK4) was used to call genetic variants. BCFtools was used to remove SNPs within 20 base pairs of an indel or other variant type. Then we extracted only SNPs (10,562,762 SNPs), and excluded the other types of variants. VariantsToTable (GATK4) was used to extract INFO annotations. SNPs meeting one or more than one of the following criteria were marked by VariantFiltration (GATK4): MQ < 40.0; FS > 60.0; QD < 10.0; MQRandSum > 2.5 or MQRandSum < -2.5; ReadPosRandSum < -2.5; ReadPosRandSum > 2.5; SOR > 3.0; DP > 11185.0 (2 * average DP). Those SNPs were excluded by SelectVariants (GATK4). A total of 4,873,274 SNPs were retained. VCFtools was used to convert individual genotypes to missing data when GQ < 30 or DP < 10. Individual homozygous reference calls with one or more read supporting the variant allele, and individual homozygous variant calls supporting the reference allele, were also converted to missing data using a custom Python3 script. Only bi-allelic SNPs were kept (4,775,984 SNPs). To avoid the reference-related biases, we focused on the core genes shared by all individual samples. Bedtools was used to find overlap between the SNPs and the core genes in the six main chromosomes (18,717 genes), and only those SNPs were kept (1,483,603 SNPs). Thirty-seven ramets were excluded from ZM_HQ_SNPs, including 10 ramets related to geitonogamous (within-clone) selfing (Supplementary Table 2), 17 ramets representing

replicated genotypes (Supplementary Table 3), and 10 ramets with high missing rate (Supplementary Fig. 1). After excluding these ramets, some SNPs became monomorphic, which were then excluded. SnpEff was used to annotate each SNP. To obtain putatively neutral SNPs, we only kept SNPs annotated as "synonymous_variant" (ZMZJ_Neutral_SNPs, 171,756 SNPs). Then only SNPs without any missing data were kept (ZMZJ_D_SNPs, 20,341 SNPs).

Coyer, J. A., Hoarau, G., Kuo, J., Tronholm, A., Veldsink, J. & Olsen, J. L. (2013). Phylogeny and temporal divergence of the seagrass family Zosteraceae using one nuclear and three chloroplast loci. *Systematics and Biodiversity, 11*(3), 271-284. doi:10.1080/14772000.2013.821187

Keilwagen, J., Hartung, F. & Grau, J. (2019). GeMoMa: homology-based gene prediction utilizing intron position conservation and RNA-seq data. *Gene prediction: Methods and protocols*, 161-177.

Olsen, J. L., Rouzé, P., Verhelst, B., Lin, Y.-C., Bayer, T., Collen, J., . . . Van De Peer, Y. (2016). The genome of the seagrass *Zostera marina* reveals angiosperm adaptation to the sea. *Nature, 530*(7590), 331-335. doi:10.1038/nature16548

Pertea, G. & Pertea, M. (2020). GFF utilities: GffRead and GffCompare. *F1000Research, 9*.

Schmutz, J. & Lovell, J. (2021). *GENESPACE R Package (GENESPACE) v1. 0*. Computer software. https://www.osti.gov//servlets/purl/1787849. USDOE. 11 May. 2021. Web. doi:10.11578/dc.20210610.3.

Supplementary Note 5 - The Role of the North Pacific Current (NPC) gateway for eelgrass population admixture

The strongly flowing North Pacific Current (NPC) approaches the East Pacific Coast at the latitude of mid-Vancouver Island, but varies between roughly Portland (Oregon) (45°N) and the Alaskan-British Columbian border (53°N, see inset maps 1 and 2). At the bifurcation, ~60% of the flow goes north, becoming part of the Alaskan Current (AC) while 40% of the water masses move south, becoming the California Current (CC). The precise fraction of the flow going in each direction varies as a function of other forcing including El Niño Southern Oscillation (ENSO) status (Checkley and Barth 2009). Thus, trans-Pacific dispersers can end up in very different places—Alaska or California. South of the bifurcation from Washington state to central California, exposed coastline is punctuated by widely-spaced smaller embayments that are suitable habitat for eelgrass. North of the bifurcation there is more continuous suitable eelgrass habitat including the inner waters of the Salish Sea and the Inside Passage of coastal BC and AK.

From Washington State south, the CC and coastal jet result in transport from north to south most of the year. The coastal jet is a wind-driven surface current, 50-90 km wide, that is strongest from Spring through Summer and weakest in the winter months when these winds relax. Northward flow at the surface occurs seasonally as the Davidson Current (DC) in winter months (Checkley and Barth 2009) and can enter the tidally modulated Strait of Juan de Fuca. Local eddies, periodic El Niño effects and exceptional storms can further affect transport. Reversal of the typical equatorward flow during Spring and Summer in exceptional El Niño years can result in northward range expansions of coastal species of 100s of km, with some establishing breeding populations beyond previous range limits (Sanford et al. 2019, Yamada et al. 2021).

The Strait of Juan de Fuca eddy, the so called "Big Eddy", in combination with the DC and tidal action in the Strait of Juan de Fuca, affect what will be transported in and out of the inner waters of British Columbia and Washington State (the Salish Sea). In addition, estuarine conditions of the inner waters are greatly affected by the Fraser River outflow, which exits both north in to the Strait of George and west along the northern side of the Strait of Juan de Fuca.

As noted in the main text, the LGM reduced eelgrass habitat along the BC and Alaska coast, e.g., Haida Gwaii, was glaciated as was much of southeastern Alaska. However, it is

also well-established that many rocky shore marine organisms and seaweeds survived in high latitude, coastal refugia, in part due to the wider shelf (Fig. 6a, green dots, Jacobs 2004, Marko et al. 2010, Bringloe et al. 2020).

In our study, we did not sample the SE Alaskan panhandle and British Columbian coast (dotted box, inset map 1), nor the inner areas of the Salish Sea and Puget Sound, where *Z. marina* is abundant. The question is, “What new insights might be gained if such sampling was added to our analysis?”

We speculate that some dispersers arriving from the west via the NPC would be deflected north along the outer coast of Vancouver Island making their way along the AC. From there they continue through the porous islands of Alaskan peninsula and north to the Bering Strait area (discussed in detail in Talbot et al. 2016). The other dispersers arriving from the west via the NPC would be deflected south catching the CC or coastal jet. The DC, when flowing north and towards the shore, would push dispersers (i.e., between Humboldt Bay-California and the outer coast of Washington) through the Strait of Juan de Fuca, the Salish Sea and Puget Sound. Countering this directionality is the outflow of the Fraser River, which pushes west out of the Strait of Juan de Fuca along the outer coast of Vancouver Island. Coastal currents through the Strait of Georgia will also push dispersers north via the Inside Passage.

Further support for the gateway bifurcation and shelf-break effects comes from the *Z. marina* study of Talbot et al. (2016; Supplement) and the distribution of two chloroplast *mat*-K haplotypes (see inset map 3). Both were present in northern Hokkaido-Japan, but on the East Pacific side, the *mat*-K2 haplotype was only found north in all of the Bering large marine ecosystem (LME) (5 sites) and Gulf of Alaska-LME (6 sites)—but also noting the absence of sampling along the BC coast. *Mat*-K2 was also found in Puget Sound-Washington, Yaquina-Oregon and Humboldt Bay-California (but not further south). The second haplotype, *mat*-K4 was also found in Puget Sound, was absent from Yaquina-Oregon, but present in Humboldt Bay-California and six further sites extending all the way to Baja-California. These results illustrate the importance of the Gateway and shelf break area in separating the northern and southern distribution of *Z. marina*.

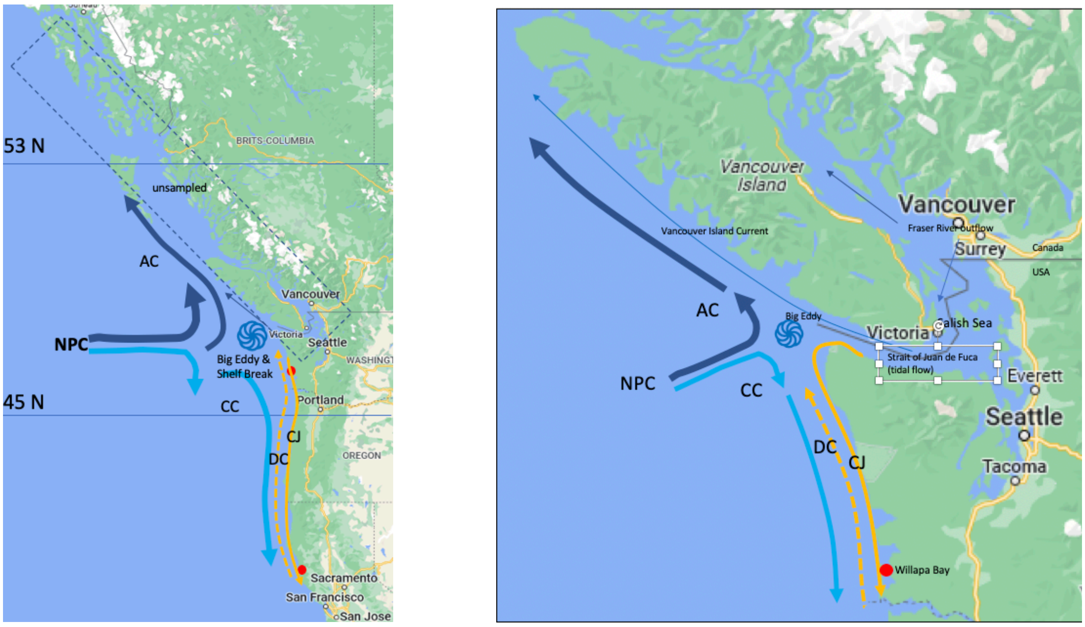

Inset maps of the NPC Gateway and associated currents. Above Left Inset 1: Overview of the larger East Pacific coast. The latitudinal lines bracket the north-south movement of the NPC bifurcation. The dotted box represents the unsampled area of *Z. marina* in the present study. **Above Right Inset 2**: Enlarged view of inner waters. NPC=North Pacific Current; AC=Alaska Current; CC=California Current; DC=Davidson Current (dotted); CJ=Coastal Jet; Swirled symbol=Big Eddy and continental shelf break; Red dots=Sampled populations in the present study. Map source: Google (n.d.). [Google Maps, NE Pacific coast from Washington state to the Alaskan panhandle]. Retrieved 11 April 2023, from URL https://www.google.com/maps/@50.3735115,-122.8748009,6.86z. Figure by Olsen JL (co-author).

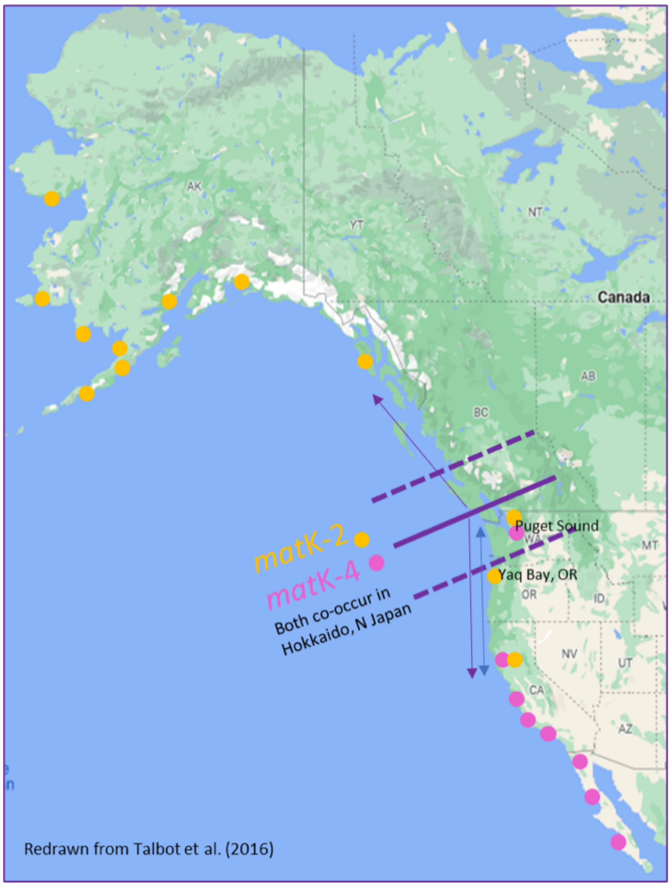

Inset map 3: Distribution of chloroplast *mat*-K haplotypes from Talbot et al. (2016). The solid purple line is the NPC gateway and "Big Eddy" off the coast of Vancouver Island. Dotted purple lines bracket the NPC's latitudinal variation. Arrows north and south indicate the bifurcation. The blue double-ended arrow is the variable Davidson Current and Coastal Jet. Redrawn from Talbot et al. (2016). Source: Google (n.d.). [Google Maps, NE Pacific coast from Baja to the Alaskan peninsula base map for Inset 3]. Retrieved 11 April 2023, from URL https://www.google.com/maps/@61.2142059,-128.9090232,4.26z. Figure by J.L. Olsen (co-author) based on data from S. Talbot et al. (2016), co-author.

Bringloe, T. T., Verbruggen, H. & Saunders, G. W. (2020). Unique biodiversity in Arctic marine forests is shaped by diverse recolonization pathways and far northern glacial refugia. *Proceedings of the National Academy of Sciences* 117, 22590-22596. doi:10.1073/pnas.2002753117.

Checkley, D.M. & Barth, J.A. (2009). Patterns and processes in the California Current System. *Progress in Oceanography* 83, 49–64

Jacobs, D. K., Haney, T. A. & Louie, K. D. (2004). Genes, diversity, and geologic process on the Pacific coast. *Annual Reviews Earth and Planetary Science, 32*, 601-652. doi:10.1146/annurev.earth.32.092203.122436

Lewis, A. G., Stark, K. & O'Connor, M. (2022). Travelogues of two copepod species in coastal waters of British Columbia. *Crustaceana, 95*(3), 289-305. doi:10.1163/15685403-bja 10183

Marko, P. B., Hoffman, J. M., Emme, S. A., McGovern, T. M., Keever, C. C. & Nicole Cox, L. (2010). The 'Expansion–Contraction'model of Pleistocene biogeography: rocky shores suffer a sea change? *Molecular Ecology, 19*(1), 146-169. doi:10.1111/j.1365-294X.2009.04417.x (2010)

Sanford, E., Sones, J.L., García-Reyes, M. *et al.* (2019). Widespread shifts in the coastal biota of northern California during the 2014–2016 marine heatwaves. *Scientific Reports,* 9, 4216 doi.org/10.1038/s41598-019-40784-3

Talbot, S. L., Sage, G. K., Rearick, J. R., Fowler, M. C., Muñiz-Salazar, R., Baibak, B., . . . Ward, D. H. (2016). The structure of genetic diversity in eelgrass (*Zostera marina* L.) along the north Pacific and Bering Sea coasts of Alaska. *PLoS One, 11*(4), e0152701. doi:10.1371/journal.pone.0152701

Yamada, S. B., Gillespie, G. E., Thomson, R. E. & Norgard, T. C. (2021). Ocean indicators predict range expansion of an introduced species: invasion history of the European green crab *Carcinus maenas* on the North American Pacific coast. *Journal of Shellfish Research, 40*(2), 399-413. doi:10.2983/035.040.0212

Supplementary Note 6 - Using the cpDNA haplotype clock as an alternative dating estimate

Estimating chloroplast haplotype divergence time

We used chloroplast synonymous substitutions to validate the divergence time between the SD and BB populations and the rest of the Pacific. The shortest synonymous mutation distance (D_{Ss}) between the two groups is 10 mutations, for example, between SD10 and WAS03. The longest distance (D_{Sl}) is 13 mutation steps, for example, between haplotypes represented in SD11 and JS03. The total of 9731 synonymous sites in chloroplast protein-coding genes (S) was calculated based on a custom-made annotation. The chloroplast synonymous substitution rate (K_S) range of 1.1 – 2.9 x 10^{-9} substitutions per site per year was obtained from Muse (2000). The divergence time of the Californian and the main Pacific chloroplasts is expected to fall between the fastest and the slowest evolution scenario, which are D_{Ss} / (2 * K_{Smax} * S) = 0.177 Mya and D_{Sl} / (2 * K_{Smin} * S) = 0.607 Mya correspondingly. Thereby the average expected divergence time based on chloroplast synonymous substitutions is 0.392 Mya which is in accordance to the nuclear-based calculated divergence time (0.3525 Mya).

Muse, S. V. (2000). Examining rates and patterns of nucleotide substitution in plants. *Plant Molecular Biology, 42*, 25-43. doi:10.1023/A:1006319803002

Supplementary Note 7 - Eelgrass refugia during the last glacial maximum in the Atlantic area

Marine refugia during the last LGM

The LGM led to latitudinal shifts in marine species distributions southward into ice free areas of the coast in combination with a greatly lowered sea level and an exposed continental shelf. In addition, the differential positions of SST contours between the West and East Atlantic further facilitated possibilities for rocky/soft sediment intertidal/subtidal refugia (Inset 1 and Fig. 6b-green ovals) as reviewed in Maggs et al. (2008) and Jenkins et al. (2018). At the end of the LGM the East Atlantic coast (Inset 2) was drastically altered, as the nascent North Sea basin filled. By contrast, the more linear western Atlantic coast, below Long Island-New York was scarcely affected, while the northern coast from the Gulf of Maine to Newfoundland was greatly affected (Inset 3). In general, rapid post-LGM expansions following the "southern richness - northern purity" model (Hewitt 2000) have been found to be the rule rather than the exception (e.g., reviewed in Jenkins et al. 2018).

Eelgrass refugia in the Atlantic

As a broadly distributed temperate to Arctic species, eelgrass found refugia in many of the known locations for other marine organisms on both sides of the Atlantic (Olsen et al. 2004, Campanella et al. 2010); but see also Becheler et al. 2010 [Brittany], Jahnke et al. 2018 [Skagerrak, W Sweden, E Denmark]). Based on microsatellite loci and on sampling locations taken from different studies, suggests at best, a weak decrease in diversity on the East Atlantic coast with latitude and a strong one on the West Atlantic coast (Inset 4).

Along the East Atlantic, the lowest diversity values are indeed at the distributional edges—rear edge in southern Portugal (Billingham et al. 2003, Diekmann & Serrão 2012), northern Norway (Olsen et al. 2013, ZEN dataset) and Baltic Finland (Reusch et al. 1999b, Johannesson & André 2006). The highest diversity is found in Wadden Sea-North Sea around Sylt and Schleswig-Holstein-Skagerrak-Kattegat regions, a genetic diversity hotspot for eelgrass that did not exist until ~5000 kya (Olsen et al. 2004, Ferber et al. 2009, Jahnke et al. 2018 and ZEN dataset). Further south, in Brittany a mosaic of high and low genetic variability was found by Becheler et al. (2010), with some locations showing diversity suggestive of a refuge and others suggestive of a rear edge. South of the Bay of Biscay there was little shelf exposed along the Atlantic coast of Portugal and eelgrass is/was known only from the southern coast (The Ria Formosa) where it has since become extinct.

Along the West Atlantic, the highest diversities are in the North Carolina and Chesapeake Bay region (Campanella et al. 2010), consistent with a southern refuge that escaped the LGM, in large measure, to the compressed SST contours. Above Long Island and up through northern Maine (Campanella et al. 2010, Olsen et al. 2004) genetic diversity decreases.

So far, there have been no studies that include the high north coasts of the Canadian Maritimes, Atlantic Canada-Newfoundland and southern Greenland above 50°N. Given the reported patchiness of diversity values from Brittany (Becheler et al. 2010), only a very dense sampling of both north and south Brittany might resolve the issue of how much the East Atlantic contributed to recolonization of itself following the LGM.

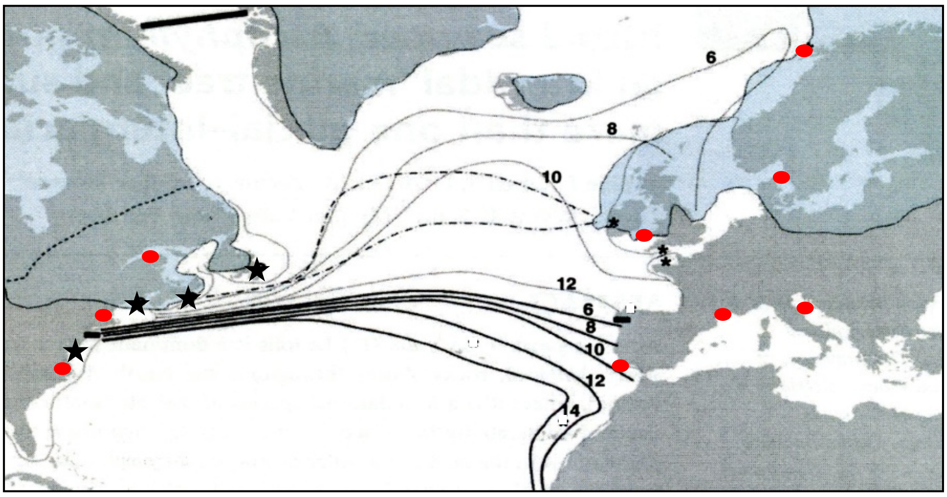

Inset 1. Exposed Atlantic coastlines, glacial cover and sea surface temperature isotherms (SST) at the LGM. Upper grey lines: modern SST contours; lower black lines: LGM SST contours; dotted lines: perennial sea ice during the LGM (adapted from CLIMAP); stars: documented refugia; red dots: sampling from the present study. Map prepared by J.L. Olsen (co-author) based on https://printable-maps.blogspot.com/2008/07/printable-blank-world-map.html. Surface isotherms are based on CLIMAP (1981) Seasonal reconstructions of the Earth's surface at the last glacial maximum. Geological Society of America Map Chart Series MC-36. Geological Society of America, Boulder, CO, USA.

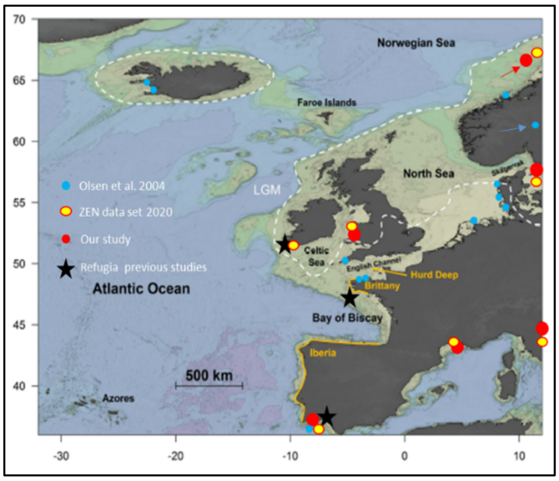

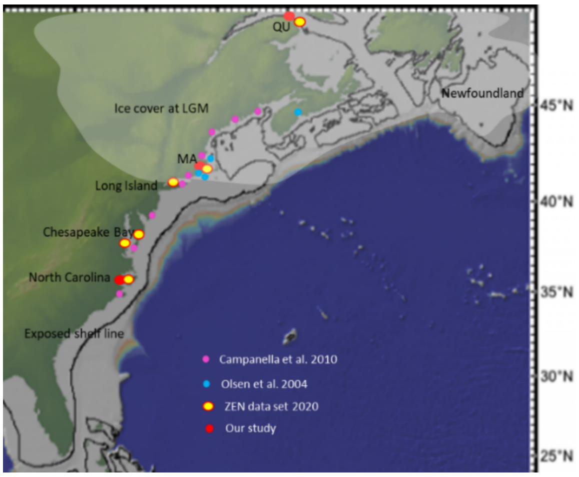

Inset 2 top left. East Atlantic. Detailed view of sea level emphasizing habitat availability during the LGM. Stars, documented refugia; red dots, sampling in the present study.; yellow dots, ZEN dataset 2020; blue dots, Olsen et al. (2004). Map from Jenkins et al. (2018), Peer J. Open access published under CC BY 4.0.

Inset 3 top right. West Atlantic. More detailed view of sea level and coast emphasizing habitat availability during the LGM. White overlay, glacial edges; red dots, sampling from the present study; yellow dots, ZEN dataset (2020); pink dots (Campanella et al. 2010). The Atlantic coast was exposed below Long Island-New York and along the eastern tip of Newfoundland. Base map from Bice, D. [n.d.], Penn. State Univ. licensed under Creative commons: CC BY-NC-SSA 4.0. URL: https://www.e-education.psu.edu/earth103/node/731 ; modified by J.L. Olsen (co-author)

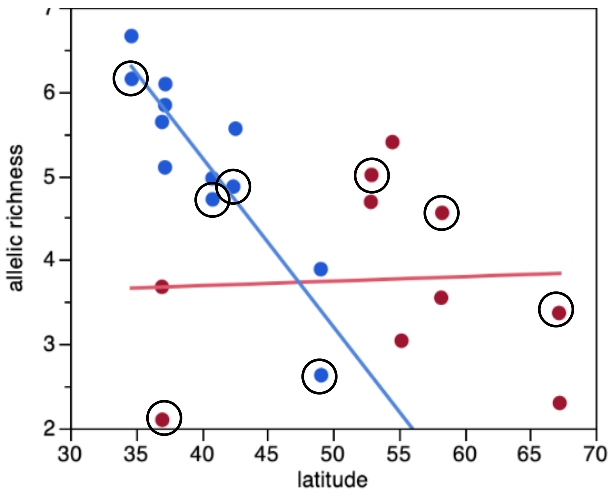

Inset 4 left. Plots of allelic richness (AR) against latitude based on ZEN dataset-2020 (see references) based on 24 microsatellite loci and rarefaction N=7. Blue dots, West Atlantic; red dots, East Atlantic. Circled locations represent locations used in the present study.

Becheler, R., Diekmann, O., Hily, C., Moalic, Y. & Arnaud-Haond, S. (2010). The concept of population in clonal organisms: mosaics of temporally colonized patches are forming highly diverse meadows of *Zostera marina* in Brittany. *Molecular Ecology, 19*(12), 2394-2407. doi:10.1111/j.1365-294X.2010.04649.x

Billingham, M. R., Reusch, T. B. H., Alberto, F. & Serrão, E. A. (2003). Is asexual reproduction more important at geographical limits? A genetic study of the seagrass *Zostera marina* in the Ria Formosa, Portugal. *Marine Ecology Progress Series, 265*, 77-83. doi:10.3354/meps265077

Campanella, J. J., Bologna, P. A., Smalley, J. V., Avila, D. N., Lee, K. N., Areche, E. C. & Slavin, L. J. (2013). An analysis of the population genetics of restored *Zostera marina* plantings in Barnegat Bay, New Jersey. *Population Ecology, 55*, 121-133. doi:10.1007/s10144-012-0351-4

Campanella, J. J., Bologna, P. A., Smalley, J. V., Rosenzweig, E. B. & Smith, S. M. (2010). Population structure of *Zostera marina* (eelgrass) on the western Atlantic coast is characterized by poor connectivity and inbreeding. *Journal of Heredity, 101*(1), 61-70. doi:10.1093/jhered/esp103

Diekmann, O. E. & Serrao, E. A. (2012). Range-edge genetic diversity: locally poor extant southern patches maintain a regionally diverse hotspot in the seagrass *Zostera marina*. *Molecular Ecology, 21*(7), 1647-1657. doi:10.1111/j.1365-294X.2012.05500.x

Hewitt, G. M. (2000). The genetic legacy of the Pleistocene ice ages. *Nature, 405*, 907-913. doi:10.1038/35016000

Jahnke, M., Jonsson, P. R., Moksnes, P. O., Loo, L. O., Jacobi, M. N. & Olsen, J. L. (2018). Seascape genetics and biophysical connectivity modelling support conservation of the seagrass *Zostera marina* in the Skagerrak–Kattegat region of the eastern North Sea. *Evolutionary Applications, 11*(5), 645-661. doi:10.1111/eva.12589

Jenkins, T. L., Castilho, R. & Stevens, J. R. (2018). Meta-analysis of northeast Atlantic marine taxa shows contrasting phylogeographic patterns following post-LGM expansions. *PeerJ, 6*, e5684. doi:10.7717/peerj.5684

Johannesson, K. & Andre, C. (2006). Life on the margin: genetic isolation and diversity loss in a peripheral marine ecosystem, the Baltic Sea. *Molecular Ecology, 15*(8), 2013-2029. doi:10.1111/j.1365-294X.2006.02919.x

Maggs, C. A., Castilho, R., Foltz, D., Henzler, C., Jolly, M. T., Kelly, J., . . . Wares, J (2008). Evaluating signatures of glacial refugia for North Atlantic benthic marine taxa. *Ecology, 89*(sp11), S108-S122. doi:10.1890/08-0257.1

Olsen, J. L., Coyer, J. A., Stam, W. T., Moy, F. E., Christie, H. & Jørgensen, N. M. (2013). Eelgrass *Zostera marina* populations in northern Norwegian fjords are genetically isolated and diverse. *Marine Ecology Progress Series, 486*, 121-132. doi:10.3354/meps10373

Olsen, J. L., Stam, W. T., Coyer, J. A., Reusch, T. B., Billingham, M., Boström, C., . . . Wyllie-Echeverria, S. (2004). North Atlantic phylogeography and large-scale population differentiation of the seagrass *Zostera marina* L. *Molecular Ecology, 13*(7), 1923-1941. doi:10.1111/j.1365-294X.2004.02205.x

Reusch, T. B. H. (2002). Microsatellites reveal high population connectivity in eelgrass (*Zostera marina*) in two contrasting coastal areas. *Limnology and Oceanography, 47*(1), 78-85. doi:10.4319/lo.2002.47.1.0078

Reusch, T. B. H., Boström, C., Stam, W. T. & Olsen, J. L. (1999b). An ancient eelgrass clone in the Baltic. *Marine Ecology Progress Series, 183*, 301-304. doi:10.3354/meps183301

Reusch, T. B. H., Stam, W. T. & Olsen, J. L. (1999a). Microsatellite loci in eelgrass *Zostera marina* reveal marked polymorphism within and among populations. *Molecular Ecology, 8*(2), 317-321. doi:10.1046/j.1365-294X.1999.00531.x

Reusch, T. B. H., Stam, W. T. & Olsen, J. L. (2000). A microsatellite-based estimation of clonal diversity and population subdivision in *Zostera marina*, a marine flowering plant. *Molecular Ecology*, 9(2), 127-140. doi:10.1046/j.1365-294x.2000.00839.x

ZEN *Zostera* Experimental Network genetic microsatellite dataset is available at: https://doi.org/10.5281/zenodo.3660013. M. Jahnke, Data and code for "eelgrass-seascape-ecology-and-genetics" (v1.0.0). Zenodo. https://doi.org/10.5281/zenodo.3660013. Deposited 8 February 2020.

Supplementary Note 8 - *Zostera marina* and *Z. pacifica* in the San Diego to Baja region

Our data raise the question whether the deep divergent San Diego SD lineage of *Z. marina* may in fact be the newly described *Z. pacifica*. The presence of a cryptic species of *Zostera* in the California Channel Islands was proposed following an extensive genetic survey of the California Bight based on nine microsatellite loci (Coyer et al. 2008 and review therein). *Zostera pacifica* (wide-leaved eelgrass) was found to be restricted to the California Channel Islands and the adjacent mainland and parts of South San Diego Bay. The problem is that Z. *pacifica* is typically wide-leafed but *Z. marina* can also be wide-leafed, rendering this morphological trait unreliable. Rather, the only conclusive diagnostic feature is molecular and entails the failure of microsatellite locus CT-20 (Reusch 2000) to amplify in *Z. pacifica* (Coyer et al. 2008). A STRUCTURE analysis excluding locus CT-20 further revealed varying degrees of introgression between the two species at three locations. Coyer et al. (2008) concluded that the distribution of *Z. pacifica* follows the glacial age land mass of the California Bight. The recognition of two species is important in terms of protection of biodiversity, transplant mitigation and management, which has been extensive in southern California (Coyer et al. 2008, Olsen et al. 2014).

The 12 San Diego (SD) samples used in the present study amplified successfully with the CT-20 microsatellite locus, thus designating them as *Z. marina*. However, our resequencing data from these samples reveals the split between SD and BB (Fig. 2G), as well as introgression of WAS and BB with SD (Fig. 2D) in a phylogeographically shallow timeframe. This begs the age-old question of how much divergence is needed to recognize a species vs. a subspecies, variety or race? At present, we continue to designate these samples as intra-specific admixtures of *Z. marina,* but noting that recognition of an inter-specific admixture would not affect any of our conclusions in this paper. What will be interesting are the potential differences in functional gene traits that population genomics makes possible. With this in mind, a second, high-quality reference genome of a non-admixed East Pacific *Z. marina* is currently being sequenced as part of our characterization of the *Z. marina* pangenome; a companion to the currently available, chromosomal-level reference genome originating in the North-East Atlantic (Baltic Sea; Olsen et al. 2016; Ma et al. 2021).

Coyer, J. A., Miller, K. A., Engle, J. M., Veldsink, J., Cabello-Pasini, A., Stam, W. T. & Olsen, J. L. (2008). Eelgrass meadows in the California Channel Islands and adjacent coast reveal a mosaic of two species, evidence for introgression and variable clonality. *Annals of Botany, 101*(1), 73-87. doi:10.1093/aob/mcm288

Ma, X., Olsen, J. L., Reusch, T. B., Procaccini, G., Kudrna, D., Williams, M., . . . Van de Peer, Y. (2021). Improved chromosome-level genome assembly and annotation of the seagrass, *Zostera marina* (eelgrass). *F1000Research, 10*(289), 289. doi:10.12688/f1000research.38156.1

Olsen, J. L., Coyer, J. A. & Chesney, B. (2014). Numerous mitigation transplants of the eelgrass *Zostera marina* in southern California shuffle genetic diversity and may promote hybridization with *Zostera pacifica*. *Biological Conservation, 176*, 133-143. doi:10.1016/j.biocon.2014.05.001

Reusch, T. B. H. (2000). Five microsatellite loci in eelgrass *Zostera marina* and a test of cross-species amplification in *Z. noltii* and *Z. japonica*. *Molecular Ecology, 9*(3), 371-373. doi:10.1046/j.1365-294x.2000.00874-4.x

Supplementary Tables

Supplementary Table 1: Population metadata for 16 worldwide *Zostera marina* populations

	Region	Population code	Population location	Latitude	Longitude	*ZEN Cross ref	Sample Numbering
West Pacific							
	Japan North	JN	Akkeshi-ko Estuary, Hokkaido, Japan	43.021N	144.903E	JN-A	JN101–JN112
	Japan South	JS	Ikunoshima, Japan (Inland sea)	34.298N	132.916E	JS-A	JS201–JS 212
East Pacific							
	Bering Sea Alaska Coastal Current **LME	ASL	Safety Lagoon /Safety Sound, Alaska, USA	64.485N	164.762W	Non-ZEN	ASL1–ASL12
	Gulf of Alaska Alaska Coastal Current LME	ALI	Izembek Lagoon, Alaska, USA	55.329N	162.821W	Non-ZEN	ALI301–ALI312
	California Current LME	WAS	Willapa Bay, Washington State, USA	46.474N	124.028W	WAS-A	WAS401–WAS412
	California Current LME	BB	Westside Park, Bodega Bay, California, USA	38.320N	123.055W	BB-A	BB501–BB512
	California Current LME	SD	Shelter Island, San Diego Bay, California, USA	32.714N	117.225W	SD-A	SD601–SD612
West Atlantic							
	Quebec	QU	Pointe-Lebel, Quebec, Canada	49.112N	68.176W	QU-A	QU701–QU711
	Massachusetts	MA	Dorothy Cove, Massachusetts, USA	42.420N	70.915W	MA-A	MA801–MA812
	North Carolina	NC	Middle Marsh, North Carolina, USA	34.692N	76.623W	NC-A	NC 1103–NC 1112
East Atlantic							
	Northern Norway	NN	Røvika, Norway (near Bødo)	67.268N	15.257E	NN-B	NN1201–NN1212
	Sweden	SW	Torserød, Sweden (west coast)	58.313N	11.549E	SW-A	SW1401–SW1412
	Wales	WN	Port Dinllaen, Wales, UK	52.991N	4.450W	WN-A	WN1501–WN1512
	Portugal	PO	Culatatra, Ria Formosa, S. Portugal	37.040N	7.910W	PO-A	PO1601–PO1611
Mediterranean Sea							
	France	FR	Bouzigues, Thau Lagoon, France	43.447N	3.662E	FR-A	FR1701–FR1712
	Croatia	CZ	Adriatic Sea, Posedarje, Croatia	44.212N	15.491E	CR-A	CZ1801–CZ1812

*The *Zostera* Experimental Network (ZEN) has produced extensive ecological and physical metadata for 50 populations of which the above represent 16 of them. All data used in their analyses, and associated R code, are available at https://doi.org/10.5281/zenodo.6808753 with the exception of the genetic data, available at https://doi.org/10.5281/zenodo.3660013 . **LME = large marine ecosystem

Supplementary Table 2: Possible parent-descendant pairs under self-fertilization as detected using the shared heterozygosity index.

Selfing Pair	Parent Ramet	Descendant Ramet
SP_01	NN05	NN02
SP_02	NN05	NN06
SP_03	NN05	NN07
SP_04	NN05	NN09
SP_05	NN05	NN10
SP_06	NN08	NN02
SP_07	NN08	NN06
SP_08	NN08	NN07
SP_09	NN08	NN09
SP_10	NN08	NN10
SP_11	SD03	SD02
SP_12	SD03	SD12
SP_13	SD12	SD02
SP_14	WN02	WN03
SP_15	WN02	WN07
SP_16	WN12	WN05

See Supplementary Fig. 3 for further details on the shared heterozygosity index.

Supplementary Table 3: Clone mates detected based on the shared heterozygosity index.

Genet	Clonemates						
Genet_01	BB04	BB05					
Genet_02	BB09	BB10					
Genet_03	JS03	JS04					
Genet_04	NN02	NN06	NN07	NN09	NN10		
Genet_05	PO02	PO05	PO07	PO08	PO10	PO11	PO12
Genet_06	PO03	PO04	PO06	PO09			
Genet_07	SD04	SD11					
Genet_08	SD06	SD09					
Genet_09	WN04	WN09					
Genet_10	WN06	WN10					
Genet_11	NN05	NN08					

See Supplementary Fig. 3 for further details on the shared heterozygosity index.

Supplementary Table 4: Statistical comparison of genetic diversity measures

Genetic diversity measures (π, H_{OBS}) were subject to 1-way analysis of variance. As populations were significantly different for both variables (π: $F_{15,80}$=320, P<0.0001; H_{OBS}: $F_{15,137}$=285, P<0.0001), this was followed by a Tukey Cramer HSD post-hoc test, using a family-wise error rate of α=0.05. Different letters indicate statistically different mean values. Population abbreviations are given in Fig. 1. Source Data are given in the Source Data Fig. 1.

Population		π	Population		H_{OBS}
JS	A	0.0816	JS	A	0.0984
JN	B	0.0695	JN	B	0.0852
BB	C	0.0584	BB	B	0.0798
WAS	D	0.0450	WAS	C	0.0596
SD	E	0.0302	SD	D	0.0421
NC	F	0.0148	ASL	E	0.0210
ALI	F G	0.0133	ALI	E F	0.0203
ASL	F G H	0.0116	NC	E F	0.0152
PO	F G H	0.0113	PO	E F G	0.0123
CZ	F G H I	0.0096	FR	F G	0.0116
FR	F G H I	0.0094	CZ	F G	0.0107
MA	F G H I	0.0078	MA	F G	0.0090
SW	G H I	0.0064	SW	F G	0.0090
WN	H I	0.0053	WN	F G	0.0088
QU	H I	0.0047	QU	G	0.0057
NN	I	0.0025	NN	G	0.0028

Supplementary Table 5: Matrix of F_{ST}-values among population pairs

**Pop\Pop**	**JN**	**JS**	**ASL**	**ALI**	**WAS**	**BB**	**SD**	QU	MA	NC	NN	SW	WN	PO	FR	CZ
**JN**	0.0	0.523	0.399	0.31	0.474	0.525	0.692	0.629	0.635	0.616	0.58	0.634	0.587	0.518	0.64	0.633
**JS**		0.0	0.698	0.618	0.649	0.641	0.724	0.731	0.738	0.723	0.69	0.74	0.697	0.628	0.739	0.736
**ASL**			0.0	0.503	0.613	0.686	0.881	0.898	0.885	0.851	0.897	0.89	0.889	0.878	0.881	0.877
**ALI**				0.0	0.498	0.59	0.852	0.916	0.896	0.845	0.923	0.904	0.908	0.876	0.887	0.882
**WAS**					0.0	0.389	0.704	0.782	0.783	0.761	0.753	0.785	0.755	0.708	0.783	0.779
**BB**						0.0	0.556	0.782	0.784	0.764	0.748	0.787	0.752	0.69	0.784	0.78
**SD**							0.0	0.909	0.904	0.881	0.897	0.908	0.895	0.857	0.9	0.898
QU								0.0	0.391	0.358	0.594	0.445	0.473	0.738	0.609	0.57
MA									0.0	0.229	0.457	0.328	0.345	0.644	0.539	0.498
NC										0.0	0.345	0.277	0.28	0.502	0.464	0.427
NN											0.0	0.26	0.401	0.792	0.587	0.545
SW												0.0	0.175	0.658	0.517	0.455
WN													0.0	0.704	0.54	0.495
PO														0.0	0.601	0.609
FR															0.0	0.503
CZ																0.0

For population abbreviations see Fig. 1. Values are calculated based on the core SNP set (11,705 SNP, Supplementary Fig. 1). Pacific populations are shown in boldface. All pairwise F_{ST} have significant p values ($p < 0.001$), based on 1,000 permutation runs, hence only the half diagonal matrix is depicted. Note that JN shows the smallest F_{ST} of all Pacific populations with any other population from the Atlantic side (Atlantic + Mediterranean Sea).

Supplementary Table 6: *Zostera marina*-associated fauna among Pacific and Atlantic locations

Greater numbers of species are found to be intimately associated with *Zostera marina* in the Pacific than in the Atlantic. Entries shaded in gray are from the Pacific, those in white are Atlantic. (A) are specialist feeders on *Z. marina*; (B) facultative feeders on green plant tissue; and (C) habitat specialists. Species found to consume only senescent/ detrital tissues were excluded but reported in (D); stabile isotopic evidence alone was not sufficient to verify consumption of green tissue.

The lists of *Zostera*-associated fauna were derived from standardized surveys of epifaunal crustaceans and gastropods (and also from the literature) at 20 eelgrass beds in the Pacific Ocean and 28 in the Atlantic Ocean (including three from the Mediterranean Sea). The field work was conducted in 2014 as part of the Zostera Experimental Network (ZEN) http://zenscience.org/ . See Duffy et al. (2022) and Gross et all (2022).

In total, these surveys found 55 peracarid crustacean species from Atlantic sites and 60 species from Pacific sites (Gross et al. 2022). There were 52 species of gastropods in the Atlantic eelgrass beds but only 31 in the Pacific; thus, the increased number of taxa feeding on green tissue and habitat specialists in the Pacific are not the result of overall higher diversity of eelgrass-associated taxa there.

**Species**	**Group**	**Coastline** (A= Atlantic, P= Pacific, W= Western, E= Eastern)	**Reference**
**A. Feeding Specialist on *Zostera* /high proportion of diet**			
Tectura depicta	gastropod	EP	7, 14
Elysia catulus[a]	gastropod	WA	13
Lottia alveus[b]	gastropod	WP, EP, WA (extinct)	3
Siphonacmea oblongata	gastropod	WP	23
**B. Facultative / readily consume live green *Zostera* tissue**			
Ampithoe lacertosa	amphipod	WP, EP	7, 20, 22
Ampithoe dalli	amphipod	EP	7, 20, 22
Ampithoe valida[c]	amphipod	WP, EP, WA (introduced)	7, 20, 22
Ampithoe sectimanus	amphipod	WP, EP	7, 20, 22
Pentidotea resecata	isopod	EP	7, 25
Idotea balthica	isopod	WA, EA	1, 20
Erichsonella crenulata	isopod	EP	16
Erichsonella attenuata	isopod	WA	2. 7
Idotea chelipes	isopod	EA	7, 18
Idotea ochotensis	isopod	WP	7, 22
Lacuna decorata	gastropod	WP	22
Zeuxo normani	tanaid	WP EP	16
**C. Habitat specialist on *Zostera* exhibiting**			
Kushia zosteraphila	copepod	WP	8
Phyllaplysia taylori	gastropod	EP	9
Siphonacmea oblongata	gastropod	WP	23
Syngnathus leptorhynchus	fish	EP	6

Erichsonella crenulata	gastropod	EP	14
Sagartiogeton viduatus	anemone	EA	
**D. Consumes eelgrass detritus/ senescent blades / no clear evidence for live eelgrass, but not green tissue (incomplete listing)**			
Erichsonella filiformis	isopod	WA	19
Ampithoe longimana	amphipod	WA	4
Cymadusa compta	amphipod	WA	4
Carcinus maenas[d]	decapod	EA (global invader)	15
Idotea phosphorea	isopod	WA	21
Idotea fewkesii[e]	isopod	EP	7
Idotea rufescens[e]	isopod	EP	7
Idotea schmitti[e]	isopod	EP	7

Footnotes to species column:

(a) Jensen (1982, 1983) considers *E. catulus* as an ecotype or subspecies called *E. serca* that has a distribution extedning further south than the distribution of eelgrass on the western North Atlantic coast.

(b) Limpet species that went extinct during wasting disease epidemic in 1930s in Washington State, but is still present in NEP and NWP (Carlton et al. 1991)

(c) *Ampithoe valida* originiated in the Pacific and invaded the Atlantic ~3 mya. Atlantic populations do not appear to feed regularly on eelgrass, but some Pacific ones do (Sotka et al. 2017).

(d) some references suggest that juvenile green crabs feed in the meristematic tissue after clipping shoots at their base, but this species is otherwise widely known to be a predator on bivalves and other infaunal and epifaunal invertebrates.

(e) *Idotea*/ *Pentidotea* spp. include several uncommon species that are uncertain as to taxonomic validity: *P. schmitti, I. rufescens, I. fewkesii*; diets are also uncertain. Most appear to be found in laminarian zone of rocky shores yet these were all collected from *Z. marina* beds in Oregon and Washington State.

Bell, T. M. & Sotka, E. E. (2012). Local adaptation in adult feeding preference and juvenile performance in the generalist herbivore *Idotea balthica*. *Oecologia, 170*, 383-393. doi:10.1007/s00442-012-2302-3 **ref1**

Boström, C. & Mattila, J. (2005). Effects of isopod grazing: an experimental comparison in temperate (*Idotea balthica*, Baltic Sea, Finland) and subtropical (*Erichsonella attenuata*, Gulf of Mexico, USA) ecosystems. *Crustaceana, 78*(2), 185-200. **ref2**

Carlton, J. T., Vermeij, G. J., Lindberg, D. R., Carlton, D. A. & Dubley, E. C. (1991). The first historical extinction of a marine invertebrate in an ocean basin: the demise of the eelgrass limpet *Lottia alveus*. *The Biological Bulletin, 180*(1), 72-80. **ref3**

Clark, K. B. (1975). Nudibranch life cycles in the Northwest Atlantic and their relationship to the ecology of fouling communities. *Helgoländer Wissenschaftliche Meeresuntersuchungen, 27*(1), 28-69. doi:10.1007/BF01611686

Duffy, J. E. & Harvilicz, A. M. (2001). Species-specific impacts of grazing amphipods in an eelgrass-bed community. *Marine Ecology Progress Series, 223*, 201-211. doi:10.3354/meps223201. **ref4**

Duffy, J. E., Stachowicz, J. J., Reynolds, P. L., Hovel, K. A., Jahnke, M., Sotka, E. E., . . . Olsen, J. L. (2022). A Pleistocene legacy structures variation in modern seagrass ecosystems. *Proceedings of the National Academy of Sciences, 119*(32), e2121425119. doi:10.1073/pnas.2121425119. **ref5**

Fishbase. https://www.fishbase.se/summary/Syngnathus-leptorhynchus.html **ref6**

Gross, C. P., Duffy, J. E., Hovel, K. A., Kardish, M. R., Reynolds, P. L., Boström, C., . . . Stachowicz, J. J. (2022). The biogeography of community assembly: latitude and predation drive variation in community trait distribution in a guild of epifaunal crustaceans. *Proceedings of the Royal Society B, 289*(1969), 20211762. doi:10.1098/rspb.2021.1762 **ref7**

Harris, V. A. (1996). Two new genera belonging to the family Porcellidiidae (Crustacea, Copepoda, Harpacticoida) from Iwate Prefecture, Japan. *Bulletin of the National Science Museum. Series A, Zoology/National Science Museum, 22*(4), 199-218. **ref8**

Hughes, A. R., Best, R. J. & Stachowicz, J. J. (2010). Genotypic diversity and grazer identity interactively influence seagrass and grazer biomass. *Marine Ecology Progress Series, 403*, 43-51. doi:10.3354/meps08506. **ref9**

Jensen, K. R. (1982). Occurrence of *Elysia serca* Marcus in Florida, with notes on the synonymy and biology of the species. *Journal of Conchology*, 31, 87–94. **ref10**

Jensen, K. R. (1983). Further Notes on the Ecology and Systematics of *Elysia serca* Marcus (Opisthobranchia, Ascoglossa). *Journal of Molluscan Studies*, 49 (12A), 69–72. **ref11**

Kajihara, R., Komorita, T., Hamada, A., Shibanuma, S., Yamada, T. & Montani, S. (2010). Possibility of direct utilization of seagrass and algae as main food resources by small gastropod, *Lacuna decorata*, in a subarctic lagoon, Hichirippu,

eastern Hokkaido, Japan with stable isotope evidences of carbon and nitrogen. *Plankton and Benthos Research, 5*(3), 90-97. doi:10.3800/pbr.5.90. **ref12**

Lark, K.B. (1975). Nudibranch life-cycles in the Northwestern Atlantic and their rrelationship to the ecology of fouling commuities. *Helgoländer Wissenschaftliche Meeresuntersuchungen*, 27, 28-69 **ref13**

Lewis, L. S. & Anderson, T. W. (2012). Top-down control of epifauna by fishes enhances seagrass production. *Ecology, 93*(12), 2746-2757. doi:10.1890/12-0038.1 **ref14**

Malyshev, A. & Quijón, P. A. (2011). Disruption of essential habitat by a coastal invader: new evidence of the effects of green crabs on eelgrass beds. *ICES Journal of Marine Science, 68*(9), 1852-1856. doi:10.1093/icesjms/fsr126 **ref15**

Menzies, R. J. (1950). A remarkable new species of marine isopod *Erichsonella crenulata* n sp, from Newport Bay, California. *Bull. South. Cal. Acad. Sci, 49*(1), 29-35. **ref16**

Nakaoka, M. (2002). Predation on seeds of seagrasses *Zostera marina* and *Zostera caulescens* by a tanaid crustacean *Zeuxo* sp. *Aquatic Botany, 72*(2), 99-106. doi:10.1016/S0304-3770(01)00213-3 **ref17**

Nienhuis, P. H. & Groenendijk, A. M. (1986). Consumption of eelgrass (*Zostera marina*) by birds and invertebrates: an annual budget. *Marine Ecology Progress Series, 29*, 29-35. **ref18**

Olsen, Y. S., Fox, S. E., Teichberg, M., Otter, M. & Valiela, I. (2011). δ15N and δ13C reveal differences in carbon flow through estuarine benthic food webs in response to the relative availability of macroalgae and eelgrass. *Marine Ecology Progress Series, 421*, 83-96. doi:10.3354/meps08900. **ref19**

Poore, A. G. B., Ahyong, S. T., Lowry, J. K. & Sotka, E. E. (2017). Plant feeding promotes diversification in the Crustacea. *Proceedings of the National Academy of Sciences, 114*(33), 8829-8834. doi:10.1073/pnas.1706399114. **ref20**

Robertson, A. I. & Mann, K. H. (1980). The role of isopods and amphipods in the initial fragmentation of eelgrass detritus in Nova Scotia, Canada. *Marine Biology, 59*, 63-69. doi:10.1007/BF00396983. **ref21**

Sotka, E. E., Bell, T., Hughes, L. E., Lowry, J. K. & Poore, A. G. B. (2017). A molecular phylogeny of marine amphipods in the herbivorous family Ampithoidae. *Zoologica Scripta, 46*(1), 85-95. doi:10.1111/zsc.12190 **ref22**

Suzuki, M., Watanabe, K. & Mukai, H. (2002). Feeding Habits and Growth of an Isopod, *Idotea ochotensis* Brandt, in Akkeshi Bay, Hokkaido, Northern Japan. *Japanese Journal of Benthology, 57*, 13-20. **ref23**

Toyohara, T., Nakaoka, M. & Tsuchida, E. (2001). Population dynamics and life history traits of *Siphonacmea oblongata* (Yokoyama) on seagrass leaves in Otsuchi Bay, north-eastern Japan (Siphonariidae, Pulmonata). *Venus (Journal of the Malacological Society of Japan), 60*(1-2), 27-36. doi:10.18941/venus.60.1-2_27 **ref24**

Welton, L. L. & Miller, M. A. (1980). Isopoda and Tanaidacea: the isopods and allies. In R. H. Morris, D. P. Abbott, & E. C. Haderlie (Eds.), *Intertidal invertebrates of California* (pp. 536-558). California: Stanford University Press. **ref25**

Supplementary Figures

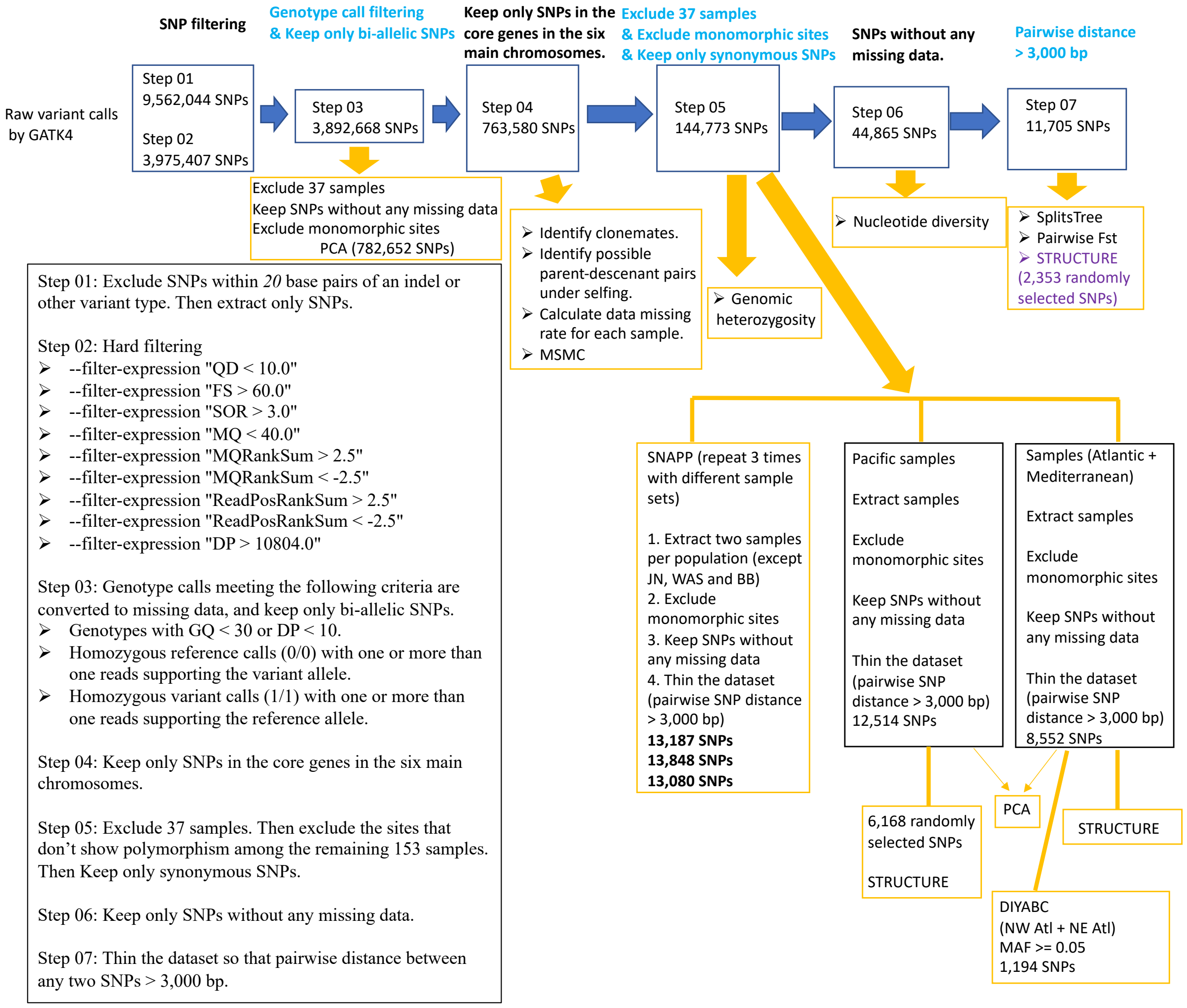

Supplementary Fig. 1: Workflow for SNP calling and filtering.

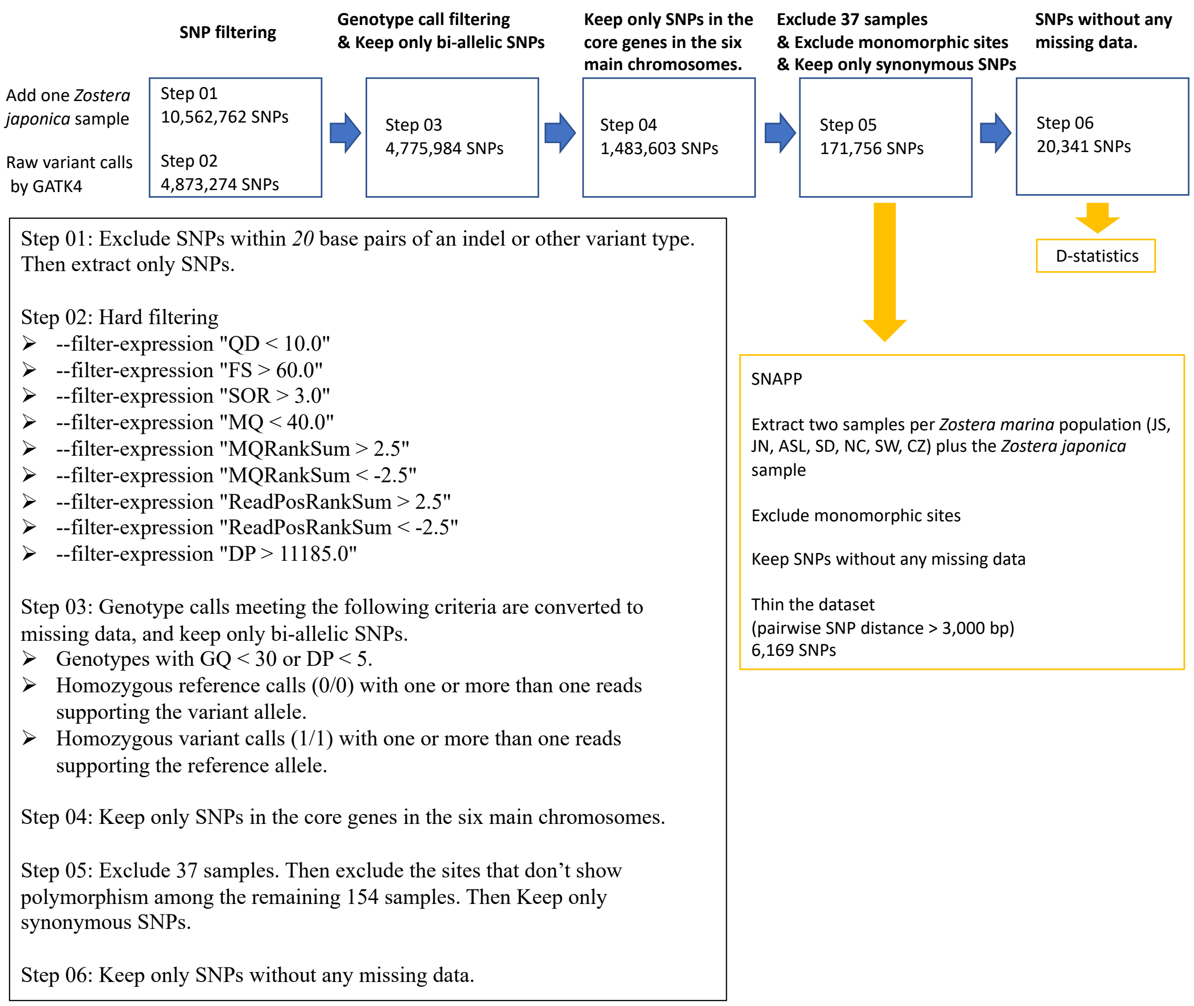

Supplementary Fig. 2: Workflow for SNP calling and filtering that required an outgroup.

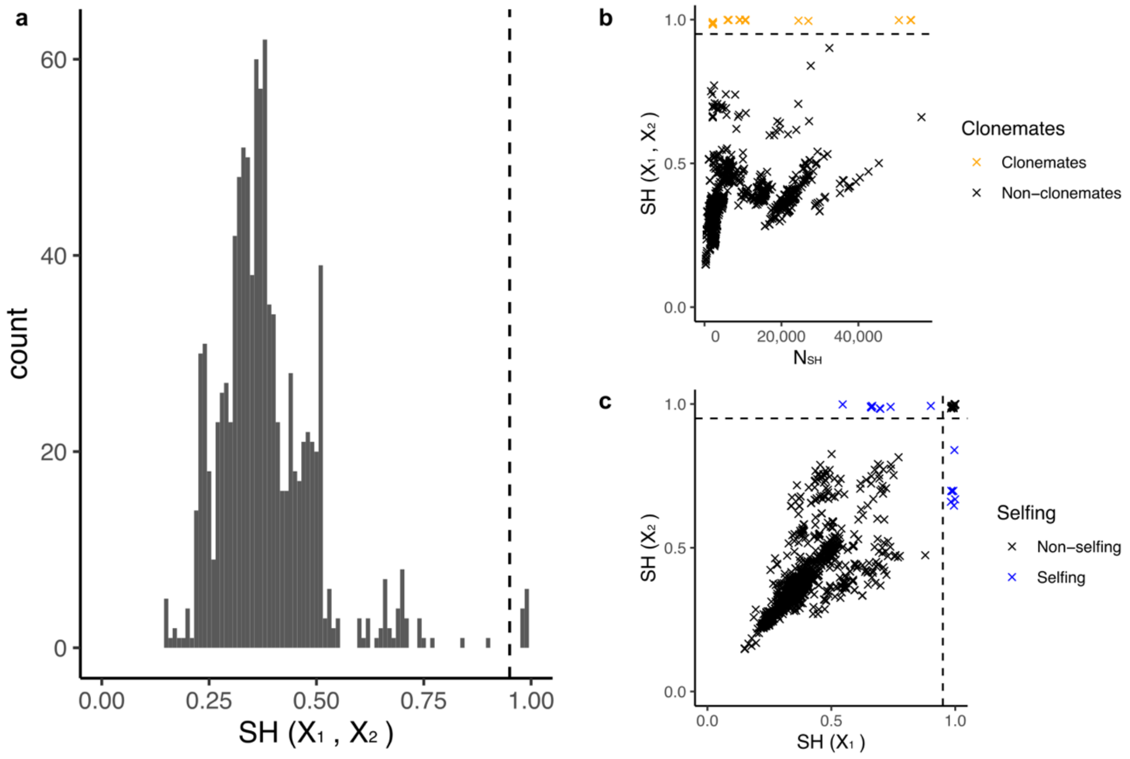

Supplementary Fig. 3: Detecting clone mates and possible parent-descendant pairs under selfing based on shared heterozygosity (SH)

Analysis were performed according to Yu et al. (2022) **a,** Histogram of pairwise $SH(X_1, X_2)$ for each pair of ramets within the same population. The threshold is set to 0.95, indicated by the vertical dashed line. **b,** Identification of pairs of ramets belonging to the same genet (=clone mates) based on $SH(X_1, X_2)$. Clone mate pairs show $SH(X_1, X_2) > 0.95$. **c,** Identification of possible parent-descendant pairs under selfing based on $SH(X_1)$ and $SH(X_2)$. Ramets originating via selfing inherit a subset of the heterozygous loci from the parent.

Yu L., Stachowicz J.J., DuBois K. & Reusch T.B.H. (2023) Detecting clonemate pairs in multicellular diploid clonal species based on a shared heterozygosity index. *Molecular Ecology Resources*, 23, 592-600.

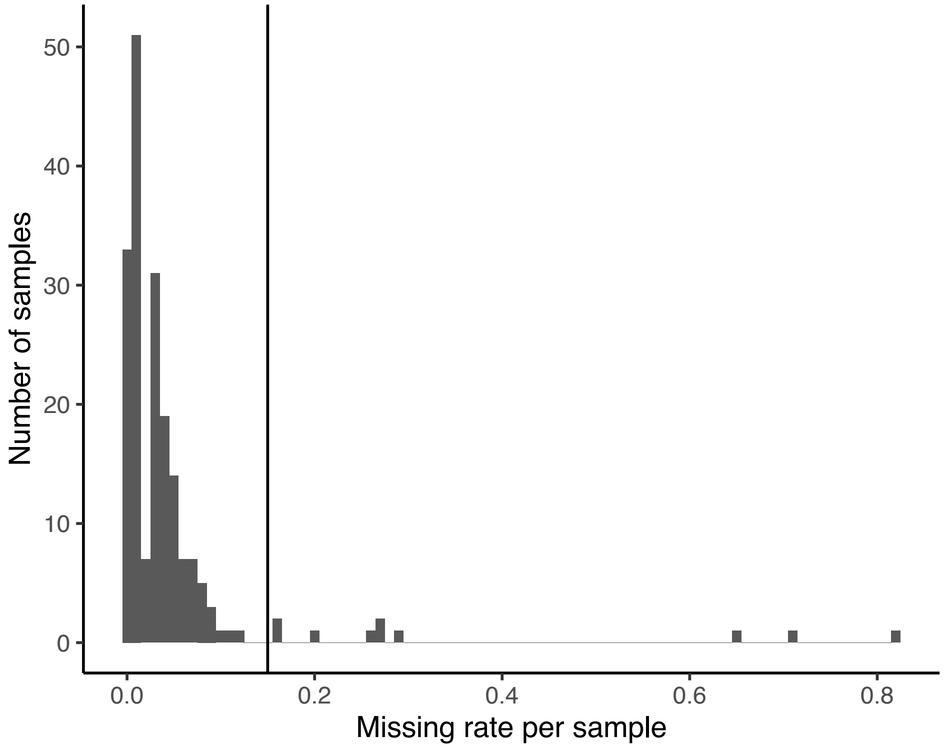

Supplementary Fig. 4: Missing data distribution for each sample.

The data missing rate for each sample was calculated based on SNP data set ZM_HQ_SNPs (763,580 SNPs). Missing rate=number of loci with missing data/763,580. Samples with <15% missing data were retained. Ten of the 190 samples had a missing rate >15% (ALI01, ALI02, ALI03, ALI04, ALI05, ALI06, ALI10, ALI16, QU03 and SD08) and were excluded from the data set.

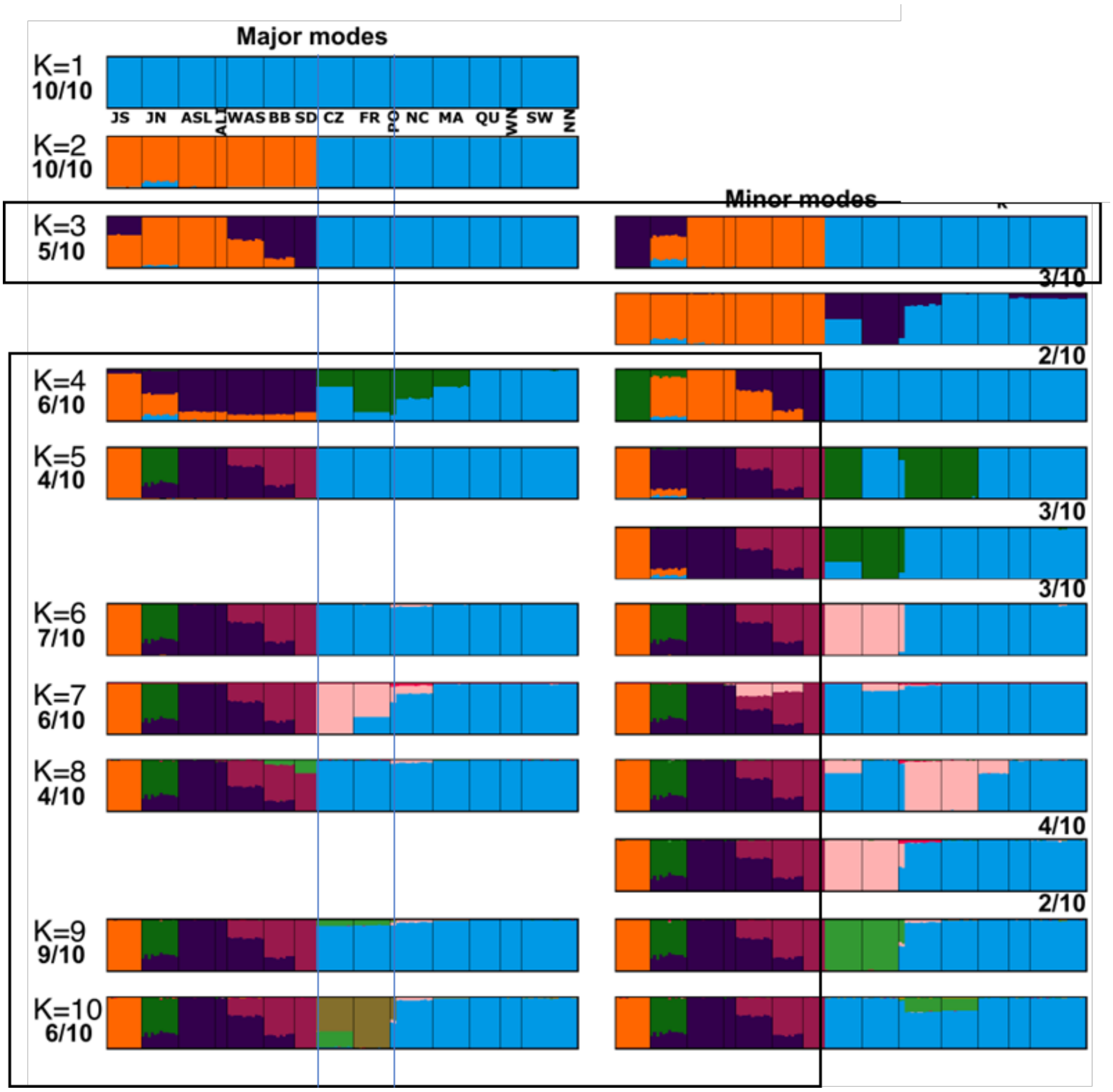

Supplementary Fig. 5: Global STRUCTURE results for K from 1 to 10.

The analysis was repeated 10 times. Numbers indicate how many times the mode occurred among the 10 runs. While K=2 is used in Fig. 2 as the basic Pacific-Atlantic split, K=3 (major mode) picks up the link between JS and WAS/BB/SD populations with the California Coastal Current, and the JN/ASL/ALI populations following the Alaska coastal current from the NPC gateway. At K=4 and above, relationships among the Pacific populations remain stable; the Mediterranean and Atlantic remain unstable as mirrored in the PCA analyses for each ocean.

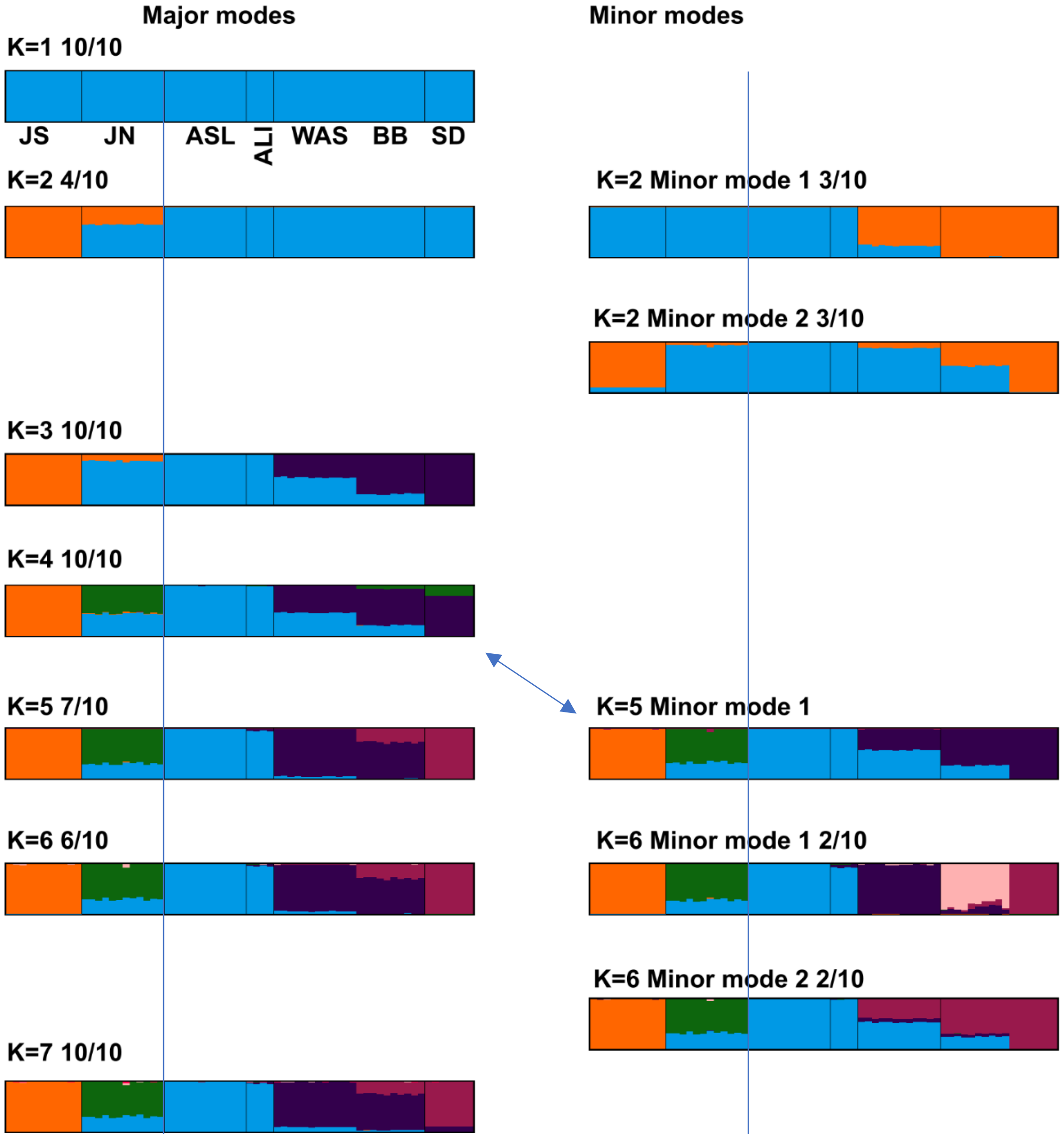

Supplementary Fig. 6: STRUCTURE results for Pacific populations only for K from 1 to 7.

The analysis was repeated 10 times with the same parameters. The number indicates how many times the mode occurred among runs. Vertical lines separate the West and East Pacific. While K=3 is shown in Fig. 4, K=4 picks up the connection among JN and SD, while K=5-7 (major and minor modes) consistently pick up the admixture among SD, BB and WAS. Clusters up to K=5 are supported by the parameter medmeak and maxmeak (Puechmaille 2016).

Puechmaille, S.J. (2016) The program structure does not reliably recover the correct population structure when sampling is uneven: subsampling and new estimators alleviate the problem. *Molecular Ecology Resources,* 16, 608-627 doi https://doi.org/10.1111/1755-0998.12512

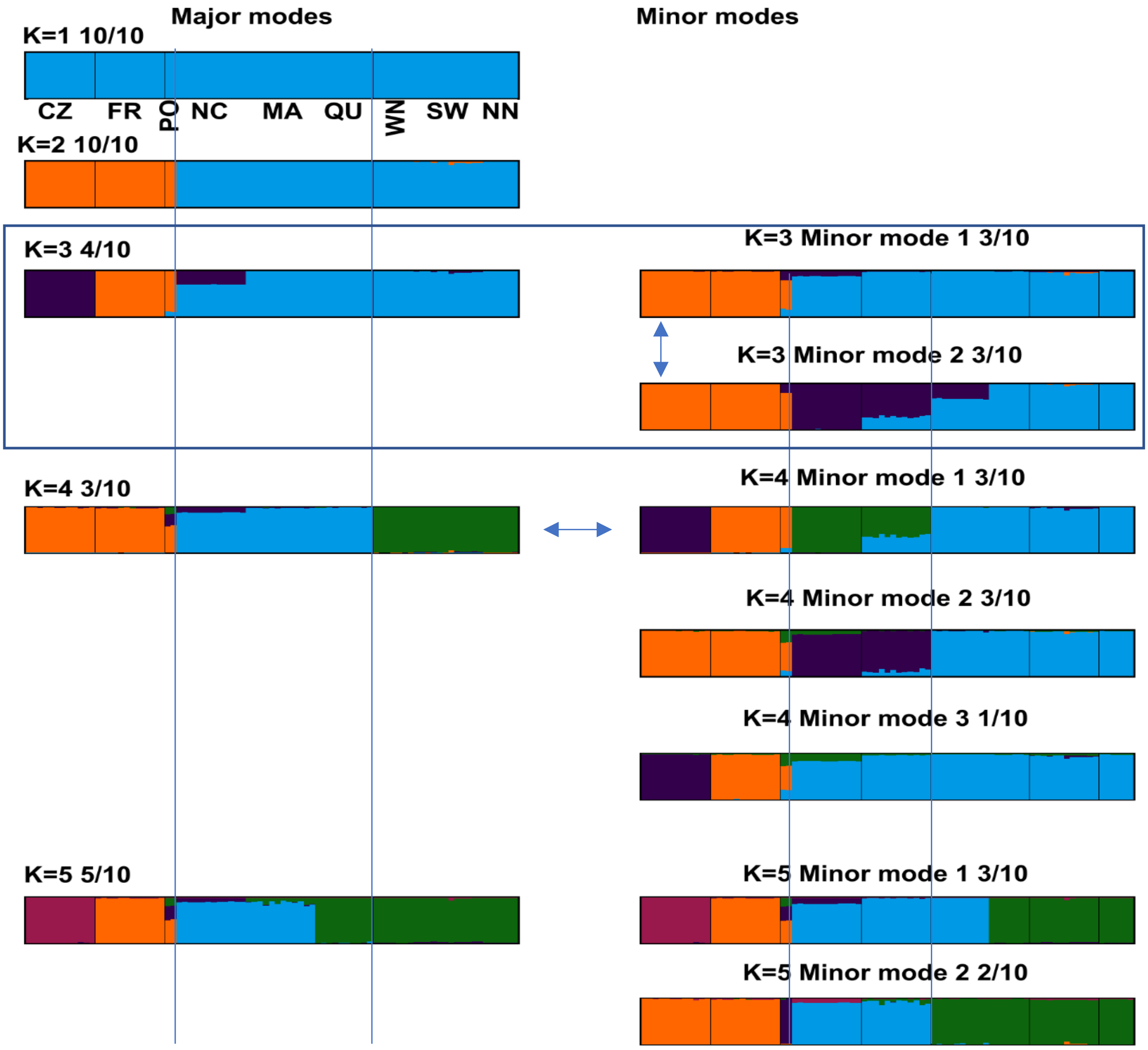

Supplementary Fig. 7: STRUCTURE results for Atlantic populations for K from 1 to 5.

The analysis was repeated 10 times. Numbers indicate how many times the mode occurred among runs. Vertical lines separate the Mediterranean, and the West and West Atlantic populations. As the minimal likely no of clusters, K=2 is used in main Fig. 4 in which the Mediterranean (including Portugal) and Atlantic are easily separated, but the West and East Atlantic not. When exploring K=3 (major and minor modes) the separation among NC and the Mediterranean becomes visible. The two minor modes also place Portugal with the Mediterranean and NC. At K=4, there is instability within each of the major partitions. Along with the tightness of points in the Atlantic PCA (Fig. 2a), this supports the lack of population level differentiation. Clusters up to K=4 are supported by medmeak and maxmeak (Puechmaille 2016).

Puechmaille, S.J. (2016) The program structure does not reliably recover the correct population structure when sampling is uneven: subsampling and new estimators alleviate the problem. *Molecular Ecology Resources,* 16, 608-627 doi https://doi.org/10.1111/1755-0998.12512

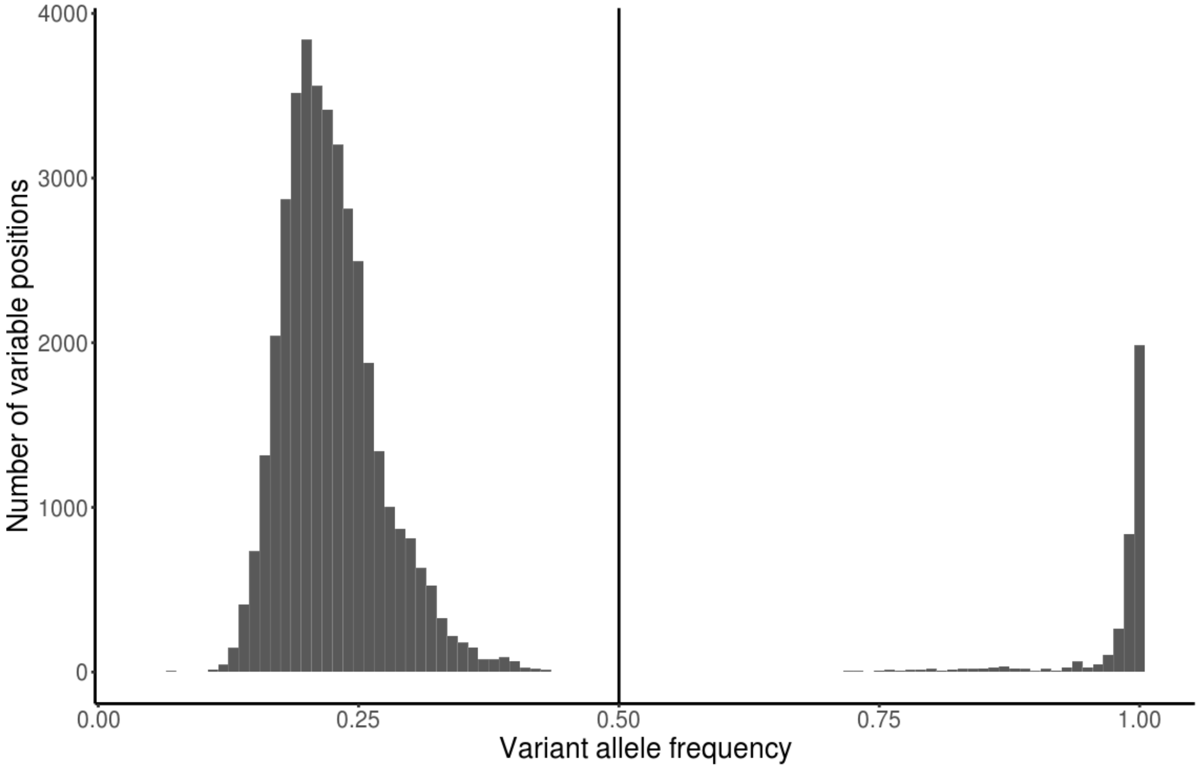

Supplementary Fig. 8: Distribution of variant allele frequencies based on 163 chloroplast genomes

Variant allele frequency diagram indicating the number of reads supporting the variant allele aligned to a given locus. These reads might originate either from the chloroplast DNA or from mitochondrial/nuclear copies of specific chloroplast regions (mtptDNA/NUPTs). Therefore, genuine fixed mutations in chloroplasts can be represented by an allele frequency lower than one. We observe two clearly separated peaks of allele frequency due to the significant prevalence of chloroplast DNA. The left peak is likely to represent loci mutated in mitochondrial (or nuclear) copies of the specific chloroplast regions, but not on the chloroplasts themselves. The right peak is likely to represent genuine chloroplast-fixed mutations. We only focused on the chloroplast fixed variant alleles by setting a threshold of >0.5.

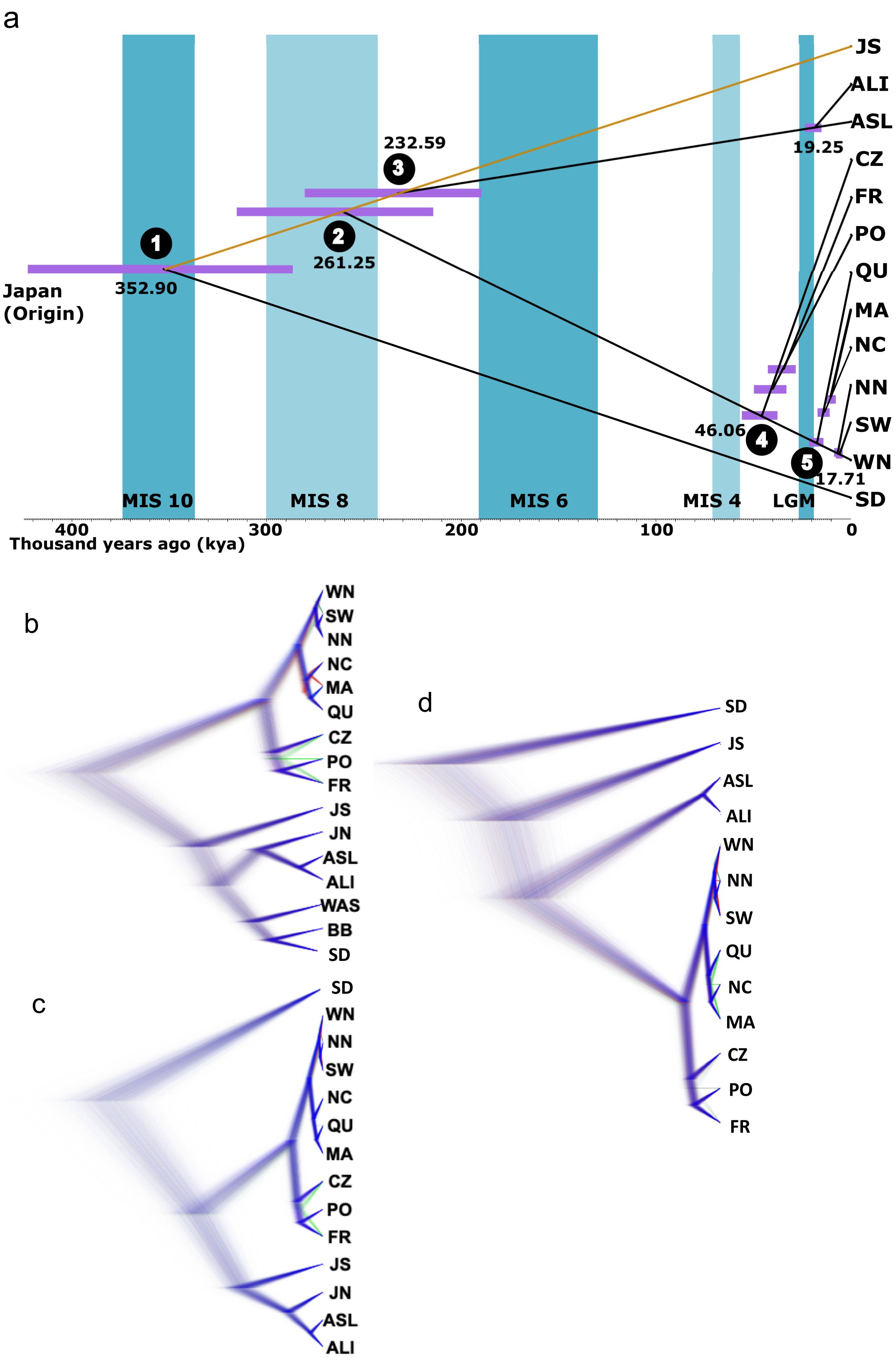
a
JS
ALI
ASL
CZ
FR
PO
QU
MA
NC
NN
SW
WN
SD
232.59
3
19.25
1
2
261.25
Japan
(Origin)
352.90
46.06
4
5
17.71
MIS 10
MIS 8
MIS 6
MIS 4
LGM
400
300
200
100
0
Thousand years ago (kya)
b
WN
SW
NN
NC
MA
QU
CZ
PO
FR
JS
JN
ASL
ALI
WAS
BB
SD
c
SD
WN
NN
SW
NC
QU
MA
CZ
PO
FR
JS
JN
ASL
ALI
d
SD
JS
ASL
ALI
WN
NN
SW
QU
NC
MA
CZ
PO
FR

Supplementary Fig. 9: Alternative SNAPP topologies when admixed populations are included.

a. Tree topology based on SNAPP analysis in Newick format using a reduced data set of 13 populations in which admixed populations (JN, WAS, BB) were excluded, as in the main manuscript (Fig. 4). In some of the replicate SNAPP runs, a divergence between the Pacific lineage and the Atlantic ("2") occurred before a second dispersal event to the NE-Pacific ("3"). To explain this, three trans-Pacific colonization events would have to be invoked, as well as an extinct "ghost" population in the Alaska region that predates the Atlantic colonization. This is why we present the more parsimonious topology in Fig. 4. Also note that Supplementary Fig. 11 (ASTRAL tree) and Supplementary Fig. 12 (StarBEAST2) support the topology shown in main Fig. 4. And finally, note that the essential divergence times of the foundation of the SD-lineage, the Atlantic populations, and the Mediterranean and East Atlantic populations are very similar here and in Fig. 4. **b-d. E**xploration of topology changes by removal of admixed populations. **b**, DensiTree cloudogram with all 16 populations (thus not accounting for admixture and creating error in the placement of SD). **c**, Cloudogram with admixed populations WAS and BB removed. **d**, definitive cloudogram as basis for Fig. 4 with WAS, BB and JN removed (i.e. 13 populations, as in panel "a").

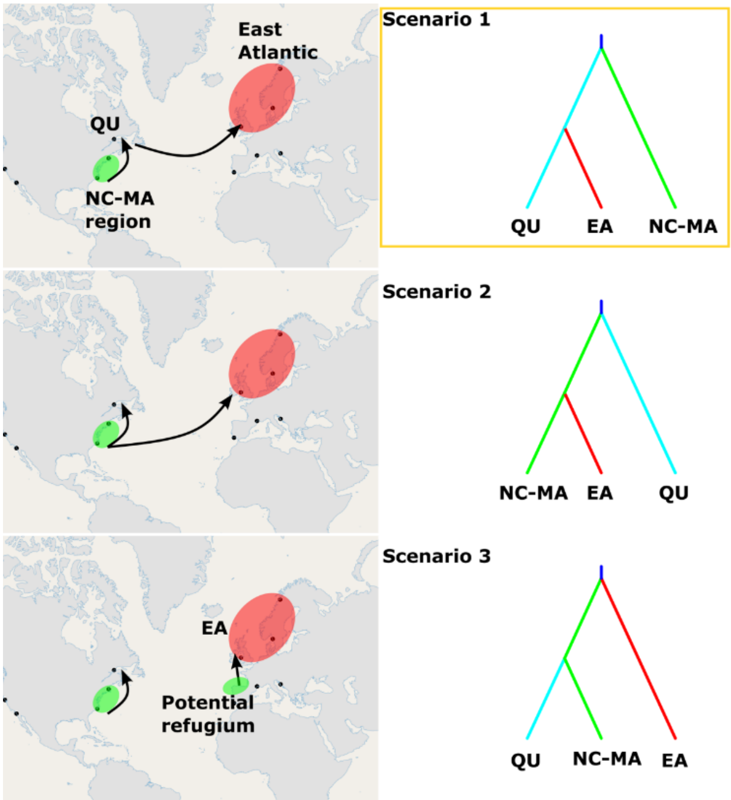

Supplementary Fig. 10: Most likely recolonization scenario of the Atlantic Ocean after the Last Glacial Maximum (LGM) using approximate Bayesian computation (ABC).

Genetic diversity (Fig 1b,c), the coalescent tree (Fig. 4) and chloroplast haplotype network (Fig. 2g) indicate that the NC-MA region was one of several west Atlantic glacial refugia during the LGM (Fig. 6b) in addition to several along the eastern Atlantic coast (Fig. 6b); the Mediterranean was a separate refugium. Both southwestern Ireland and southern Brittany are proposed as a refuge for rocky intertidal/subtidal species (Jenkins et al., 2018, Maggs et al 2008) including *Z. marina* (Becheler et al 2010). **Scenario 1**: the NC-MA region recolonized QU as stepping stone and subsequently the Northeast Atlantic. **Scenario 2**: NC-MA region recolonized QU and the Northeast Atlantic in parallel (no stepping stone). **Scenario 3**: Mainly the NC-MA region recolonized the northern NW Atlantic (QU), while a different early diverging and non-sampled refuge recolonized the Northwest Atlantic. Among the three scenarios tested, Scenario 1 is the most likely based on Approximate Bayesian computation implemented in the package DIYABC (Collin et a. 2021). Since our sampling is limited, and lacks sites that served as refugia in the Eastern Atlantic known for other coastal marine species, we cannot exclude additional such refugia.

Collin, F. D., Durif, G., Raynal, L., Lombaert, E., Gautier, M., Vitalis, R., . . . Estoup, A. (2021). Extending approximate Bayesian computation with supervised machine learning to infer demographic history from genetic polymorphisms using DIYABC Random Forest. *Molecular Ecology Resources, 21*(8), 2598-2613. doi:10.1111/1755-0998.13413

Jenkins, T. L., Castilho, R. & Stevens, J. R. (2018). Meta-analysis of northeast Atlantic marine taxa shows contrasting phylogeographic patterns following post-LGM expansions. *PeerJ, 6*, e5684. doi:10.7717/peerj.5684

Maggs, C. A., Castilho, R., Foltz, D., Henzler, C., Jolly, M. T., Kelly, J., . . . Wares, J (2008). Evaluating signatures of glacial refugia for North Atlantic benthic marine taxa. *Ecology, 89*(sp11), S108-S122. doi:10.1890/08-0257.1

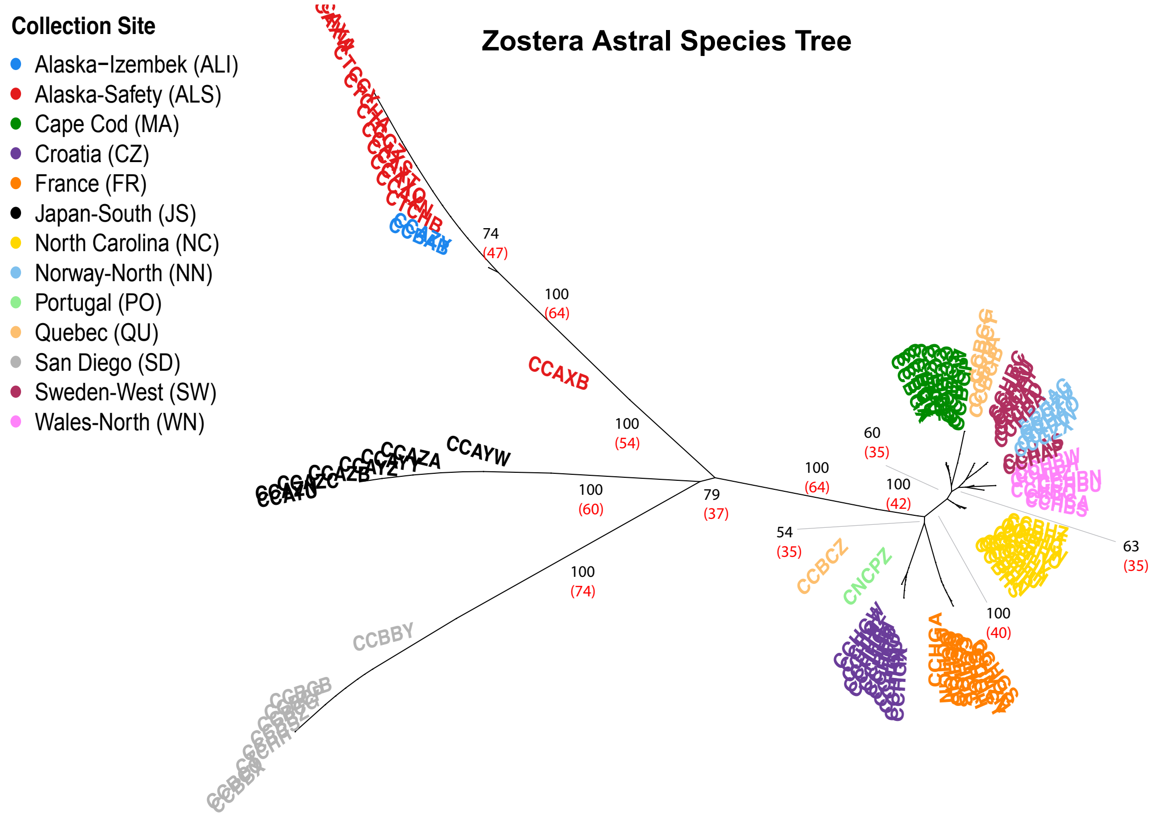

Supplementary Fig. 11: Unrooted phylogenetic tree using ASTRAL

The data set comprises the same 13 populations as in Fig. 4 and Supplementary Fig. 9a. Heavily admixed populations (BB, WAS, JN) were excluded. The final dataset for consideration included 129 samples and 20,100 aligned transcripts. Of the 18,311 core genes that were present in 97.9% of all samples, a random subset of 617 genes was selected. CDS and protein sequences from each transcript alignment were predicted using GeMoMa (v1.7). Gene trees were constructed by first aligning CDS sequences together using MAFFT (v7.475). (parameters: mafft --localpair --phylipout --maxiterate 1000), then generating individual gene trees with IQTREE (v2.1.2) (parameters -B 1000 -m K2P -T auto). With all estimated gene trees as input, the population tree was estimated with ASTRAL v5.7.3 excluding a map file. This was done to unconstrain individuals that were pre-assigned to a given population. The unrooted tree is oriented to show Pacific and Atlantic populations. Major branch nodes are labeled with percent bootstrap support (in black) and local branch ASTRAL quartet scores (Zhang et al. 2020; red values in parentheses). Each individual genotype (five letter illumina library code for identification) is colored by its collection site. For further details and references see Supplementary Note 3. Numbers indicate the outcome of a quartet analysis of incomplete lines sorting (Zhang et al. 2020).

Zhang, C., Scornavacca, C., Molloy, E. K. & Mirarab, S. (2020). ASTRAL-Pro: quartet-based species-tree inference despite paralogy. *Molecular biology and evolution, 37*(11), 3292-3307. doi:10.1093/molbev/msaa139

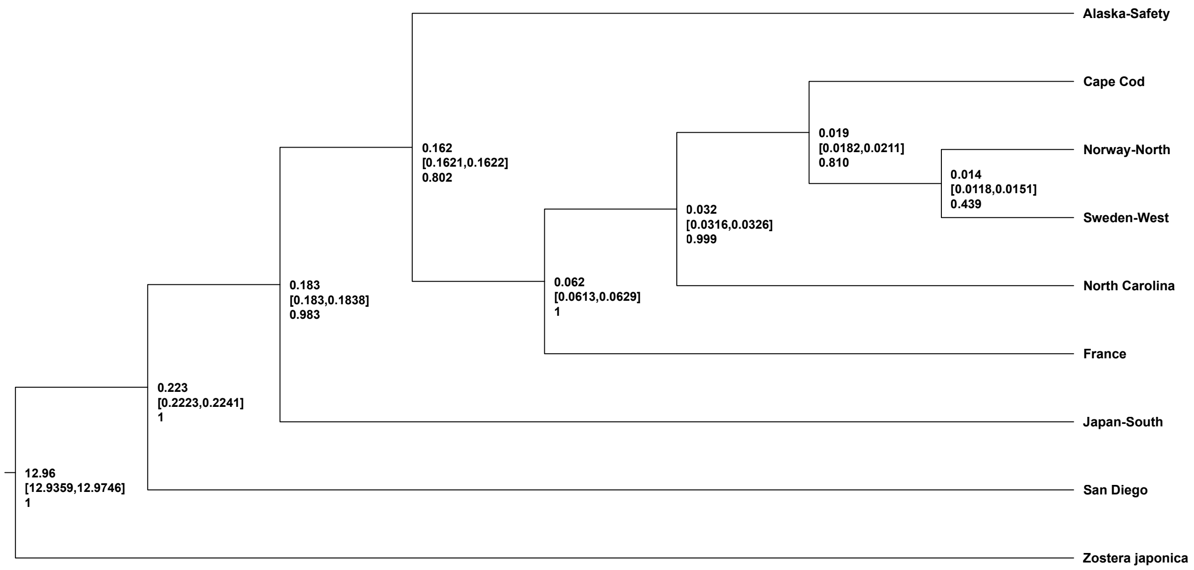

Supplementary Fig. 12: StarBEAST2 phylogenetic tree including divergence time estimation

The XML file was generated using BEAUTi from 70 randomly selected alignments from a subset of 4 samples per population. Parameters used in the StarBEAST2 analysis were: gene ploidy = 2; constant population sizes; population size parameter = 0.03 (assuming N_e = 10,000 and generation time 3 yrs); Gamma site model; estimated substitution rate; HKY substitution model; with estimated kappa parameter; empirical nucleotide frequencies; strict molecular clock; estimated clock rate; Yule model; Outgroup=*Z. japonica*; outgroup divergence time constrained with a lognormal prior [M=11.01; S=0.01; mean in real space, use originate]; MCMC chain length = 200,000,000; store every 200,000; pre-burnin = 0. StarBEAST2 was run in triplicate, inspecting each run with Tracer to check for model convergence (10% burn-in; Effective sample sizes [ESS]>300). TreeAnnotator was used to summarize each run (10% burn-in, median peak height, 0.5 posterior probability limit) which were then combined using LogCombiner. Tree has been transformed into a cladogram (uniform branch lengths) to increase visibility among nodes. Each node is labeled with: estimated median divergence time in millions of years, 95% confidence interval for median divergence times, and posterior probability, respectively (top to bottom). Subpopulation abbreviations: ASL- Alaska-Safety; MA- Cape Cod; NN- Norway North, SW- Sweden West; NC- North Carolina; FR- France; JS- Japan South; SD - San Diego. For further details and references see Supplementary Note 2.
