# Supplementary material for: Ocean current patterns drive the worldwide colonization of eelgrass (Zostera marina)
Source: Nat Plants. 2023 Jul 20;9(8):1207–20. doi: 10.1038/s41477-023-01464-3 (PMC10435387; doi:10.1038/s41477-023-01464-3)
Supplement: Supplementary file 2 — Reporting Summary [file 41477_2023_1464_MOESM2_ESM.pdf]

nature portfolio

Corresponding author(s): Thorsten B.H. Reusch

Last updated by author(s): May 22, 2023

Reporting Summary

Nature Portfolio wishes to improve the reproducibility of the work that we publish. This form provides structure for consistency and transparency in reporting. For further information on Nature Portfolio policies, see our Editorial Policies and the Editorial Policy Checklist.

Statistics

For all statistical analyses, confirm that the following items are present in the figure legend, table legend, main text, or Methods section.

n/a	Confirmed	
☐	☒	The exact sample size (*n*) for each experimental group/condition, given as a discrete number and unit of measurement
☒	☐	A statement on whether measurements were taken from distinct samples or whether the same sample was measured repeatedly
☐	☒	The statistical test(s) used AND whether they are one- or two-sided<br>*Only common tests should be described solely by name; describe more complex techniques in the Methods section.*
☒	☐	A description of all covariates tested
☒	☐	A description of any assumptions or corrections, such as tests of normality and adjustment for multiple comparisons
☒	☐	A full description of the statistical parameters including central tendency (e.g. means) or other basic estimates (e.g. regression coefficient) AND variation (e.g. standard deviation) or associated estimates of uncertainty (e.g. confidence intervals)
☐	☒	For null hypothesis testing, the test statistic (e.g. *F*, *t*, *r*) with confidence intervals, effect sizes, degrees of freedom and *P* value noted<br>*Give P values as exact values whenever suitable.*
☐	☒	For Bayesian analysis, information on the choice of priors and Markov chain Monte Carlo settings
☒	☐	For hierarchical and complex designs, identification of the appropriate level for tests and full reporting of outcomes
☒	☐	Estimates of effect sizes (e.g. Cohen's *d*, Pearson's *r*), indicating how they were calculated

Our web collection on statistics for biologists contains articles on many of the points above.

Software and code

Policy information about availability of computer code

Data collection

Reference genome
- Zostera marina v3.1 NCBI BioProject PRJNA701932
- annotated version of Zostera marina v3.1 at https://bioinformatics.psb.ugent.be/orcae/

Mapping of short reads against reference genome
- sequence reads were mapped against the chromosome-level reference genome of Zostera marina V3.1 using BWA MEM (Burrows-Wheeler Alignment Tool v0.1.17).
- alignments were converted to BAM format and sorted using Samtools
- MarkDuplicates module in GATK4 was used to identify duplicate reads (repository for GATK4 package at https://github.com/broadinstitute/gatk)
- calculation of mapping coverage and rate for each genotype using Samtools v1.11 (Supplementary Data 1 and 2)

Calling of single nucleotide polymorphism
- HaplotypeCaller (GATK4 v4.1.1.0) was used to generate a GVCF format file for each sample, GVCF files were combined by CombineGVCFs (GATK4)
- GenotypeGVCFs (GATK4) was used to call genetic variants.
- BCFtools (v1.11) was used to remove SNPs within 20 base pairs of an indel or other variant type (cf. Supplementary Fig. 1)
- VariantsToTable (GATK4) was used to extract INFO annotations
- quality filtering: marking by VariantFiltration (GATK4) accd. to the criteria MQ < 40.0; FS > 60.0; QD < 10.0; MQRandSum > 2.5 or MQRandSum < -2.5; ReadPosRandSum < -2.5; ReadPosRandSum > 2.5; SOR > 3.0; DP > 10804.0 (2 * average DP).
- those SNPs were excluded by SelectVariants (GATK4)

Data analysis

Excluding duplicate genotypes (i.e. clonemates) based on shared heterozygosity
-custom-made script at https://github.com/leiyu37/Detecting-clonemates.git

Identifying synonymous and non-synonymous SNPs in coding sequences of nuclear genome
-annotation of each SNP using SnpEff v4.3 (http://pcingola.github.io/SnpEff/)

cp_genome basic analysis
- genome de novo assembly using NOVOPlasty v3.8.2.

Quantifying population-level nuclear genomic diversity
-nucleotide diversity (phi) using vcftools (at https://vcftools.github.io/index.html)
-chromosomal-level individual heterozygosity custom Python3 script at github.com/leiyu37/populationGenomics_ZM.git

Assessing population structure
- Bayesian inference of population structure using the package STRUCTURE v. 2.3.4. available at https://web.stanford.edu/group/pritchardlab/structure.html
- Principal Component Analysis PCA: package vcfR to load the VCF format file, R-studio function glPca in adegenet package to conduct PCA analyses, visualization through the ggplot2 package
- analysis of conflicting phylogenetic signals using SplitsTree4 with subsequent NeighborNet algorithm to construct trees
- pairwise population differentiation via F_st using R-package StAMPP

Analysis of introgression
- calculation of Patterson's D-statistics using D-suite, vizualization of results using custom script available at https://github.com/mmatschiner/tutorials/blob/master/analysis_of_introgression_with_snp_data/src/plot_d.rb

SNAPP coalescent analysis
- Preparation of input file: custom made script at https://github.com/mmatschiner/snapp_prep
- Obtaining independent SNP loci by maintaining a minimal physical distance on genome: Vcftools v0.1.15
- SNAPP analysis within the Beast2 package available at https://www.beast2.org/snapp/

ASTRAL & StarBEAST2 coalescent analysis
Identifying core and variable genes

- de novo assembly of Illumina short reads for each sample using HipMer (https://portal.nersc.gov/project/hipmer/)
- alignment of primary transcript sequences (gene models) from the Z. marina reference (V3.1) using BLAT using default parameters to each de novo assembly
- classification of genes to be present if the transcript aligned with either (i) >60% identity and >60% coverage from a single alignment, or (ii) >85% identity and > 85% coverage split across three or fewer scaffolds using custom-made script, available at https://github.com/leiyu37/populationGenomics_ZM/tree/main/gene_presense_absence_analysis
- conversion of individual presence-absence-variation (PAV) calls into a matrix to classify genes into core, cloud, and shell categories based on their observation across the population using custom-made script, available at https://github.com/leiyu37/populationGenomics_ZM/tree/main/gene_presense_absence_analysis

Estimation of the rate of the molecular clock via divergence time estimation between Z. marina and Z. japonica

- identification of 1,072 syntenic paralog pairs in the Z. marina reference V3.1 via comparative genomics pipeline GENESPACE v0.9.4
- constrained protein homology search was performed using BLAT (v.30) and GeMoMa v1.7
- alignment of Z. marina transcripts to the Z. japonica genome assembly using BLAT alignment (v.30)
- extraction of each transcript best hit location (+500 bp sequence buffer) from Z. japonica genome via GeMoMa v1.7. protein prediction
- extraction of each peptide coding sequence via Gffread
- identification of 1:1 orthologs between Z. japonica and Z. marina using best reciprocal BLAT (v.30) hits
- calculation of fhe 4DTv rate among orthologs using 10,000 bootstrap estimates (0.0795- 0.0816; 90% CI) using a custom-made script

Constructing an ASTRAL species tree

- de novo assembly of Illumina short reads for each sample using HipMer
- alignment of transcript sequences from Z. marina (v3.1) against each HipMer assembly using BLAT (v30)
- prediction of CDS and protein sequences from each transcript alignment using GeMoMa (v1.7)
- construction of gene trees by aligning CDS sequences together using MAFFT (v7.475) (parameters: mafft --localpair --phylipout --maxiterate 1000)
- generating individual gene trees with IQTREE (v2.1.2) (parameters -B 1000 -m K2P -T auto)
- tree analysis using all gene trees jointly via a species tree analysis using ASTRAL (Zhang et al. 2018) v5.7.3
- population tree estimation using ASTRAL v5.7.3 without map file to unconstrain individuals that were pre-assigned to a given population.
- calculation of ASTRAL quartet scores accd. to Zhang et al. 2020 Mol Biol Evol ; using the ASTRAL-pro package v5.7.3

Divergence time estimates using StarBEAST2
- obtaining gene alignments by MAFFT as outlined in the ASTRAL species tree section
- estimation of divergence among Z. marina populations using StarBEAST2 (v2.6.3) (Bouckaert et al. 2014; Heled and Drummond 2010)
- prediction of protein sequences from an assembly of Zostera japonica (Xiaomei Zhang pers. comm., available at doi.org/10.6084/m9.figshare.21626327.v1, and Zhang et al. 2019) using the GeMoMa pipeline
- alignment and identification of reciprocal best orthologs of 8,687 predicted peptide sequences to Z. marina peptides using BLAT [v30]
- generation of xml file based on MAFFT alignments (phylip format) using seqmagick (v0.6.2; Shen et al. 2016) to convert phylip format to nexus
- generation of xml file required for StarBEAST2 using BEAUTi v2.6.0

- divergence estimation using StarBEAST2 with parameters: gene ploidy = 2; constant population sizes; population size parameter = 0.03; Gamma site model with estimated substitution rate; HKY substitution model; estimate kappa; empirical nucleotide frequencies; strict molecular clock; estimated clock rate; Yule model; Outgroup= Z. japonica ; Outgroup divergence constrained with a lognormal prior [M=11.01; S=0.01; mean in real space, use originate]; MCMC chain length = 200,000,000; store every 200,000; pre-burnin = 0.
- checking of model convergence of StarBEAST2 Tracer v1.7.1 to check for model convergence (Barido-Sottani et al. 2018) (20% burn-in; Effective sample sizes [ESS]>300)
- summary of each run using TreeAnnotator v2.6.0 (20% burn-in, median peak height, 0.5 posterior probability limit) which were then combined using LogCombiner v2.6.0
- re-summarizing the log combined tree file with TreeAnnotator v2.6.0 using the parameters listed above

Historical demography using MCMS - multiple sequentially Markovian coalescent
-generation of mappability mask file for each of six chromosomes using SNPable (http://lh3lh3.users.sourceforge.net/snpable.shtml)
-generation of ramet-specific mask file based on bam file using bamCaller.py (https://github.com/stschiff/msmc-tools)
-running the demographic history analysis with msmc_1.1.0_linux64bit

cpDNA haplotype analysis:
-haplotype network: computed as median Joining Network method with epsilon 0 and 1 implemented by PopART v1.7 (https://popart.maths.otago.ac.nz/how-to-cite/)
-maximum-likelihood phylogenetic tree using software iQ-Tree v1.5.5 (Nguyen et al. 2015) with 1000 bootstrap runs

For manuscripts utilizing custom algorithms or software that are central to the research but not yet described in published literature, software must be made available to editors and reviewers. We strongly encourage code deposition in a community repository (e.g. GitHub). See the Nature Portfolio guidelines for submitting code & software for further information.

Data

Policy information about availability of data

All manuscripts must include a data availability statement. This statement should provide the following information, where applicable:

- Accession codes, unique identifiers, or web links for publicly available datasets
- A description of any restrictions on data availability
- For clinical datasets or third party data, please ensure that the statement adheres to our policy

Short reads for all 190 samples have been deposited on Genbank short read archive, all relevant SRA accession numbers can be found in Supplementary Data Table 3
VCF files of the called SNPs have been deposited on figshare (doi.org/10.6084/m9.figshare.21629471.v1)
Coding sequences of Z. japonica and Z. marina for the ASTRAL analysis can be found on figshare (doi.org/10.6084/m9.figshare.21626327.v1)
The short sequence coverage for each sample is given in Supplementary Data 1, the respective mapping rate can be found in Supplementary data 2, SRA acc nos and library specifications are listed in Supplementary Data 3.
Source Data for Fig 1b,c, are given in the file Source Data Fig1b,c,

Human research participants

Policy information about studies involving human research participants and Sex and Gender in Research.

Reporting on sex and gender	na
Population characteristics	na
Recruitment	na
Ethics oversight	na

Note that full information on the approval of the study protocol must also be provided in the manuscript.

Field-specific reporting

Please select the one below that is the best fit for your research. If you are not sure, read the appropriate sections before making your selection.

☐ Life sciences ☐ Behavioural & social sciences ☒ Ecological, evolutionary & environmental sciences

For a reference copy of the document with all sections, see nature.com/documents/nr-reporting-summary-flat.pdf

Ecological, evolutionary & environmental sciences study design

All studies must disclose on these points even when the disclosure is negative.

Study description	A population genomics study using full resequenced nuclear and chloroplast genomes of the marine angiosperm (or seagrass)

Study description	Zostera marina was conducted in order to identify worldwide patterns of genetic diversity, reconstruct historical demography and date major colonization events starting from the putative origin in the Northwest Pacific.
Research sample	A research sample is a leaf shoot (or ramet) of the seagrass Zostera marina (=eelgrass), 12 of which were collected at each site, a site being representative of a local population
Sampling strategy	Sixteen worldwide locations of eelgrass (Zostera marina) were selected based on prior microsatellite based studies of population structure, with the general objective to cover the entire distribution range on both sides of Pacific and Atlantic (Fig. 1a). At each selected site, 12 specimen were collected for population genomic analysis using wading, snorkling or diving.
Data collection	Collectors are mentioned in the section on sampling permits. Sample extraction was performed at GEOMAR Kiel (Diana Gill) and at UC Davis (Brenda Cameron), extracts were then sent to JGI. Data acquisition in terms of genome re-sequencing was done at the U.S. Department of Energy Joint Genome Institute (https://ror.org/04xm1d337), a DOE Office of Science User Facility, supported by the Office of Science of the U.S. Department of Energy operated under Contract No. DE-AC02-05CH11231
Timing and spatial scale	Sampling was conducted between May 2016 and August 2017. At a given site, a population was defined as continguous eelgrass meadow of at least 50 m across (parallel to shore)
Data exclusions	For the population genomic analysis, inadvertently sampled clone mates (=members of the same seagrass genet at particular sites were excluded (cf. Supplementary Table 3), as well as those originating from within-clone selfing (Supplementary Table 2).
Reproducibility	Three independent approaches were used to date major phylogenetic branching events. Two of those used nuclear (SNP) data, namely the coalsecent analysis via SNAPP, and the gene based coalsecence analysis using ASTRAL in combination with StarBEAST2 analysis (Supplementary Note 3). A third approach used the mutational distance between complete chloroplast haplotypes in combination with a universal average molecular clock derived from several monocotyledoneous angiosperms (Supplementary Note 4).
Randomization	Specimen at each site were taken haphazardly, at minimal distances of 5m, along a transect parallel to the shore line
Blinding	no blinding was possible

Did the study involve field work? ☒ Yes ☐ No

Field work, collection and transport

Field conditions	As our study builds upon genome polymorphism and differentiation that was emerging over hundreds to thousands of years, no environmental data were collected at the time of sampling
Location	All 16 sampling locations were geo-referenced, coordinates are listed in Supplementary Table 1 and below
Access & import/export	Sampling took place between May 2016 and August 2017. For all sites, sampling permits have been obtained by the relevant national or regional authorities where required. An e-mail string can be provided upon request between the local collaborators and the respective national authorities with respect to an obligation or waiver of CBD or general sampling permit.<br><br>Specific information is listed below for each site, from West to East (site abbreviations as in Fig 1a and Supplementary Table 1):<br>- Japan North / JN /Pos 43.021N 144.903E. Sampling: collecting permit to Dr. Massa Nakaoka (in Japanese). CBD-"Nagoya": collection in August 2017 before implementation of CBD access regulation in Japan<br>- Japan South /J S /Pos 34.298N 132.916E. Sampling: collecting permit to Dr. Masakazu Hori, CBD-"Nagoya": see above<br>- Alaska Safety Lagoon, USA /ASL / Pos 64.485N 164.762W. Sampling: no collecting permit required, waiver by U. S. Fish and Wildlife Service to Dr. David Ward & Dr. Sandra Talbot, CBD: non-signatory<br>-Alaska- Izembek Lagoon, USA /ALI / Pos 55.329N 162.821W. Sampling: no collecting permit required, waiver by U. S. Fish and Wildlife Service to Dr. David Ward & Dr. Sandra Talbot. CBD-"Nagoya": non-signatory<br>-Willapa Bay, Washington State, USA / WAS / Pos 46.474N 124.028W. Sampling: permit to Dr. Jennifer Ruesink through Wash Dept Natural Res. CBD-"Nagoya": non-signatory<br>-Bodega Bay, USA / BB /Pos 38.320N 123.055W. Sampling: permit to Dr. John S Stachowicz through Dept Fish Wildlife CA. CBD-"Nagoya": non-signatory<br>-San Diego Bay, USA / SD / Pos 32.714N 117.225W. Sampling: permit to Dr. Kevin A Hovel through Dept Fish Wildlife CA. CBD-"Nagoya": non-signatory<br>-Quebec, Canada / QU / Pos 49.112N 68.176W. Sampling: permit to Dr. Mathieu Cusson through Fisheries and Oceans Canada. CBD-"Nagoya": non-signatory<br>-Massachusetts, USA / MA/ Pos 42.420N 70.915W. Sampling: permit to Dr. Randall Hughes through Massachusetts Division of Marine Fisheries. CBD-"Nagoya": non-signatory<br>-North Carolina, USA / NC / Pos 34.692N 76.623W. Sampling: permit to Dr. Joel Fodrie through North Carolina Division of Marine Fisheries. CBD-"Nagoya": non-signatory<br>-Røvika, Northern Norway / NN / Pos 67.268N 15.257E. Sampling: no permit required. CBD-"Nagoya": waiver<br>-Torserød, West Coast of Sweden / SW / 58.313N 11.549E. Sampling: no permit required, waiver by Administrative County Board of Västra Götalands to Dr. Per-Olav Moksnes. CBD-"Nagoya": waiver<br>-Port Dinllaen, Wales, UK / WN/ 52.991N 4.450W. Sampling: waiver to Dr. Richard Unsworth by authorities as amount negligible. CBD-"Nagoya": waiver /collection before 1 July 2017<br>-Ria Formosa, Portugal /PO / 37.040N 7.910W sampling: no collection permit required. CBD-"Nagoya": collection before 1 July 2017<br>-Thau Lagoon, France / FR/ 43.447N 3.662E sampling: no collection permit required, waiver to Dr. Francesca Rossi. CBD-"Nagoya":

	waiver /collection before 1 July 2017<br>-Adriatic Sea, Croatia /CZ /Pos 44.212N 15.491E. sampling: no collection permit required, waiver to Dr. Stewart Schulz & Dr. Claudia Kruschel. CBD-"Nagoya": non-signatory
Disturbance	At each site, in an area of several 1000 m2, 12 leaf shoots of eelgrass were collected, representing <0.001% of all plants of the respective meadow. This level of disturbance is negligible compared to, for example, natural physical disturbance by storms or herbivory

Reporting for specific materials, systems and methods

We require information from authors about some types of materials, experimental systems and methods used in many studies. Here, indicate whether each material, system or method listed is relevant to your study. If you are not sure if a list item applies to your research, read the appropriate section before selecting a response.

Materials & experimental systems

n/a	Involved in the study
☒	☐ Antibodies
☒	☐ Eukaryotic cell lines
☒	☐ Palaeontology and archaeology
☒	☐ Animals and other organisms
☒	☐ Clinical data
☒	☐ Dual use research of concern

Methods

n/a	Involved in the study
☒	☐ ChIP-seq
☒	☐ Flow cytometry
☒	☐ MRI-based neuroimaging
